# Effect of Hormonal Contraceptives on Circulating Biomarkers of Inflammation, Chemotaxis, Angiogenesis, and Vascular Stress

**DOI:** 10.1111/apm.70235

**Published:** 2026-07-14

**Authors:** Bertram Kjerulff, Khoa Manh Dinh, Jens Kjærgaard Boldsen, Ulrik Schiøler Kesmodel, Joseph Dowsett, Susan Mikkelsen, Lisette J. A. Kogelman, Maria Didriksen, Christina Mikkelsen, Jakob Bay, Mie Topholm Bruun, Bitten Aagaard, Mette Nyegaard, Klaus Rostgaard, Erik Sørensen, Ole Birger Pedersen, Sisse Rye Ostrowski, Christian Erikstrup

**Affiliations:** ^1^ Department of Clinical Immunology Aarhus University Hospital Aarhus Denmark; ^2^ Big Data Centre for Environmental Research and Health, Aarhus University Aarhus Denmark; ^3^ Department of Clinical Immunology Copenhagen University Hospital, Rigshospitalet Copenhagen Denmark; ^4^ Department of Clinical Medicine Faculty of Health, Aarhus University Aarhus Denmark; ^5^ Kvindesygdomme Og Fødsler, Gødstrup Hospital Herning Denmark; ^6^ Department of Obstetrics and Gynecology Aalborg University Hospital Aalborg Denmark; ^7^ Department of Health Science and Technology Genomic Medicine Group, Aalborg University Aalborg Denmark; ^8^ Department of Neuroscience Faculty of Health and Medical Science, Copenhagen University Copenhagen Denmark; ^9^ Department of Clinical Immunology Zealand University Hospital Køge Denmark; ^10^ Department of Clinical Immunology Odense University Hospital Odense Denmark; ^11^ Department of Clinical Immunology Aalborg University Hospital Aalborg Denmark; ^12^ Department of Epidemiology Research Statens Serum Institute Copenhagen Denmark; ^13^ Danish Cancer Institute Danish Cancer Society Copenhagen Denmark; ^14^ Department of Clinical Medicine Faculty of Health and Medical Sciences, University of Copenhagen Copenhagen Denmark

## Abstract

Hormonal contraceptive (HC) use is known to be associated with elevated CRP levels. However, evidence on the effect of HC on several circulating inflammatory biomarkers in healthy humans is limited. The aim of this study was to examine the association between hormonal contraceptives and circulating biomarker levels and differential cell counts. A cross‐sectional observational study design with biomarker measurements from 4,160 healthy females was used, coupled with registered HC prescriptions. We compared 752 high and low dose combined oral contraceptive (COC) users, 77 progestin‐only pill users, 417 levonorgestrel intrauterine device users, 65 hormonal therapy users, and 1,523 post‐menopausal women with a control group of 1,326 women below 50 years. Repeated differential cell counts from a different dataset of 16,231 females were analyzed for the effects of HC. Data were analyzed using various regression models. COC use was associated with higher vascular marker levels but lower chemokine levels. We found no association with progestin‐only use. The association with differential cell counts was limited for most HC types. This study emphasizes the widespread impact of HC on the immune and vascular systems and how the impact may differ between HC types. The biomarker associations found could be targets for elucidation in functional studies.

## 1 Introduction

Hormonal contraceptives (HC) have been widely used since their introduction in 1960 [1]. In Denmark, half of all women aged 15–19 years use HC, increasing to 60% among women aged 20–24 years, and decreasing gradually to 23% in the 45–49 years age group. Women younger than 30 years mainly use combined oral contraceptives (COC), whereas levonorgestrel‐intrauterine device (LNG‐IUD) is preferred among women aged 30–49 years [2]. HC use in Denmark generally decreased, and IUD use increased from 2010 to 2019 (available at medstat.dk/en), aligning with trends observed in the US and Sweden [2, 3, 4].

We have shown that C‐reactive protein (CRP) levels are increased among COC users and COC use is a stronger determinant of elevated CRP than lifestyle factors such as high body mass index (BMI) and smoking [5]. Whether this CRP increase reflects actual low‐grade inflammation (LGI) and whether it is associated with an increase in cardiovascular disease (CVD) risk is unclear. CRP is primarily released in response to IL‐6 during infection, tissue damage, inflammatory diseases, and cancer [6, 7, 8]. In addition, LGI is associated with increased risk of CVD [9]. COC use was associated with higher CRP, corresponding to LGI levels, among Danish blood donors.

The well‐known effect of COC on CRP does not appear to be driven by increased IL‐6 [5, 10, 11]. Studies have shown both increased and decreased risks of various autoimmune diseases among HC users [12] and have linked HC use to inflammatory markers [13, 14, 15]. However, extensive analysis of inflammatory markers in large cohorts of healthy HC users is lacking. An improved understanding of which inflammatory markers are associated with HC use may help explain observed side effects and disease associations. Healthy Blood donors offer a valuable opportunity to investigate the isolated effects of hormonal contraceptive use on inflammatory markers.

In this study, we aimed to examine the effect of specific types of HC on the concentration of 47 circulating biomarkers of inflammation, chemotaxis, angiogenesis, and vascular stress in a large population‐based cohort of Danish blood donors.

## 2 Methods

### 2.1 Study Population and Design

**FIGURE 1:**
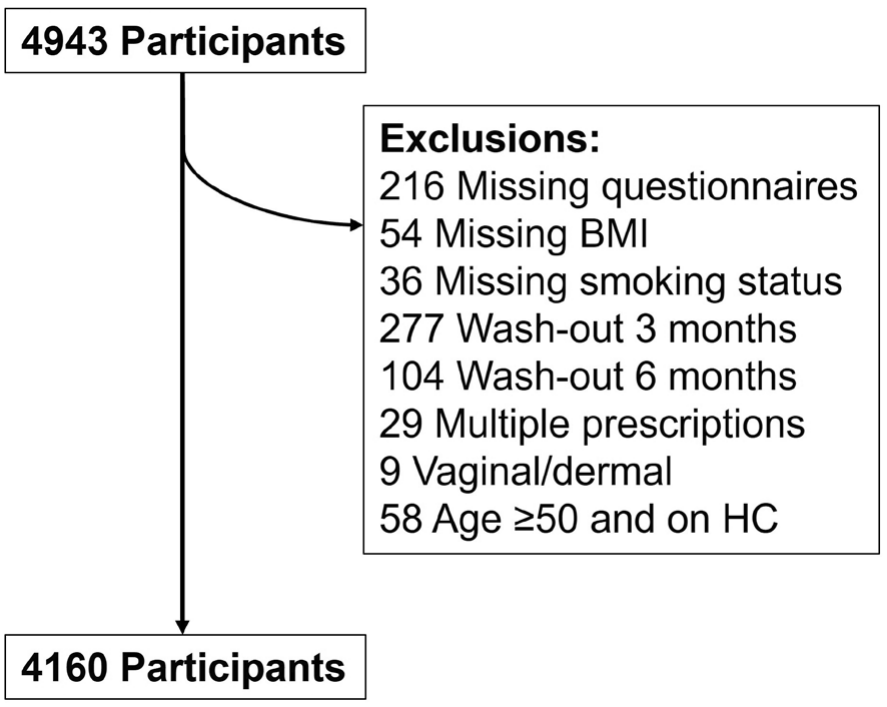
Participant exclusions prior to analysis.

This cross‐sectional observational study included Danish Blood Donor Study (DBDS) participants enrolled from 2010 to 2021. DBDS is an ongoing, multicenter cohort study hosting a large biobank. Briefly, over 175,000 consented donors completed a health questionnaire and had a gel‐separated EDTA plasma sample stored at −20°C. Donors allowed biobank use of all previous and subsequent donations and linkage to Danish registers [16]. In this particular study, we included females from the *DBDS 10 K Inflammatory Biomarker Cohort* [17]. Concentrations of 47 plasma biomarkers were available for 4943 participants from this *DBDS 10 K Inflammatory Biomarker Cohort* (Figure 1). After the exclusions, summarized in Figure 1, measurements from 4160 participants were available. Furthermore, in the full DBDS cohort, repeated differential cell counts (white blood cells, lymphocytes, monocytes, basophils, neutrophils, and eosinophils) were available from a separate but overlapping sub‐cohort of 16,231 female DBDS participants younger than 50 years.

### 2.2 Inflammatory Markers and Differential Cell Counts

Biomarkers were measured using Mesoscale Discovery (Meso Scale Diagnostics LLC, Maryland, USA) V‐plex kits following the manufacturer's recommendations. The panels used were: Proinflammatory Panel 1, Cytokine Panel 1, Cytokine Panel 2, Angiogenesis Panel 1, Chemokine Panel 1, and Vascular Injury Panel 2. Panels were chosen to cover a broad range of inflammatory and vascular markers. The selection, measurements, and quality control are published elsewhere [17]. A description of biomarker measurements can be found in the supplementary methods file.

Biomarkers were grouped by function or associated cell types. The groups are displayed in Table 1 and have been used previously for DBDS data [17]. Common biomarker aliases are listed in the supplementary methods.

**TABLE 1:** The biomarker grouping.

Group	Biomarkers
Proinflammatory	CRP, SAA, TNF‐α, TNF‐β, IL‐1α, IL‐1β, IL‐1RA, IL‐3, IL‐6, IL‐12/IL‐23p40, IL‐12p70, IL‐15
T cell‐derived	IFN‐γ, IL‐2, IL‐4, IL‐5, IL‐9, IL‐10, IL‐13, IL‐16, IL‐17A, IL‐17A/F, IL‐17B, IL‐17C, IL‐17D
Chemokines	CCL11, CCL26, CXCL8/IL‐8, CXCL10, CCL2, CCL13, CCL22, CCL3, CCL4, CCL17
Growth factors and vascular markers	bFGF, Flt‐1, Tie‐2, IL‐7, PlGF, VEGF‐A, VEGF‐C, VEGF‐D, TSLP, GM‐CSF, sICAM‐1, sVCAM‐1

*Note:* The biomarkers placed in each of the four groups based on effect or origin.

To further examine immune activation, we linked the nearest answered questionnaire and applied the same exclusion criteria as in Figure 1 to routine differential cell count measurements from blood and plasma donations among female DBDS participants in the Central Denmark Region (2010–2022). Cell counts were measured using Sysmex XT‐1800i or XN‐1000 (Kobe, Japan). After 16,231 participants contributed up to 115,248 measurements, as shown in Table S1. Exposure groups were defined as below, but were limited to non‐users, COC, IUD, and POP.

### 2.3 Exposure Definition of Contraceptive Use

HC use was identified from the national prescription register (NPR) using anatomical therapeutic chemical classification (ATC) codes defined in the supplementary methods file. The NPR holds all filled prescriptions from Danish pharmacies coded by ATC and also holds information on the quantity of the prescriptions used to define the exposure period [18, 19].

Participants were grouped as follows: Non‐users below 50 years or above (postmenopausal group), and hormonal contraceptive users below 50 years. HC users were sub‐grouped into: COC‐low: Low estrogen, less than 30 μg/dose; COC‐high: High estrogen, 30 μg/dose or more; vaginal/dermal: Combination, vaginal/dermal; LNG‐IUD: Levonorgestrel intra‐uterine device (progestin‐only); POP: Progestin‐only pill; OC users; and hormone therapy (HT): Menopause‐related hormonal treatment patients (this group also includes participants aged more than 50 years).

The few users of combination vaginal rings or dermal implants were excluded. Progestin‐only depot and transdermal implants were included in the POP group. The first DBDS questionnaire with responses from 40,652 female participants, collected from 2010 to 2015, identified 50 years of age to be the median menopausal age, which was then chosen as the cut for menopausal age. LNG‐IUD exposure time was set to four years based on reported durations [20, 21]. Duration of use allowed a 14‐day gap between active prescriptions.

Recent users (cessation within 6 months) and participants with different overlapping HC prescriptions were excluded.

### 2.4 Other Variables

BMI was included as a continuous variable and calculated from self‐reported height and weight. Current smoking was self‐reported and included as a binary variable. Sample age was defined as the time from sample draw to analysis. Age was available from register data and was calculated at the time of sample draw. These were all included as they are associated with biomarker levels [17].

### 2.5 Statistics

**FIGURE 2:**
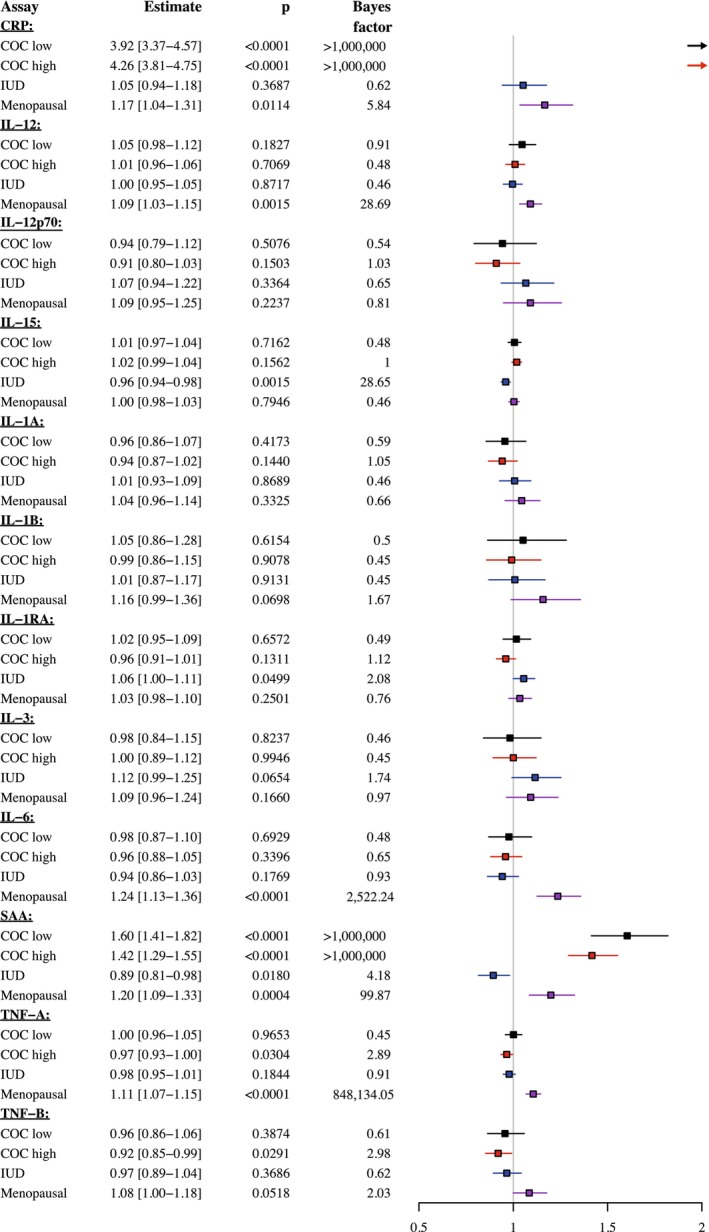
Effects of contraceptives on proinflammatory biomarkers. Fold change estimates in proinflammatory biomarkers in hormonal contraceptive users and menopausal women compared to the non‐users. Estimates are given with 95% confidence intervals in brackets. The Bayes factor column shows the calculated Bayes factors with values above 1,000,000 indicated by > 1,000,000. The arrows indicate the position of the CRP COC estimates beyond the axis limits.

**FIGURE 3:**
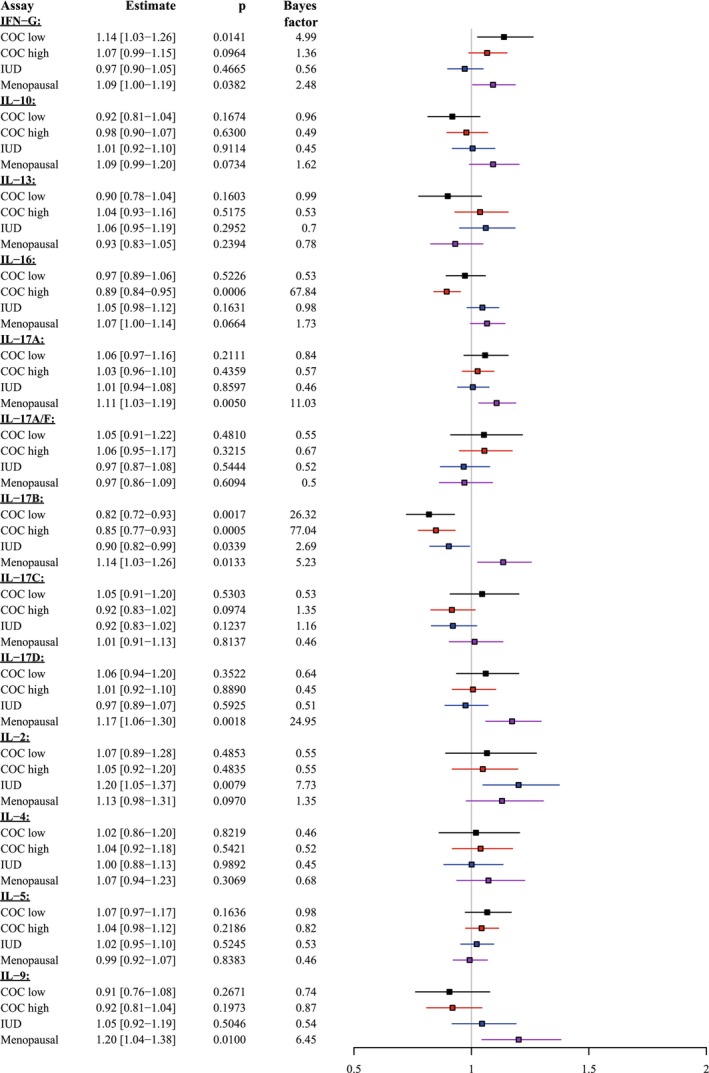
Effects of contraceptives on T cell‐derived biomarkers. Fold change estimates in T cell‐derived biomarkers in hormonal contraceptive users and menopausal women compared to the non‐users. Estimates are given with 95% confidence intervals in brackets. The Bayes factor column shows the calculated Bayes factors with values above 1,000,000 indicated by > 1,000,000.

**FIGURE 4:**
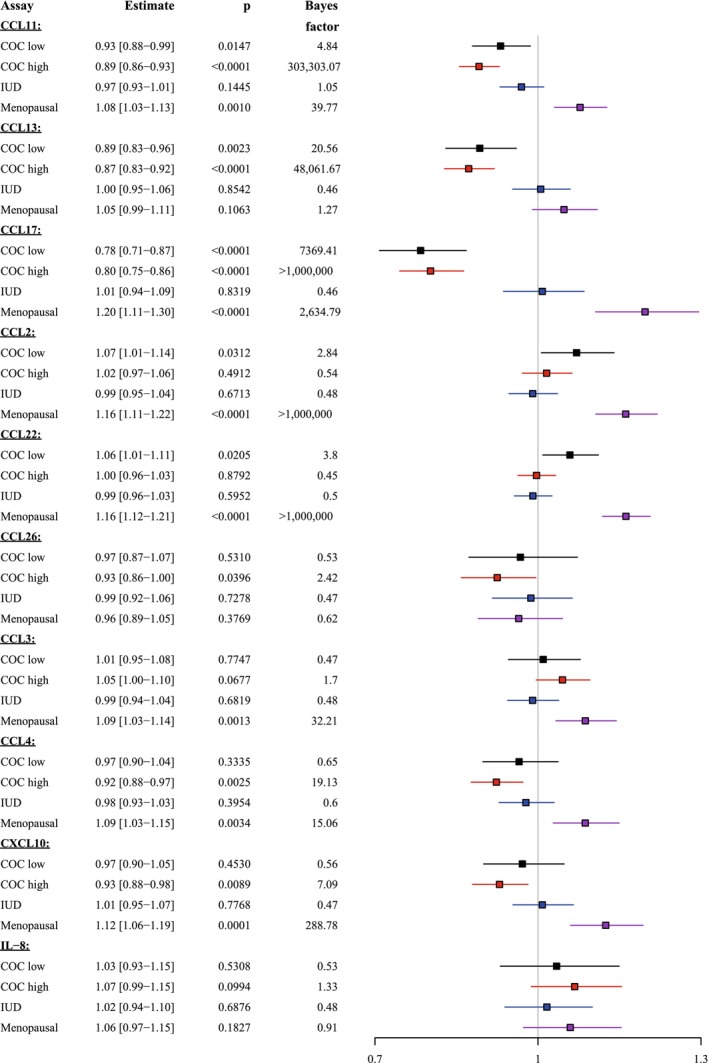
Effects of contraceptives on Chemokines. Fold change estimates in chemokine biomarkers in hormonal contraceptive users and menopausal women compared to the non‐users. Estimates are given with 95% confidence intervals in brackets. The Bayes factor column shows the calculated Bayes factors with values above 1,000,000 indicated by > 1,000,000.

**FIGURE 5:**
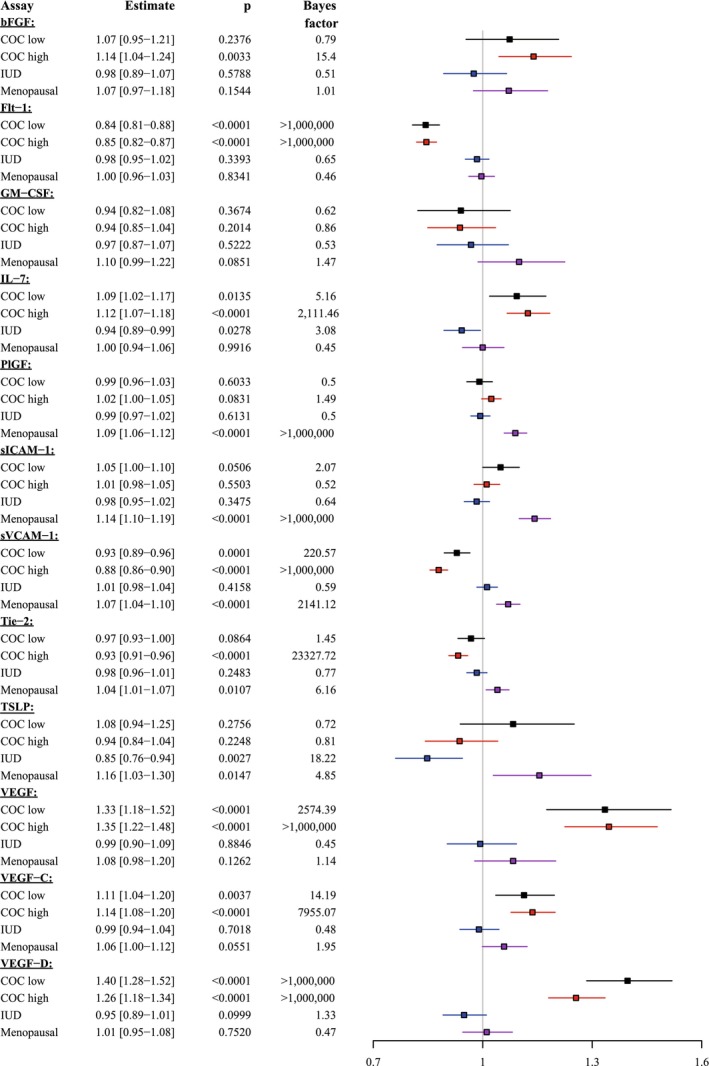
Effects of contraceptives on growth factor biomarkers. Fold change estimates in growth factor biomarkers in hormonal contraceptive users and menopausal women compared to the non‐users. Estimates are given with 95% confidence intervals in brackets. The Bayes factor column shows the calculated Bayes factors with values above 1,000,000 indicated by > 1,000,000.

Participant characteristics were presented as medians with interquartile ranges or counts with percentages. Associations between the HC group and log‐transformed biomarker concentrations were estimated using multivariable linear regression. Models were adjusted for age, current smoking, BMI, sample age, sample analysis date, and Danish administrative region of sampling. Only complete cases were included. Estimates with 95% confidence intervals (CI) were exponentiated to yield fold or percentage difference relative to the non‐user reference group. Duration of use was examined in two sub‐analyses, excluding users with less than 28 days of use, to allow a full cycle of effect. Cell count models were adjusted for age, BMI, and current smoking, with participant as a random effect, as participants had measurements from multiple dates. *p*‐values were Bonferroni corrected by multiplying by the number of tests in the relevant biomarker group and considered statistically significant when < 0.05. All Table S1 show the corrected *p*‐values, while Figures 2, 3, 4, 5 show the crude *p*‐values. POP and HT users were omitted from figures due to small numbers, but were included in Table S1. Figures also include a Bayes factor approximation based on model output as described by Rostgaard [22] using the EpiForsk package [23]. In simple terms, the Bayes factor presents an estimate of P (the alternative hypothesis is true)/P (the null hypothesis is true). Analysis was performed in R version 4 (R Foundation for Statistical Computing, Vienna, Austria, www.R‐project.org).

## 3 Results

### 3.1 Participant Characteristics

Characteristics of the 4160 participants are shown in Table 2. The COC users were younger, whereas the HT and menopausal groups were expectedly older than non‐users.

**TABLE 2:** Participant characteristics.

Group	COC low	COC high	LNG‐IUD	POP	HT	Menopausal	Non‐users
*n*	228	524	417	77	65	1,523	1,326
Age (years)	27 (23–31)	27 (23–33)	39 (34–43)	33 (25–40)	61 (57–63)	60 (54–63)	39 (32–45)
Current Smokers	30 (13.2%)	87 (16.6%)	58 (13.9%)	10 (13%)	6 (9.2%)	212 (13.9%)	177 (13.3%)
BMI (kg/m^2^)	23 (21–25)	24 (22–27)	24 (22–27)	23 (21–26)	24 (22–27)	25 (23–28)	24 (22–28)
Duration of HC use (months)	7 (3–14)	8 (3–17)	24 (13–36)	6 (2–17)	1 (0–2)	0 (0–0)	0 (0–0)
Sample age (years)	9 (8–10)	6 (3–9)	5 (3–9)	5 (3–9)	9 (4–10)	8 (4–10)	8 (4–10)

*Note:* Sample age is the time from sample draw to analysis. Counts are presented as *n* (%) and continuous variables as median (interquartile range [IQR]).

Abbreviations: COC high, high estrogen combined oral contraceptives; COC low, low estrogen combined oral contraceptives; HT, hormone therapy; LNG‐IUD, progestin‐releasing intrauterine device; Menopausal, non‐contraceptive users aged 50 years or above; POP, progestin‐only pill.

### 3.2 Biomarker Differences

Estrogen‐containing COC use was associated with higher CRP, SAA, VEGF‐A, VEGF‐C, VEGF‐D, bFGF, and IL‐7 for one or both dosage groups. COC use was associated with lower IL‐16, IL‐17B, Flt‐1, Tie‐2, sVCAM‐1, and multiple chemokines. LNG‐IUD use was associated with lower TSLP, IL‐15, and IL‐17B. Group‐specific results are shown in Figures 2, 3, 4, 5.

The menopausal group was associated with higher SAA, TNF‐α, IL‐12, IL‐17D, PlGF, sICAM‐1, sVCAM‐1, and multiple chemokines. No associations were observed in the 77 POP users, and they were omitted from Figures 2, 3, 4, 5. HT was associated with higher IL‐2, TNF‐α, CCL2, CCL22, and sICAM‐1. Fully adjusted results for all groups are shown in Table S2 and a simpler and unadjusted model is shown in Tables S3 and S4.

### 3.3 Differences Between High and Low Dose COC

Combining the high‐ and low‐dose COC groups revealed no additional differences (Table S5). No dose‐specific differences remained after multiple‐testing correction (Table S6).

### 3.4 Effects of Duration of Use

Duration of use was examined in two additional analyses, excluding users with less than 28 days of use (101 participants) and the HT group. Adjusted for duration of use, IL‐15 and TSLP were no longer significant in LNG‐IUD users, while IL‐16 was no longer lower in COC‐high users (Table S7).

When duration was the exposure, IL‐12 and IL‐17A negatively associated with longer COC‐high use, and IL‐5 was negatively associated with LNG‐IUD use (Table S8). When excluding those with less than 28 days of HC use made VEGF‐C in COC‐low and bFGF in COC‐high non‐significant (Table S9).

### 3.5 Never Users

To assess residual effects, we compared never‐users (no recorded prescriptions) with current users. Among 917 never‐users, only 247 were pre‐menopausal. Results (Table S10) aligned with the main analysis, but with fewer significant differences due to the reduced power. In the HT and menopausal group, there was now significantly higher CRP, but no longer a difference in several other markers.

Allowing one previous prescription yielded 309 non‐users (Table S11). CRP was not significantly higher in HT users, but there was significantly higher IL‐17A and sVCAM‐1.

### 3.6 Grouping by ATC Code

To examine specific contraceptives, the participants were grouped by the ATC code of their most recent prescription, excluding codes with fewer than 50 users. Most differences did not withstand correction for multiple testing (Figure S1 to Figure S4 and Table S12). COCs generally shared the same patterns, though G03AA11 had more noticeable associations in several markers. No differences were observed in G03AC09 (POP).

### 3.7 HT Users and Menopause

An additional analysis, with the same variables as the main analysis, was performed comparing the HT users with the menopausal group in Table S13. There was an association with HT use for IL‐3, which is unreliable because 70% of the measurements were below the detection limit.

### 3.8 Differential Cell Counts

Contraceptive use was associated with minor differences in the various cell counts (Table 3). The largest change was the 14% decrease in basophiles in low‐dose COC users, corresponding to around 6 cells per μL. For the remaining cell types, the largest observed difference was around 6%.

**TABLE 3:** The estimated percentage difference in cell counts for hormonal contraceptive users compared to non‐users.

	White blood cells	Neutrophils	Lymphocytes	Basophils	Monocytes	Eosinophils
**Mean cell count/μL**	6 849	3 928	2 172	41	548	161
**IUD**	**0.7 [0.3: 1.1]**	−0.4 [−1.1: 0.3]	**1.0 [0.5: 1.5]**	**3.7 [2.7: 4.8]**	**−1.0 [−1.5: −0.5]**	**2.7 [1.5: 4.0]**
**COC High**	**1.2 [0.8: 1.6]**	0.4 [−0.3: 1.2]	**3.6 [3.1: 4.2]**	**−4.1 [−5.2: −3.0]**	**−4.4 [−5.0: −3.8]**	**−6.0 [−7.3: −4.7]**
**COC low**	−0.5 [−1.1: 0.2]	−0.2 [−1.8: 1.3]	**3.7 [2.5: 4.9]**	**−14.2 [−16.3: −12.0]**	**−1.6 [−2.9: −0.3]**	**−5.6 [−8.3: −2.7]**
**POP**	0.3 [−0.5: 1.2]	−1.1 [−2.5: 0.3]	**1.7 [0.6: 2.8]**	**6.7 [4.5: 9.0]**	−0.1 [−1.2: 1.1]	−2.0 [−4.5: 0.6]

*Note:* % change estimates and 95% CI of cell concentration for each contraceptive type. Adjusted for age, smoking and BMI. Estimates with 95% CI not spanning zero are marked with bold text.

## 4 Discussion

This cross‐sectional study examined how HC associates with the concentration of 47 circulating biomarkers and differential cell counts in female blood donors. The cohort included 1246 HC users and 1326 non‐users. COC use was associated with lowered chemokine and varying growth factor levels, but few differences in classic proinflammatory markers, besides SAA and CRP, and an indication of higher IFN‐γ. This suggests that other factors lead to the observed and previously reported higher CRP among COC users [5] than a classical stimulation of the hepatic acute‐phase response pathway through IL‐1, −6, and TNF‐α. This study presents associations with COC use in healthy humans and on a wider range of inflammatory and vascular markers than previously reported. Although the exact mechanisms remain unclear, the pattern suggests COC‐related effects on immune and vascular pathways, particularly for chemokines and cell motility.

The differences in age and BMI between the contraceptive groups were expected, as COC users are generally younger, and BMI tends to increase with age. The slightly higher proportion of smokers in the COC high group may reflect the higher prevalence of smokers among the younger participants or could be incidental.

Association with higher VEGF but lower Flt‐1 (VEGF receptor) matches previous literature in COC users and estrogen‐related murine models [24, 25]. Estrogen has known preventive effects on atherosclerosis [26, 27]. Lower sVCAM‐1 in COC users and higher sICAM‐1 and sVCAM‐1 post‐menopause may be related to upregulation of antioxidative gene expression by estrogen [28, 29]. The higher VEGF also fits estrogen‐driven angiogenesis and endothelial cell proliferation [30, 31]. sVCAM‐1 is decreased in OC users [32], possibly reflecting reduced atherosclerosis in past OC users [33]. Overall, OC use has been associated with favorable endothelial effects despite known thrombotic risks [34]. Together, these findings indicate a shift in endothelial remodeling pathways. Such vascular changes may affect immunity, as atherosclerosis is considered an inflammatory disease [35]. Circulating VEGF has both been found to be stable and to vary during the menstrual cycle [36, 37, 38]. It is therefore likely that altering the cycle with HCs will then influence VEGF levels. IL‐7 was higher among COC users, consistent with estrogen having reported effects on the thymus [39, 40, 41].

Although there is a lack of studies in healthy humans, estrogen has been linked to higher IFN‐γ, and we observed a similar trend before correction [42, 43, 44]. Several chemokine shifts may reflect altered CCR4‐mediated recruitment [45, 46, 47]. The associations in these immune markers may relate to reported reductions in asthma risk among HC users [48]. The decrease in CCR3 binding chemokines (CCL11, 13, and 26) indicates a downregulation of the CCR3 axis, and an estrogen‐related decrease in activity of CCR3‐expressing basophils, eosinophils, and monocyte‐derived dendritic cells, possibly meaning less generation of reactive oxygen species and less inflammation [49, 50, 51, 52]. This matches the slightly lower eosinophile, monocyte, and basophile counts in COC users. CCR3 activation in vascular endothelial cells can have some of the same downstream effects as VEGF [53]. The lymphocyte count was higher in COC users, which has been reported for lymphocyte subsets [54]. Effects of hormonal contraceptives on cell counts were expected as immune cell distributions have been reported to change across the menstrual cycle [55]. Estrogen exposure has been found to increase CCL2 and CCL11 in murine cells [56] and CCL13 expression in synovial fibroblasts from arthritis patients [57]. This is contrary to the association with lower CCL13 in COC users. Lower CCL11 and 17 in estradiol users parallels our COC findings [58]. Estradiol‐related reductions in CCL4 in murine models are consistent with our observations [59], and both lower CCL4 and 17 further support reduced immune cell recruitment. Depot progestin users show altered mucosal cytokines, though these may not reflect systemic levels [60]. IL‐16, a proinflammatory chemoattractant, was also lower among COC high users. IL‐17B is inversely correlated with estrogen level in postmenopausal women [61] and was lower in COC users. These findings raise the question of whether COC users experience altered infection risk, as suggested in prior studies [62].

No POP associations were observed, but the 77 POP users limit statistical power. LNG‐IUD use was associated with lower TSLP and IL‐15. Although estrogen and IL‐1β stimulate TSLP secretion and TSLP in turn stimulates CCL2/MCP‐1 and IL‐8 secretion in endometrial stromal cells [63, 64], we found no LNG‐IUD effects on IL‐1β, CCL2, or IL‐8. Lower TSLP may reflect progesterone‐mediated reduction of estrogen receptors [65]. An IL‐15 increase with progesterone has been reported, although not in circulation [66, 67]. Minimal systemic changes in IUD users may reflect lower doses and predominantly local effects. IUD use has localized immune effects [68], but there is a paucity of studies on systemic effects. Other studies have also reported higher leukocyte counts but also altered metabolomic profiles in IUD users [69, 70]. As COC use declines and IUD use rises in Denmark, broader evaluation of HC side effects becomes increasingly important [2, 71, 72, 73].

HT users generally resembled the menopausal females, except for IL‐2. This likely reflects age and menopausal status rather than HT itself. Most HT products (gels or vaginal tablets) act locally, which may limit systemic effects.

Several chemokines involved in immune cell recruitment and chemotaxis were lower in COC users, although CCL2, CCL3, and CCL22 trended towards higher levels. This indicates effects on immune cell recruitment and motility. The effects on immune cells could influence infection risk, which can be examined in epidemiologic studies. The changes in VEGF and bFGF reflect known cardiovascular side effects of COC use, but do not explain the underlying mechanisms, as they are involved in angiogenesis. Both high and low VEGF levels are associated with increased CVD risk [74].

Combining high and low dose COC into one group removed the IL‐16 difference, suggesting a possible dose–response relationship; however, no differences were found when comparing high and low dose COC groups. No consistent differences emerged between COC generations. Overall, COC use was associated with modest but consistent reductions in several immune‐activation markers, indicating an effect on immune cell recruitment and activation. Whether the effects increase or decrease immune activation may be both tissue and cell‐specific. Animal models or in vitro studies could help elucidate cell and tissue‐specific differences, as could further studies on more varied sample material to discern localized and systemic effects.

## 5 Strengths and Limitations

Prescription data may not perfectly reflect actual use, and likewise, it was unknown when LNG‐IUDs were removed or inserted after the prescriptions were filled. Donors are not allowed to donate when pregnant and are advised to avoid donation while attempting to become pregnant, hopefully reducing misclassification of IUD exposure. Excluding overlapping prescriptions reduced exposure uncertainty. The six‐month wash‐out period was applied to minimize residual HC effects. Restricting analysis to never‐users only reduced power and altered some of the associations otherwise observed. The NPR coverage from 1994 onwards may misclassify some of the older participants as never‐users. There is potentially some unrealized selection bias of never‐users, but as blood donors are generally very healthy, the effect this bias may have on inflammatory markers is likely to be limited. Adjusting for duration suggested that HC use itself, rather than duration, drives most associated biomarker differences (Table S8). Longitudinal samples would be better suited for studying the effects of duration of use. Several ATC codes contain mixed dosages, but G03AA11 only contains high doses and has several unique differences, and although there were only 52, it supports a dose‐dependent effect. Donation eligibility criteria reduce confounding from acute illness, such as infections, and associated inflammation. However, donors may still have a‐ or pre‐symptomatic infections influencing biomarker levels. Some donors will have experienced menopause either before or after the median age of 50 years, but only 58 HC users were older than 50 years. HC use is clearly limited in this age group, and the influence on the findings is likely limited. The LNG‐IUD and POP users were closer in age to the non‐users than the other groups. If there are differences in, e.g., socioeconomic status or alcohol consumption between the groups, this could influence estimates and obscure or augment differences between groups. The models would have benefited from adjustment for alcohol consumption, but this data was not available. BMI was available for adjustment, generally increases with age, and is associated with several lifestyle and socioeconomic factors [75, 76]. Information on the menstrual cycle was also unavailable, but HC, by design, influences the circulating hormone levels and affects the natural cycle. If there are certain times during the natural cycle when donors are more or less likely to donate, this could create a difference from the HC groups that may, for instance, skip a menstrual phase. Additionally, inflammation is well known to change across the menstrual cycle [77, 78]. Models were adjusted for age, current smoking, and BMI as these are known to influence the levels of several of the investigated biomarkers [17, 79, 80, 81]. Sample age, administrative region of sampling, and date of analysis were included to account for technical variation. Blood donors are generally healthier than the background population [82] and consequently, the observations reflect the differences in a healthy population, which may limit generalizability. Although the observed differences in white blood cell parameters were small, the high number of repeated measurements provides a powerful analysis in support of the observed biomarker differences.

In conclusion, we observed higher levels of markers of angiogenesis and growth factors and lower levels of sVCAM‐1 and the receptors Tie‐2 and Flt‐1 in COC users than non‐users. COC use was not associated with levels of proinflammatory markers apart from increased CRP and SAA and lower IL‐16 and IL‐17B. There were lower levels in several chemokines, indicating an association between COC and lower immune cell motility and activity. The effects of IUD were limited to lower TSLP and IL‐15. No associations with oral progestin‐only use were observed. COC use was associated with a higher number of circulating lymphocytes and lower basophil, monocyte, and eosinophil numbers. This study emphasizes the widespread impact of hormonal contraception on the immune and vascular systems and how the impact may differ between HC types.

## Funding

The Danish Blood Donor Study is funded by a Novo Nordisk Research Infrastructure grant (NNF23OC0082015), the Danish Administrative Regions, and the Danish Blood Donor Research Foundation. The infrastructure of the Danish Bio‐ and Genome Bank facilitated sample collections. BK was supported by a grant from BERTHA—the Danish Big Data Centre for Environment and Health, which, in turn, is funded by the Novo Nordisk Foundation Challenge Programme (grant NNF17OC0027864).

## Ethics Statement

Oral and written informed consent was obtained from all study participants. The DBDS was approved by the Danish Data Protection Agency (P‐2019‐99) and the Committees on Health Research Ethics in the Central Denmark Region (1–10–72‐95‐13) and the Zealand Region (SJ‐740). The authors confirm that the ethical policies of the journal, as noted on the journal's author guidelines page, have been adhered to, and the appropriate ethical review committee approval has been received.

## Conflicts of Interest

The authors declare no conflicts of interest.

## Supporting information


**Figure S1:** Fold change estimates in proinflammatory biomarkers in combined oral contraceptive users compared to the non‐users. Estimates are given with 95% confidence intervals in brackets. The CRP and SAA estimates extend somewhat beyond the axis limits and have been cut at 2.

**Figure S2:** Fold change estimates in T cell‐derived biomarkers in combined oral contraceptive users compared to the non‐users. Estimates are given with 95% confidence intervals in brackets.

**Figure S3:** Fold change estimates in chemokine biomarkers in combined oral contraceptive users compared to the non‐users. Estimates are given with 95% confidence intervals in brackets.

**Figure S4:** Fold change estimates in growth factor biomarkers in combined oral contraceptive users compared to the non‐users. Estimates are given with 95% confidence intervals in brackets.

**Table S1:** The number of observations for the different differential cell counts and the distribution of contraceptive users among those with a white blood cell count available.

**Table S2:** Main model. Estimates are shown as percentage change, and *p*‐values are corrected for multiple testing. The model is adjusted for age, current smoking, BMI, sample age, sample analysis date, and the Danish administrative region of sampling.
**Table S3:** Reduced model adjusted for age, current smoking, BMI, and sample age. Estimates are shown as percentage change, and *p*‐values are corrected for multiple testing.
**Table S4:** Unadjusted model. Estimates are shown as percentage change, and *p*‐values are corrected for multiple testing.
**Table S5:** Model combining COC low and COC high into a group with all COC users. Estimates are shown as percentage change, and *p*‐values are corrected for multiple testing. The model is adjusted for age, current smoking, BMI, sample age, sample analysis date, and the Danish administrative region of sampling.
**Table S6:** Model comparing the COC high group to the COC low group. Estimates are shown as percentage change, and *p*‐values are shown both as crude and as corrected for multiple testing. The model is adjusted for age, current smoking, BMI, sample age, sample analysis date, and the Danish administrative region of sampling.
**Table S7:** Model including duration of use as a covariate. Estimates are shown as percentage change, and *p*‐values are corrected for multiple testing. The model is adjusted for age, current smoking, BMI, duration of use, sample age, sample analysis date, and the Danish administrative region of sampling.
**Table S8:** Model using duration of use as the exposure. Estimates are shown as percentage change for each month of consecutive contraceptive use, and *p*‐values are corrected for multiple testing. The model is adjusted for age, current smoking, BMI, sample age, sample analysis date, and the Danish administrative region of sampling.
**Table S9:** Model excluding those who have been on hormonal contraceptives for less than 28 days. Estimates are shown as percentage change, and *p*‐values are corrected for multiple testing. The model is adjusted for age, current smoking, BMI, sample age, sample analysis date, and the Danish administrative region of sampling.
**Table S10:** Model that only allows those who have never used hormonal contraceptives in the non‐user group and in the menopausal group. Estimates are shown as percentage change, and *p*‐values are corrected for multiple testing. The model is adjusted for age, current smoking, BMI, sample age, sample analysis date, and the Danish administrative region of sampling.
**Table S11:** Model similar to the one in Table S10, but allowing for one previous prescription while still being counted as a never‐user. Estimates are shown as percentage change, and *p*‐values are corrected for multiple testing. The model is adjusted for age, current smoking, BMI, sample age, sample analysis date, and the Danish administrative region of sampling.
**Table S12:** Model with ATC codes as exposure instead of group. Estimates are shown as percentage change, and *p*‐values are corrected for multiple testing. The model is adjusted for age, current smoking, BMI, sample age, sample analysis date, and the Danish administrative region of sampling. ATC codes with fewer than 50 users are excluded. In the top row, the n for each ATC code is noted.
**Table S13:** Model with hormone therapy users compared to the menopausal group. Estimates are shown as percentage change, and *p*‐values are corrected for multiple testing. The model is adjusted for age, current smoking, BMI, sample age, sample analysis date, and the Danish administrative region of sampling.

Supinfo1:

## Data Availability

The DBDS is a platform for studies carried out by the Danish blood centres and collaborators. The study is managed by a steering committee who respond to enquiries regarding collaboration. The blood donors participate in the DBDS to increase the scope of their donation, i.e., to help produce valuable research for the benefit of future patients. Additional information can be found at our home page [http://www.dbds.dk]. Individual level data used for this study are not publicly available, as the data are restricted to approved research projects based in Denmark given the Danish GDPR legislation. External researchers may be granted access through collaborations upon contacting the DBDS steering committee (info@dbds.dk), following approval from The Regional Committees on Health Research Ethics and the Danish Data Protection Agency [http://www.datatilsynet.dk].

## References

[1] S. W. Junod , “Women's Trials: The Approval of the First Oral Contraceptive Pill in the United States and Great Britain,” Journal of the History of Medicine and Allied Sciences 57, no. 2 (2002): 117–160, 10.1093/jhmas/57.2.117. 11995593

[2] S. I. P. Kristensen and Ø. Lidegaard , “Hormonal Contraceptive Use in Denmark 2010‐2019,” Danish Medical Journal 68, no. 6 (2021): A08200599. 34060463

[3] K. Daniels and J. C. Abma , “Contraceptive Methods Women Have Ever Used:United States, 2015–2019,” National Health Statistics Reports 195 (2023): 1–18. PubMed PMID: 38170816. 38170816

[4] A. Hellström , K. Gemzell Danielsson , and H. Kopp Kallner , “Trends in Use and Attitudes Towards Contraception in Sweden: Results of a Nationwide Survey,” European Journal of Contraception & Reproductive Health Care 24, no. 2 (2019): 154–160, 10.1080/13625187.2019.1581163. 30920325

[5] C. J. Sørensen , O. B. Pedersen , M. S. Petersen , et al., “Combined Oral Contraception and Obesity Are Strong Predictors of Low‐Grade Inflammation in Healthy Individuals: Results From the Danish Blood Donor Study (DBDS),” PLoS One 9, no. 2 (2014): e88196, 10.1371/journal.pone.0088196. 24516611 PMC3916399

[6] D. Zhang , M. Sun , D. Samols , and I. Kushner , “STAT3 Participates in Transcriptional Activation of the C‐Reactive Protein Gene by Interleukin‐6,” Journal of Biological Chemistry 271, no. 16 (1996): 9503–9509, 10.1074/jbc.271.16.9503. 8621622

[7] P. Calabró , J. T. Willerson , and E. T. H. Yeh , “Inflammatory Cytokines Stimulated C‐Reactive Protein Production by Human Coronary Artery Smooth Muscle Cells,” Circulation 108, no. 16 (2003): 1930–1932, 10.1161/01.CIR.0000096055.62724.C5. 14530191

[8] M. B. Pepys and G. M. Hirschfield , “C‐Reactive protein: a critical update,” Journal of Clinical Investigation 111, no. 12 (2003): 1805–1812, 10.1172/JCI200318921. 12813013 PMC161431

[9] N. Rifai and P. M. Ridker , “Population Distributions of C‐Reactive Protein in Apparently Healthy Men and Women in the United States: Implication for Clinical Interpretation,” Clinical Chemistry 49, no. 4 (2003): 666–669, 10.1373/49.4.666. PubMed PMID: 12651826. 12651826

[10] A. A. Divani , X. Luo , Y. H. Datta , J. D. Flaherty , and A. Panoskaltsis‐Mortari , “Effect of Oral and Vaginal Hormonal Contraceptives on Inflammatory Blood Biomarkers,” Mediators of Inflammation 2015 (2015): 379501, 10.1155/2015/379501. 25861161 PMC4378601

[11] B. Larsen , A. Cox , C. Colbey , et al., “Inflammation and Oral Contraceptive Use in Female Athletes Before the Rio Olympic Games,” Frontiers in Physiology 11 (2020): 497, 10.3389/fphys.2020.00497. 32523546 PMC7261912

[12] W. V. Williams , “Hormonal Contraception and the Development of Autoimmunity: A Review of the Literature,” Linacre Quarterly 84, no. 3 (2017): 275–295, 10.1080/00243639.2017.1360065. 28912620 PMC5592309

[13] N. Dordevic , C. Dierks , E. Hantikainen , et al., “Pervasive Influence of Hormonal Contraceptives on the Human Plasma Proteome in a Broad Population Study,” medRxiv (2023), 10.1101/2023.10.11.23296871.

[14] L. DeRoo , M. Abbas , G. Goodney , and A. Gaye , “Changes in Proteome Profiles Linked to Hormonal Contraceptive Use Among African American Women With Untreated High Blood Pressure,” bioRxiv (2024), 10.1101/2024.08.12.607634.

[15] M. H. Kangasniemi , R. K. Arffman , S. Joenväärä , et al., “Ethinylestradiol in Combined Hormonal Contraceptive Has a Broader Effect on Serum Proteome Compared With Estradiol Valerate: A Randomized Controlled Trial,” Human Reproduction 38, no. 1 (2023): 89–102, 10.1093/humrep/deac250. 36416543 PMC9825269

[16] C. Erikstrup , E. Sørensen , K. R. Nielsen , et al., “Cohort Profile: The Danish Blood Donor Study,” International Journal of Epidemiology 52, no. 3 (2023): e162–e171, 10.1093/ije/dyac194. 36194120 PMC10244044

[17] B. Kjerulff , J. Dowsett , R. L. Jacobsen , et al., “Lifestyle and Demographic Associations With 47 Inflammatory and Vascular Stress Biomarkers in 9876 Blood Donors,” Community Medicine 4, no. 1 (2024): 50, 10.1038/s43856-024-00474-2. PMC10944541 38493237

[18] H. W. Kildemoes , H. T. Sørensen , and J. Hallas , “The Danish National Prescription Registry,” Scandinavian Journal of Public Health 39, no. 7 Suppl (2011): 38–41, 10.1177/1403494810394717. PubMed PMID: 21775349. 21775349

[19] A. Pottegård , S. A. J. Schmidt , H. Wallach‐Kildemoes , H. T. Sørensen , J. Hallas , and M. Schmidt , “Data Resource Profile: The Danish National Prescription Registry,” International Journal of Epidemiology 46, no. 3 (2017): 798–798f, 10.1093/ije/dyw213. PubMed PMID: 27789670; PubMed Central PMCID: PMC5837522. 27789670 PMC5837522

[20] S. J. Phillips , L. G. Hofler , A. M. Modest , L. F. B. Harvey , L. H. Wu , and M. R. Hacker , “Continuation of Copper and Levonorgestrel Intrauterine Devices: A Retrospective Cohort Study,” American Journal of Obstetrics and Gynecology 217, no. 1 (2017): 57.e1–57.e6, 10.1016/j.ajog.2017.03.005. PMC5810132 28315664

[21] J. T. Diedrich , T. Madden , Q. Zhao , and J. F. Peipert , “Long‐Term Utilization and Continuation of Intrauterine Devices,” American Journal of Obstetrics and Gynecology 213, no. 6 (2015): 822.e1–822.e6, 10.1016/j.ajog.2015.08.077. PMC4679676 26409157

[22] K. Rostgaard , “Simple Nested Bayesian Hypothesis Testing for Meta‐Analysis, Cox, Poisson and Logistic Regression Models,” Scientific Reports 13, no. 1 (2023): 4731, 10.1038/s41598-023-31838-8. 36959371 PMC10036629

[23] A. Husby , A. Laksafoss , E. M. Thiesson , K. D. Jakobsen , M. Andersson , and K. Rostgaard , “EpiForsk: Code Sharing at the Department of Epidemiological Research at Statens Serum Institut,” R package version (2023), 10.32614/CRAN.package.EpiForsk.

[24] H. Maia , J. Casoy , K. Pimentel , et al., “Effect of Oral Contraceptives on Vascular Endothelial Growth Factor, Cox‐2 and Aromatase Expression in the Endometrium of Uteri Affected by Myomas and Associated Pathologies,” Contraception 78, no. 6 (2008): 479–485, 10.1016/j.contraception.2008.07.002. 19014794

[25] S. Jesmin , C. N. Mowa , S. N. Sultana , et al., “VEGF Signaling Is Disrupted in the Hearts of Mice Lacking Estrogen Receptor Alpha,” European Journal of Pharmacology 641, no. 2–3 (2010): 168–178, 10.1016/j.ejphar.2010.05.020. 20639141

[26] Q. Meng , Y. Li , T. Ji , et al., “Estrogen Prevent Atherosclerosis by Attenuating Endothelial Cell Pyroptosis via Activation of Estrogen Receptor α‐Mediated Autophagy,” Journal of Advanced Research 28 (2021): 149–164, 10.1016/j.jare.2020.08.010. 33364052 PMC7753237

[27] K. Kavanagh , M. A. Davis , L. Zhang , et al., “Estrogen Decreases Atherosclerosis in Part by Reducing Hepatic Acyl‐CoA:Cholesterol Acyltransferase 2 (ACAT2) in Monkeys,” Arteriosclerosis, Thrombosis, and Vascular Biology 29, no. 10 (2009): 1471–1477, 10.1161/ATVBAHA.109.191825. 19759374 PMC2763273

[28] C. Borrás , M. Ferrando , M. Inglés , et al., “Estrogen Replacement Therapy Induces Antioxidant and Longevity‐Related Genes in Women After Medically Induced Menopause,” Oxidative Medicine and Cellular Longevity 2021 (2021): 1–9, 10.1155/2021/8101615. PMC8448598 34539974

[29] W. Rui , L. Guan , F. Zhang , W. Zhang , and W. Ding , “PM _2.5_ ‐Induced Oxidative Stress Increases Adhesion Molecules Expression in Human Endothelial Cells Through the ERK/AKT/NF‐κB‐Dependent Pathway: PM2.5 Effect Adhesion Molecule Expression via the ERK/AKT/NF‐κB Pathway,” Journal of Applied Toxicology 36, no. 1 (2016): 48–59, 10.1002/jat.3143. 25876056

[30] D. E. Morales , M. G. KA , D. S. Grant , et al., “Estrogen Promotes Angiogenic Activity in Human Umbilical Vein Endothelial Cells in Vitro and in a Murine Model,” Circulation 91, no. 3 (1995): 755–763, 10.1161/01.CIR.91.3.755. 7530174

[31] K. Krasinski , I. Spyridopoulos , T. Asahara , R. van der Zee , J. M. Isner , and D. W. Losordo , “Estradiol Accelerates Functional Endothelial Recovery After Arterial Injury,” Circulation 95, no. 7 (1997): 1768–1772, 10.1161/01.CIR.95.7.1768. 9107161

[32] I. Souter , C. Janzen , O. Martinez‐Maza , et al., “Serum Levels of Soluble Vascular Cell Adhesion Molecule‐1 Are Decreased in Women Receiving Oral Contraceptives Compared With Normally Menstruating Women: Implications in Atherosclerosis,” Fertility and Sterility 83, no. 5 (2005): 1480–1488, 10.1016/j.fertnstert.2004.11.048. 15866588

[33] C. N. Bairey Merz , B. D. Johnson , S. Berga , G. Braunstein , S. E. Reis , and V. Bittner , “Past Oral Contraceptive Use and Angiographic Coronary Artery Disease in Postmenopausal Women: Data From the National Heart, Lung, and Blood Institute–Sponsored Women's Ischemia Syndrome Evaluation,” Fertility and Sterility 85, no. 5 (2006): 1425–1431, 10.1016/j.fertnstert.2006.01.009. 16600235

[34] J. S. Williams and M. J. MacDonald , “Influence of Hormonal Contraceptives on Peripheral Vascular Function and Structure in Premenopausal Females: A Review,” American Journal of Physiology. Heart and Circulatory Physiology 320, no. 1 (2021): H77–H89, 10.1152/ajpheart.00614.2020. 33164574

[35] D. Wolf and K. Ley , “Immunity and Inflammation in Atherosclerosis,” Circulation Research 124, no. 2 (2019): 315–327, 10.1161/CIRCRESAHA.118.313591. 30653442 PMC6342482

[36] J. R. Zolton , L. A. Sjaarda , S. L. Mumford , et al., “Circulating Vascular Endothelial Growth Factor and Soluble Fms‐Like Tyrosine Kinase‐1 as Biomarkers for Endometrial Remodeling Across the Menstrual Cycle,” Obstetrics & Gynecology 137, no. 1 (2021): 82–90, 10.1097/AOG.0000000000004171. 33278289 PMC7746598

[37] Y. H. Kusumanto , G. A. P. Hospers , W. J. Sluiter , W. A. Dam , C. Meijer , and N. H. Mulder , “Circulating Vascular Endothelial Growth Factor During the Normal Menstrual Cycle,” Anticancer Research 24, no. 6 (2004): 4237–4241. PubMed PMID: 15736478. 15736478

[38] A. Malamitsi‐Puchner , A. Sarandakou , J. Tziotis , A. Stavreus‐Evers , A. Tzonou , and B. M. Landgren , “Circulating Angiogenic Factors During Periovulation and the Luteal Phase of Normal Menstrual Cycles,” Fertility and Sterility 81, no. 5 (2004): 1322–1327, 10.1016/j.fertnstert.2003.10.025. 15136097

[39] C. Hong , M. A. Luckey , and J. H. Park , “Intrathymic IL‐7: The Where, When, and Why of IL‐7 Signaling During T Cell Development,” Seminars in Immunology 24, no. 3 (2012): 151–158, 10.1016/j.smim.2012.02.002. 22421571 PMC3358706

[40] A. L. Zoller and G. J. Kersh , “Estrogen Induces Thymic Atrophy by Eliminating Early Thymic Progenitors and Inhibiting Proliferation of β‐Selected Thymocytes,” Journal of Immunology 176, no. 12 (2006): 7371–7378, 10.4049/jimmunol.176.12.7371. 16751381

[41] M. D. Taves and J. D. Ashwell , “Effects of Sex Steroids on Thymic Epithelium and Thymocyte Development,” Frontiers in Immunology 13 (2022): 975858, 10.3389/fimmu.2022.975858. 36119041 PMC9478935

[42] M. Nakaya , H. Tachibana , and K. Yamada , “Effect of Estrogens on the Interferon‐γ Producing Cell Population of Mouse Splenocytes,” Bioscience, Biotechnology, and Biochemistry 70, no. 1 (2006): 47–53, 10.1271/bbb.70.47. 16428820

[43] H. S. Fox , B. L. Bond , and T. G. Parslow , “Estrogen Regulates the IFN‐Gamma Promoter,” Journal of Immunology 146, no. 12 (1991): 4362–4367. PubMed PMID: 1904081. 1904081

[44] R. P. Singh , B. H. Hahn , and D. S. Bischoff , “Interferon Genes Are Influenced by 17β‐Estradiol in SLE,” Frontiers in Immunology 12 (2021): 725325, 10.3389/fimmu.2021.725325. 34733276 PMC8558410

[45] C. E. Repeke , T. P. Garlet , C. F. Francisconi , D. Broll , A. P. F. Trombone , and G. P. Garlet , Encyclopedia of Signaling Molecules [Internet], Springer Cham, ed. S. Choi (2016), 10.1007/978-3-319-67199-4.

[46] M. Mariani , R. Lang , E. Binda , P. Panina‐Bordignon , and D. D'Ambrosio , “Dominance of CCL22 Over CCL17 in Induction of Chemokine Receptor CCR4 Desensitization and Internalization on Human Th2 Cells,” European Journal of Immunology 34, no. 1 (2004): 231–240, 10.1002/eji.200324429. 14971049

[47] T. Imai , D. Chantry , C. J. Raport , et al., “Macrophage‐Derived Chemokine Is a Functional Ligand for the CC Chemokine Receptor 4,” Journal of Biological Chemistry 273, no. 3 (1998): 1764–1768, 10.1074/jbc.273.3.1764. 9430724

[48] B. I. Nwaru , R. Pillinger , H. Tibble , et al., “Hormonal Contraceptives and Onset of Asthma in Reproductive‐Age Women: Population‐Based Cohort Study,” Journal of Allergy and Clinical Immunology 146, no. 2 (2020): 438–446, 10.1016/j.jaci.2020.02.027. PubMed PMID: 32305347. 32305347

[49] C. Combadiere , S. K. Ahuja , and P. M. Murphy , “Cloning and Functional Expression of a Human Eosinophil CC Chemokine Receptor,” Journal of Biological Chemistry 270, no. 28 (1995): 16491–16494, 10.1074/jbc.270.28.16491. PubMed PMID: 7622448. 7622448

[50] M. Uguccioni , C. R. Mackay , B. Ochensberger , et al., “High Expression of the Chemokine Receptor CCR3 in Human Blood Basophils. Role in Activation by Eotaxin, MCP‐4, and Other Chemokines,” Journal of Clinical Investigation 100, no. 5 (1997): 1137–1143, 10.1172/JCI119624. PubMed PMID: 9276730; PubMed Central PMCID: PMC508288. 9276730 PMC508288

[51] A. Rubbert , C. Combadiere , M. Ostrowski , et al., “Dendritic Cells Express Multiple Chemokine Receptors Used as Coreceptors for HIV Entry,” Journal of Immunology 160, no. 8 (1998): 3933–3941. PubMed PMID: 9558100. 9558100

[52] F. Sallusto , C. R. Mackay , and A. Lanzavecchia , “Selective Expression of the Eotaxin Receptor CCR3 by Human T Helper 2 Cells,” Science 277, no. 5334 (1997): 2005–2007, 10.1126/science.277.5334.2005. PubMed PMID: 9302298. 9302298

[53] G. Huang , L. Tao , S. Shen , and L. Chen , “Hypoxia Induced CCL28 Promotes Angiogenesis in Lung Adenocarcinoma by Targeting CCR3 on Endothelial Cells,” Scientific Reports 6, no. 1 (2016): 27152, 10.1038/srep27152. 27250766 PMC4890017

[54] L. Auerbach , T. Hafner , J. C. Huber , and S. Panzer , “Influence of Low‐Dose Oral Contraception on Peripheral Blood Lymphocyte Subsets at Particular Phases of the Hormonal Cycle,” Fertility and Sterility 78, no. 1 (2002): 83–89, 10.1016/S0015-0282(02)03173-4. 12095495

[55] J. Nowak , B. Borkowska , and B. Pawlowski , “Leukocyte Changes Across Menstruation, Ovulation, and Mid‐Luteal Phase and Association With Sex Hormone Variation,” American Journal of Human Biology 28, no. 5 (2016): 721–728, 10.1002/ajhb.22856. 27088641

[56] A. J. Lengi , R. A. Phillips , E. Karpuzoglu , and S. A. Ahmed , “Estrogen Selectively Regulates Chemokines in Murine Splenocytes,” Journal of Leukocyte Biology 81, no. 4 (2007): 1065–1074, 10.1189/jlb.0606391. 17185357

[57] A. Yamaguchi , K. Nozawa , M. Fujishiro , et al., “Estrogen Inhibits Apoptosis and Promotes CC Motif Chemokine Ligand 13 Expression on Synovial Fibroblasts in Rheumatoid Arthritis,” Immunopharmacology and Immunotoxicology 34, no. 5 (2012): 852–857, 10.3109/08923973.2012.664149. 22393877

[58] R. C. Eldridge , N. Wentzensen , R. M. Pfeiffer , et al., “Endogenous Estradiol and Inflammation Biomarkers: Potential Interacting Mechanisms of Obesity‐Related Disease,” Cancer Causes & Control 31, no. 4 (2020): 309–320, 10.1007/s10552-020-01280-6. 32100190 PMC7472689

[59] J. W. Xu , J. Gong , X. M. Chang , et al., “Estrogen Reduces CCL4‐ Induced Liver Fibrosis in Rats,” World Journal of Gastroenterology 8, no. 5 (2002): 883–887, 10.3748/wjg.v8.i5.883. 12378635 PMC4656580

[60] K. G. Michel , R. P. H. Huijbregts , J. L. Gleason , H. E. Richter , and Z. Hel , “Effect of Hormonal Contraception on the Function of Plasmacytoid Dendritic Cells and Distribution of Immune Cell Populations in the Female Reproductive Tract,” JAIDS Journal of Acquired Immune Deficiency Syndromes 68, no. 5 (2015): 8–511, 10.1097/QAI.0000000000000531. PMC4874780 25763784

[61] R. El‐Mallah , A. A. Saab , and N. Nassar , “Serum Interleukin‐17 and Estradiol Levels in Postmenopausal Women in Relation to Osteoporosis,” Egyptian Rheumatology and Rehabilitation 48, no. 1 (2021): 35, 10.1186/s43166-021-00083-0.

[62] Y. S. Rosenthal , A. Rosenthal , H. Shalev Ram , S. Ram , G. Chodick , and G. Koren , “Association Between Oral Contraceptives and Serious Infections: A Population‐Based Cohort Study,” British Journal of Clinical Pharmacology 87, no. 11 (2021): 4241–4251, 10.1111/bcp.14840. 34018215

[63] K. K. Chang , L. B. Liu , H. Li , et al., “TSLP Induced by Estrogen Stimulates Secretion of MCP‐1 and IL‐8 and Growth of Human Endometrial Stromal Cells Through JNK and NF‐κB Signal Pathways,” International Journal of Clinical and Experimental Pathology 7, no. 5 (2014): 1889–1899. PubMed PMID: 24966899; PubMed Central PMCID: PMC4069968. 24966899 PMC4069968

[64] Y. Urata , Y. Osuga , G. Izumi , et al., “Interleukin‐1β Stimulates the Secretion of Thymic Stromal Lymphopoietin (TSLP) From Endometrioma Stromal Cells: Possible Involvement of TSLP in Endometriosis,” Human Reproduction 27, no. 10 (2012): 3028–3035, 10.1093/humrep/des291. PubMed PMID: 22888172. 22888172

[65] A. J. W. Hsueh , E. J. Peck , and J. H. Clark , “Progesterone Antagonism of the Oestrogen Receptor and Oestrogen‐Induced Uterine Growth,” Nature 254, no. 5498 (1975): 337–339, 10.1038/254337a0. 163981

[66] K. Kitaya , J. Yasuda , I. Yagi , Y. Tada , S. Fushiki , and H. Honjo , “IL‐15 Expression at Human Endometrium and Decidua,” Biology of Reproduction 63, no. 3 (2000): 683–687, 10.1095/biolreprod63.3.683. PubMed PMID: 10952908. 10952908

[67] H. Okada , T. Nakajima , M. Sanezumi , A. Ikuta , K. Yasuda , and H. Kanzaki , “Progesterone Enhances Interleukin‐15 Production in Human Endometrial Stromal Cells in Vitro,” Journal of Clinical Endocrinology and Metabolism 85, no. 12 (2000): 4765–4770, 10.1210/jcem.85.12.7023. PubMed PMID: 11134140. 11134140

[68] U. Shanmugasundaram , J. F. Hilton , J. W. Critchfield , et al., “Effects of the Levonorgestrel‐Releasing Intrauterine Device on the Immune Microenvironment of the Human Cervix and Endometrium,” American Journal of Reproductive Immunology 76, no. 2 (2016): 137–148, 10.1111/aji.12535. 27401588 PMC5316474

[69] L. Morin‐Papunen , H. Martikainen , M. C. MI , et al., “Comparison of Metabolic and Inflammatory Outcomes in Women Who Used Oral Contraceptives and the Levonorgestrel‐Releasing Intrauterine Device in a General Population,” American Journal of Obstetrics and Gynecology 199, no. 5 (2008): 529.e1–529.e10, 10.1016/j.ajog.2008.04.013. 18533124

[70] E. Toffol , O. Heikinheimo , P. Jousilahti , et al., “Metabolomics Profile of 5649 Users and Nonusers of Hormonal Intrauterine Devices in Finland,” American Journal of Obstetrics and Gynecology 227, no. 4 (2022): 603.e1–603.e29, 10.1016/j.ajog.2022.06.009. 35697093

[71] C. W. Skovlund , L. S. Mørch , L. V. Kessing , and Ø. Lidegaard , “Association of Hormonal Contraception With Depression,” JAMA Psychiatry 73, no. 11 (2016): 1154, 10.1001/jamapsychiatry.2016.2387. 27680324

[72] L. S. Mørch , C. W. Skovlund , P. C. Hannaford , L. Iversen , S. Fielding , and Ø. Lidegaard , “Contemporary Hormonal Contraception and the Risk of Breast Cancer,” New England Journal of Medicine 377, no. 23 (2017): 2228–2239, 10.1056/NEJMoa1700732. 29211679

[73] E. Løkkegaard , “Evidensbaseret tilgang til kontraception,” Ugeskrift for Laeger 183 (2021): V205084. 34498582

[74] B. M. Kaess , S. R. Preis , A. Beiser , et al., “Circulating Vascular Endothelial Growth Factor and the Risk of Cardiovascular Events,” Heart 102, no. 23 (2016): 1898–1901, 10.1136/heartjnl-2015-309155. PubMed PMID: 27354275. 27354275

[75] M. V. Groth , S. Fagt , A. Stockmarr , J. Matthiessen , and A. Biltoft‐Jensen , “Dimensions of Socioeconomic Position Related to Body Mass Index and Obesity Among Danish Women and Men,” Scandinavian Journal of Public Health 37, no. 4 (2009): 418–426, 10.1177/1403494809105284. 19470691

[76] R. B. Hasselbalch , M. K. Andrea , C. V. Nolsøe , et al., “Association Between Socioeconomic Factors and Semaglutide Use for Weight Loss: A Population‐Based Cross‐Sectional Study in Denmark,” Lancet Regional Health ‐ Europe 56 (2025): 101398, 10.1016/j.lanepe.2025.101398. 41624087 PMC12859550

[77] J. Evans and L. A. Salamonsen , “Inflammation, Leukocytes and Menstruation,” Reviews in Endocrine & Metabolic Disorders 13, no. 4 (2012): 277–288, 10.1007/s11154-012-9223-7. PubMed PMID: 22865231. 22865231

[78] A. Azlan , L. A. Salamonsen , J. Hutchison , and J. Evans , “Endometrial Inflammasome Activation Accompanies Menstruation and May Have Implications for Systemic Inflammatory Events of the Menstrual Cycle,” Human Reproduction 35, no. 6 (2020): 1363–1376, 10.1093/humrep/deaa065. PubMed PMID: 32488243. 32488243

[79] M. F. Gallo , L. M. Lopez , D. A. Grimes , F. Carayon , K. F. Schulz , and F. M. Helmerhorst , “Combination Contraceptives: Effects on Weight,” Cochrane Database of Systematic Reviews 2014, no. 1 (2014): CD003987, 10.1002/14651858.CD003987.pub5. 24477630 PMC10640873

[80] L. M. Lopez , S. Ramesh , M. Chen , et al., “Progestin‐Only Contraceptives: Effects on Weight,” Cochrane Database of Systematic Reviews 2016, no. 8 (2016): CD008815, 10.1002/14651858.CD008815.pub4. PMC5034734 27567593

[81] E. R. Mayeda , A. H. Torgal , and C. L. Westhoff , “Weight and Body Composition Changes During Oral Contraceptive Use in Obese and Normal Weight Women,” Journal of Women's Health 23, no. 1 (2014): 38–43, 10.1089/jwh.2012.4241. PMC3880912 24156617

[82] T. Brodersen , K. Rostgaard , C. J. Lau , et al., “The Healthy Donor Effect and Survey Participation, Becoming a Donor and Donor Career,” Transfusion. 2023; 63(1): 143–155: trf.17190, 10.1111/trf.17190. PMC10107247 36479702

