# Supplementary material for: Effect of Hormonal Contraceptives on Circulating Biomarkers of Inflammation, Chemotaxis, Angiogenesis, and Vascular Stress
Source: APMIS. 2026 Jul 14;134(7):e70235. doi: 10.1111/apm.70235 (PMC13368256; doi:10.1111/apm.70235)
Supplement: Supplementary file 5 — Table S1: The number of observations for the different differential cell counts and the distribution of contraceptive users among those with a white blood cell count available. [file APM-134-0-s006.docx]

|  | | **WBC** | **NEUT** | | **LYMPH** | | **BASO** | | **MONO** | | **EO** |
| --- | --- | --- | --- | --- | --- | --- | --- | --- | --- | --- | --- |
| **Observations** | | 115248 | 75562 | | 75608 | | 73871 | | 74053 | | 73858 |
|  | **Non-user** | | | **High dose** | | **Low dose** | | **IUD** | | **POP** | |
| **Observations**  **(WBC)** | 51700 | | | 29297 | | 7649 | | 22630 | | 3973 | |

Supplementary Table 1
