# Supplementary material for: Effect of Hormonal Contraceptives on Circulating Biomarkers of Inflammation, Chemotaxis, Angiogenesis, and Vascular Stress
Source: APMIS. 2026 Jul 14;134(7):e70235. doi: 10.1111/apm.70235 (PMC13368256; doi:10.1111/apm.70235)
Supplement: Supplementary file 6 — Table S2: Main model. Estimates are shown as percentage change, and p‐values are corrected for multiple testing. The model is adjusted for age, current smoking, BMI, sample age, sample analysis date, and the Danish administrative region of sampling. Table S3: Reduced model adjusted for age, current smoking, BMI, and sample age. Estimates are shown as percentage change, and p‐values are corrected for multiple testing. Table S4: Unadjusted model. Estimates are shown as percentage change, and p‐values are corrected for multiple testing. Table S5: Model combining COC low and COC high into a group with all COC users. Estimates are shown as percentage change, and p‐values are corrected for multiple testing. The model is adjusted for age, current smoking, BMI, sample age, sample analysis date, and the Danish administrative region of sampling. Table S6: Model comparing the COC high group to the COC low group. Estimates are shown as percentage change, and p‐values are shown both as crude and as corrected for multiple testing. The model is adjusted for age, current smoking, BMI, sample age, sample analysis date, and the Danish administrative region of sampling. Table S7: Model including duration of use as a covariate. Estimates are shown as percentage change, and p‐values are corrected for multiple testing. The model is adjusted for age, current smoking, BMI, duration of use, sample age, sample analysis date, and the Danish administrative region of sampling. Table S8: Model using duration of use as the exposure. Estimates are shown as percentage change for each month of consecutive contraceptive use, and p‐values are corrected for multiple testing. The model is adjusted for age, current smoking, BMI, sample age, sample analysis date, and the Danish administrative region of sampling. Table S9: Model excluding those who have been on hormonal contraceptives for less than 28 days. Estimates are shown as percentage change, and p‐values are corrected for multiple testing. The model [file APM-134-0-s001.docx]

| **Supplementary Table 2** | |  |  |  |  |  |  |  |  |  |  |  |  |
| --- | --- | --- | --- | --- | --- | --- | --- | --- | --- | --- | --- | --- | --- |
| S1 | COC low |  | COC high |  | IUD |  | POP |  | HT |  | Menopausal |  |  |
| Assay | % estimate | corrected P | % estimate | corrected P | % estimate | corrected P | % estimate | corrected P | % estimate | corrected P | % estimate | corrected P | group |
| CRP | 292.4[237.3 : 356.6] | 1,78E-66 | 325.8[281.3 : 375.5] | 3,06E-134 | 5.2[-5.8 : 17.6] | 4,423800729 | -3.8[-23.4 : 20.9] | 8,909959457 | 32.6[1.8 : 72.7] | 0,436048038 | 16.7[3.5 : 31.5] | 0,137280562 | Proinflammatory |
| SAA | 60.4[41.4 : 82.0] | 3,15E-12 | 41.7[29.3 : 55.4] | 1,59E-12 | -10.6[-18.5 : -1.9] | 0,216021988 | -9.5[-25.2 : 9.4] | 3,624931763 | 36.4[9.5 : 70.1] | 0,068676103 | 19.9[8.6 : 32.5] | 0,004257169 | Proinflammatory |
| IL-1A | -4.5[-14.4 : 6.7] | 5,007516006 | -5.9[-13.2 : 2.1] | 1,728461443 | 0.7[-7.3 : 9.4] | 10,42653646 | -1.3[-16.9 : 17.1] | 10,5208712 | 2.1[-16.2 : 24.4] | 10,06931585 | 4.5[-4.4 : 14.1] | 3,990022866 | Proinflammatory |
| IL-1B | 5.2[-13.6 : 28.1] | 7,384810541 | -0.9[-14.3 : 14.7] | 10,89321197 | 0.8[-13.0 : 16.9] | 10,95717893 | -12.4[-35.5 : 19.1] | 4,788816168 | -1.0[-30.5 : 41.0] | 11,47497055 | 15.8[-1.2 : 35.6] | 0,838146958 | Proinflammatory |
| IL-1RA | 1.6[-5.4 : 9.2] | 7,886913805 | -4.0[-8.9 : 1.2] | 1,573584221 | 5.5[0.0 : 11.4] | 0,599041449 | -6.0[-15.9 : 5.1] | 3,322211266 | -4.7[-16.3 : 8.6] | 5,638360122 | 3.5[-2.4 : 9.6] | 3,00120876 | Proinflammatory |
| IL-3 | -1.7[-15.8 : 14.7] | 9,884268786 | 0.0[-10.8 : 12.1] | 11,93555969 | 11.6[-0.7 : 25.3] | 0,784397466 | 0.6[-21.1 : 28.2] | 11,53620444 | -27.6[-45.4 : -4.0] | 0,297430146 | 9.2[-3.6 : 23.7] | 1,992472621 | Proinflammatory |
| IL-6 | -2.3[-13.1 : 9.8] | 8,314890317 | -4.1[-12.0 : 4.5] | 4,075423931 | -5.8[-13.7 : 2.8] | 2,123083386 | 0.9[-15.9 : 20.9] | 11,10545805 | 9.2[-11.4 : 34.6] | 4,920202954 | 23.6[12.6 : 35.8] | 1,12E-04 | Proinflammatory |
| IL-12 | 4.7[-2.1 : 11.9] | 2,19233651 | 0.9[-3.9 : 6.0] | 8,483254019 | -0.4[-5.3 : 4.7] | 10,46004418 | 11.0[0.0 : 23.2] | 0,595292391 | 12.0[-0.7 : 26.3] | 0,773105404 | 9.1[3.4 : 15.1] | 0,018560982 | Proinflammatory |
| IL-12p70 | -5.7[-20.8 : 12.2] | 6,09166251 | -9.0[-20.0 : 3.5] | 1,803196734 | 6.6[-6.4 : 21.5] | 4,037042386 | 3.1[-21.4 : 35.3] | 9,906928099 | 18.3[-13.5 : 61.8] | 3,513848568 | 9.1[-5.2 : 25.5] | 2,684255412 | Proinflammatory |
| IL-15 | 0.6[-2.7 : 4.1] | 8,594729344 | 1.8[-0.7 : 4.4] | 1,873939749 | -4.0[-6.4 : -1.6] | 0,018587133 | -3.0[-7.9 : 2.3] | 3,181447534 | -0.1[-6.0 : 6.2] | 11,72382563 | 0.4[-2.3 : 3.1] | 9,535082625 | Proinflammatory |
| TNF-A | 0.1[-4.2 : 4.6] | 11,58390121 | -3.5[-6.5 : -0.3] | 0,364936849 | -2.2[-5.3 : 1.1] | 2,213021747 | 0.0[-6.6 : 7.1] | 11,94054185 | 14.9[6.2 : 24.3] | 0,006366485 | 10.6[6.8 : 14.6] | 2,09E-07 | Proinflammatory |
| TNF-B | -4.4[-13.7 : 5.9] | 4,648945275 | -8.0[-14.7 : -0.8] | 0,349718903 | -3.4[-10.5 : 4.2] | 4,423221925 | 5.1[-10.3 : 23.2] | 6,46166276 | 5.3[-12.3 : 26.5] | 6,932067288 | 8.5[-0.1 : 17.7] | 0,621313175 | Proinflammatory |
| IFN-G | 13.8[2.6 : 26.3] | 0,183913844 | 6.7[-1.2 : 15.2] | 1,252989993 | -2.8[-10.1 : 5.0] | 6,064034802 | 1.8[-13.4 : 19.6] | 10,77200571 | 6.3[-11.7 : 28.0] | 6,743043015 | 9.2[0.5 : 18.7] | 0,496034078 | T derived |
| IL-2 | 6.6[-10.9 : 27.7] | 6,309126813 | 4.9[-8.2 : 19.7] | 6,285651666 | 20.1[4.9 : 37.4] | 0,102917068 | -4.0[-27.5 : 27.1] | 10,09187558 | 73.5[25.6 : 139.6] | 0,010904509 | 13.0[-2.2 : 30.6] | 1,261645847 | T derived |
| IL-4 | 1.9[-13.8 : 20.5] | 10,68449359 | 3.9[-8.1 : 17.5] | 7,047454249 | 0.1[-11.7 : 13.5] | 12,85930085 | 10.4[-14.9 : 43.3] | 5,90772904 | 18.7[-12.1 : 60.3] | 3,415494907 | 7.3[-6.2 : 22.7] | 3,989881225 | T derived |
| IL-5 | 6.7[-2.6 : 17.0] | 2,126566709 | 4.3[-2.5 : 11.6] | 2,841429222 | 2.3[-4.5 : 9.5] | 6,81848864 | -4.9[-17.5 : 9.7] | 6,359198243 | 7.2[-9.1 : 26.3] | 5,320097588 | -0.8[-7.8 : 6.8] | 10,89764969 | T derived |
| IL-9 | -9.4[-23.8 : 7.8] | 3,472156392 | -8.0[-19.1 : 4.5] | 2,564694371 | 4.5[-8.2 : 19.0] | 6,559300816 | -9.0[-30.5 : 19.1] | 6,385327741 | 9.0[-20.4 : 49.2] | 7,705606599 | 20.1[4.5 : 38.1] | 0,130319614 | T derived |
| IL-10 | -8.1[-18.5 : 3.6] | 2,175680548 | -2.2[-10.4 : 6.9] | 8,189507816 | 0.5[-8.1 : 10.0] | 11,84884805 | 17.7[-2.4 : 41.9] | 1,148064813 | 12.9[-9.0 : 40.1] | 3,512402639 | 9.2[-0.8 : 20.3] | 0,953981986 | T derived |
| IL-13 | -10.1[-22.5 : 4.3] | 2,083742592 | 3.7[-7.1 : 15.6] | 6,727111795 | 6.1[-5.0 : 18.6] | 3,83794064 | 9.1[-13.4 : 37.5] | 5,953949444 | -8.2[-29.6 : 19.8] | 6,872820484 | -6.9[-17.4 : 4.9] | 3,112338244 | T derived |
| IL-16 | -2.8[-10.7 : 5.9] | 6,79380614 | -10.5[-16.0 : -4.7] | 0,007237892 | 4.7[-1.8 : 11.6] | 2,119829791 | -4.7[-16.6 : 8.9] | 6,217677149 | 2.8[-11.9 : 19.8] | 9,451768407 | 6.7[-0.4 : 14.3] | 0,862552615 | T derived |
| IL-17A | 5.8[-3.2 : 15.6] | 2,744580637 | 2.6[-3.9 : 9.6] | 5,667215357 | 0.6[-5.9 : 7.5] | 11,17579463 | 7.0[-6.8 : 22.8] | 4,409882996 | 23.0[4.9 : 44.2] | 0,141020751 | 10.7[3.1 : 18.9] | 0,065300922 | T derived |
| IL-17A/F | 5.3[-8.8 : 21.7] | 6,253516111 | 5.5[-5.1 : 17.4] | 4,179234575 | -3.3[-13.2 : 7.8] | 7,077272433 | -16.6[-33.3 : 4.4] | 1,470954912 | -9.5[-30.4 : 17.6] | 5,893242686 | -3.0[-13.6 : 9.0] | 7,922599885 | T derived |
| IL-17B | -18.1[-27.7 : -7.2] | 0,022313822 | -15.1[-22.6 : -6.9] | 0,006237937 | -9.6[-17.7 : -0.8] | 0,440149754 | -17.6[-32.2 : 0.1] | 0,667846185 | 14.6[-8.6 : 43.7] | 3,067409734 | 13.5[2.7 : 25.5] | 0,172656876 | T derived |
| IL-17C | 4.6[-9.0 : 20.2] | 6,894535729 | -8.3[-17.3 : 1.6] | 1,266846413 | -7.9[-17.0 : 2.3] | 1,607687371 | -12.6[-29.6 : 8.6] | 2,932892386 | 1.6[-21.2 : 30.9] | 11,74586315 | 1.4[-9.4 : 13.4] | 10,57785685 | T derived |
| IL-17D | 6.1[-6.3 : 20.2] | 4,578853514 | 0.7[-8.2 : 10.3] | 11,5567201 | -2.5[-11.2 : 7.0] | 7,702368203 | 3.0[-15.1 : 25.1] | 9,899363952 | -0.2[-20.4 : 25.2] | 12,84312202 | 17.3[6.1 : 29.7] | 0,02379668 | T derived |
| CCL2 | 7.1[0.6 : 14.0] | 0,311609771 | 1.6[-2.9 : 6.3] | 4,91229548 | -1.0[-5.3 : 3.6] | 6,713490117 | -0.1[-9.2 : 9.8] | 9,774087587 | 16.8[4.9 : 30.0] | 0,045014989 | 16.2[10.6 : 21.9] | 1,73E-08 | Chemokine |
| CCL3 | 1.0[-5.4 : 7.8] | 7,747236668 | 4.5[-0.3 : 9.6] | 0,677472361 | -1.0[-5.6 : 3.8] | 6,818683093 | 8.5[-1.8 : 19.9] | 1,090926253 | 10.3[-1.5 : 23.5] | 0,880671074 | 8.7[3.3 : 14.4] | 0,013458736 | Chemokine |
| CCL4 | -3.5[-10.1 : 3.7] | 3,334500062 | -7.7[-12.3 : -2.8] | 0,025303922 | -2.2[-7.2 : 3.0] | 3,9538607 | -4.7[-14.6 : 6.2] | 3,825096082 | 9.5[-3.2 : 23.8] | 1,497024467 | 8.7[2.8 : 14.9] | 0,033995 | Chemokine |
| CCL11 | -6.9[-12.1 : -1.4] | 0,14713929 | -10.9[-14.5 : -7.1] | 5,22E-07 | -3.0[-7.0 : 1.1] | 1,444954813 | 3.8[-4.8 : 13.3] | 3,986549256 | 0.2[-9.2 : 10.6] | 9,639177053 | 7.8[3.0 : 12.7] | 0,010458092 | Chemokine |
| CCL13 | -10.7[-17.0 : -4.0] | 0,023175838 | -12.8[-17.2 : -8.0] | 3,76E-06 | 0.5[-4.7 : 5.9] | 8,542164299 | -9.5[-19.0 : 1.2] | 0,790777036 | 5.0[-7.4 : 19.0] | 4,454661765 | 4.8[-1.0 : 10.9] | 1,063417492 | Chemokine |
| CCL17 | -21.6[-29.2 : -13.2] | 2,88E-05 | -19.8[-25.5 : -13.7] | 4,29E-08 | 0.8[-6.4 : 8.5] | 8,318991333 | -6.0[-19.5 : 9.8] | 4,349696431 | 13.2[-5.0 : 34.8] | 1,642658727 | 19.7[10.6 : 29.6] | 8,89E-05 | Chemokine |
| CCL22 | 5.9[0.9 : 11.1] | 0,205450503 | -0.3[-3.7 : 3.3] | 8,79239492 | -0.9[-4.3 : 2.6] | 5,951719729 | -2.1[-9.1 : 5.3] | 5,662139121 | 20.0[10.4 : 30.3] | 1,78E-04 | 16.2[11.9 : 20.7] | 7,19E-14 | Chemokine |
| CCL26 | -3.2[-12.7 : 7.3] | 5,310062163 | -7.5[-14.1 : -0.4] | 0,396194268 | -1.3[-8.4 : 6.3] | 7,278002274 | -4.3[-18.2 : 11.9] | 5,793908511 | 2.3[-14.3 : 22.0] | 8,04624055 | -3.6[-11.0 : 4.5] | 3,769009944 | Chemokine |
| IL-8 | 3.4[-6.9 : 15.0] | 5,30770602 | 6.8[-1.2 : 15.4] | 0,994050566 | 1.6[-6.1 : 10.0] | 6,875734545 | -9.5[-23.2 : 6.7] | 2,353562366 | 1.5[-16.0 : 22.7] | 8,759181248 | 5.9[-2.7 : 15.3] | 1,826598321 | Chemokine |
| CXCL10 | -2.9[-10.0 : 4.8] | 4,53049711 | -7.1[-12.0 : -1.8] | 0,088571748 | 0.8[-4.6 : 6.5] | 7,768263225 | 0.4[-10.6 : 12.8] | 9,419791928 | 10.1[-3.4 : 25.5] | 1,49382117 | 12.5[6.0 : 19.4] | 0,001050121 | Chemokine |
| bFGF | 7.4[-4.6 : 20.8] | 2,851660307 | 13.9[4.4 : 24.3] | 0,039682531 | -2.5[-10.7 : 6.5] | 6,945775022 | -11.8[-26.6 : 6.0] | 2,17942062 | 10.1[-10.9 : 36.0] | 4,494510812 | 7.1[-2.6 : 17.8] | 1,852570465 | Growth Factor |
| Flt-1 | -15.6[-19.3 : -11.9] | 4,37E-13 | -15.5[-18.1 : -12.7] | 6,61E-23 | -1.6[-4.8 : 1.7] | 4,071642434 | 3.1[-3.7 : 10.4] | 4,555129084 | -8.3[-15.2 : -0.8] | 0,375815077 | -0.4[-3.8 : 3.2] | 10,00939613 | Growth Factor |
| GM-CSF | -6.0[-17.8 : 7.5] | 4,408291003 | -6.2[-15.1 : 3.5] | 2,417180663 | -3.2[-12.5 : 7.0] | 6,266997329 | 2.1[-17.2 : 25.9] | 10,12747055 | 12.9[-11.3 : 43.7] | 3,880940803 | 10.0[-1.3 : 22.5] | 1,020656348 | Growth Factor |
| IL-7 | 9.3[1.9 : 17.3] | 0,162138075 | 12.4[6.7 : 18.4] | 1,36E-04 | -5.8[-10.6 : -0.6] | 0,333408991 | -5.0[-14.9 : 6.1] | 4,353869734 | -4.2[-15.6 : 8.8] | 6,140924198 | 0.0[-5.5 : 5.8] | 11,89967257 | Growth Factor |
| PlGF | -0.9[-4.4 : 2.6] | 7,239951384 | 2.3[-0.3 : 5.0] | 0,9969681 | -0.7[-3.3 : 2.0] | 7,357553802 | -2.0[-7.2 : 3.6] | 5,778669858 | 8.4[1.7 : 15.5] | 0,151686406 | 8.9[5.9 : 12.1] | 4,61E-08 | Growth Factor |
| sICAM-1 | 4.9[0.0 : 10.0] | 0,607424961 | 1.1[-2.4 : 4.6] | 6,603516635 | -1.7[-5.1 : 1.8] | 4,17049092 | -0.5[-7.4 : 6.9] | 10,71299569 | 14.6[5.4 : 24.6] | 0,016253857 | 14.2[10.0 : 18.6] | 7,06E-11 | Growth Factor |
| sVCAM-1 | -7.1[-10.6 : -3.5] | 0,001712114 | -12.1[-14.5 : -9.6] | 1,28E-18 | 1.2[-1.6 : 4.0] | 4,989886006 | 0.1[-5.4 : 6.0] | 11,52347102 | 10.1[3.0 : 17.6] | 0,053489416 | 6.9[3.8 : 10.2] | 1,34E-04 | Growth Factor |
| Tie-2 | -3.2[-6.8 : 0.5] | 1,037344519 | -6.7[-9.3 : -4.1] | 9,86E-06 | -1.6[-4.4 : 1.2] | 2,979688084 | 4.4[-1.5 : 10.7] | 1,79963823 | 3.8[-3.0 : 11.1] | 3,337312464 | 4.0[0.9 : 7.2] | 0,127907131 | Growth Factor |
| TSLP | 8.3[-6.2 : 25.1] | 3,307290171 | -6.3[-15.8 : 4.1] | 2,697066977 | -15.2[-23.9 : -5.6] | 0,032249655 | 1.2[-19.1 : 26.6] | 10,9989271 | -1.8[-24.4 : 27.5] | 10,68439791 | 15.5[2.9 : 29.7] | 0,176080152 | Growth Factor |
| VEGF | 33.5[17.5 : 51.6] | 1,09E-04 | 34.6[22.5 : 47.8] | 7,75E-09 | -0.7[-9.8 : 9.2] | 10,61552014 | -3.7[-21.1 : 17.4] | 8,491371127 | 4.3[-17.0 : 31.2] | 8,587246647 | 8.3[-2.2 : 20.0] | 1,514521015 | Growth Factor |
| VEGF-C | 11.3[3.5 : 19.6] | 0,04394069 | 13.6[7.7 : 19.8] | 3,17E-05 | -1.1[-6.3 : 4.5] | 8,421806069 | 4.4[-6.7 : 16.8] | 5,48664029 | 0.1[-12.0 : 14.0] | 11,81065181 | 5.8[-0.1 : 12.2] | 0,660634244 | Growth Factor |
| VEGF-D | 39.7[28.5 : 51.9] | 7,06E-14 | 25.6[18.0 : 33.5] | 6,59E-12 | -5.1[-10.9 : 1.0] | 1,198643334 | -5.5[-17.0 : 7.7] | 4,780237508 | 9.7[-5.6 : 27.4] | 2,738543986 | 1.1[-5.5 : 8.1] | 9,024478667 | Growth Factor |

**Supplementary Table 2:** Main model. Estimates are shown as percentage change and p values are corrected for multiple testing. Model is adjusted for age, current smoking, BMI, sample age, sample analysis date, and Danish administrative region of sampling.

| **Supplementary Table 3** | |  |  |  |  |  |  |  |  |  |  |  |  |
| --- | --- | --- | --- | --- | --- | --- | --- | --- | --- | --- | --- | --- | --- |
|  | COC low |  | COC high |  | IUD |  | POP |  | HT |  | Menopausal |  |  |
| Assay | % estimate | corrected P | % estimate | corrected P | % estimate | corrected P | % estimate | corrected P | % estimate | corrected P | % estimate | corrected P | group |
| CRP | 294.1[238.9 : 358.4] | 2,80E-67 | 325.7[281.3 : 375.1] | 4,83E-135 | 5.7[-5.4 : 18.1] | 3,960351338 | -3.1[-22.9 : 21.7] | 9,421630169 | 34.7[3.5 : 75.3] | 0,319280286 | 17.1[3.9 : 31.8] | 0,11377655 | Proinflammatory |
| SAA | 60.5[41.5 : 82.0] | 2,65E-12 | 41.1[28.7 : 54.6] | 2,79E-12 | -10.1[-18.1 : -1.4] | 0,288356932 | -9.7[-25.3 : 9.2] | 3,527364255 | 38.1[10.9 : 72.0] | 0,047841387 | 20.0[8.6 : 32.5] | 0,003951422 | Proinflammatory |
| IL-1A | -5.0[-14.9 : 6.1] | 4,355810087 | -5.7[-13.0 : 2.3] | 1,884308633 | 0.7[-7.3 : 9.3] | 10,48241871 | -1.7[-17.2 : 16.6] | 10,10380215 | 2.5[-15.8 : 24.9] | 9,664420061 | 4.4[-4.4 : 14.0] | 4,059179881 | Proinflammatory |
| IL-1B | 5.5[-13.4 : 28.5] | 7,11515635 | -0.4[-13.9 : 15.1] | 11,43179641 | 0.6[-13.3 : 16.6] | 11,29170159 | -10.3[-34.1 : 21.9] | 5,843822736 | -2.8[-31.8 : 38.5] | 10,51014295 | 17.7[0.5 : 37.9] | 0,518220135 | Proinflammatory |
| IL-1RA | 3.6[-3.7 : 11.5] | 4,089382906 | -3.1[-8.2 : 2.2] | 2,981661579 | 6.0[0.3 : 12.0] | 0,460838859 | -4.0[-14.3 : 7.6] | 5,757091963 | -4.1[-16.1 : 9.5] | 6,404284938 | 5.3[-0.7 : 11.7] | 1,032585693 | Proinflammatory |
| IL-3 | -1.1[-15.3 : 15.4] | 10,63998977 | -0.1[-10.8 : 12.0] | 11,86051761 | 10.6[-1.5 : 24.2] | 1,062592747 | 0.3[-21.2 : 27.8] | 11,75115766 | -29.1[-46.5 : -6.2] | 0,193451666 | 8.8[-3.9 : 23.2] | 2,212576621 | Proinflammatory |
| IL-6 | -3.2[-13.9 : 8.7] | 6,931424408 | -4.2[-12.1 : 4.4] | 3,94349613 | -6.3[-14.1 : 2.3] | 1,751997315 | 0.8[-15.9 : 20.9] | 11,15321381 | 9.0[-11.5 : 34.3] | 5,026297549 | 22.9[12.0 : 34.9] | 1,80E-04 | Proinflammatory |
| IL-12 | 4.0[-2.7 : 11.2] | 2,985137513 | 1.0[-3.8 : 6.1] | 8,137394018 | -0.3[-5.1 : 4.9] | 11,01874341 | 11.2[0.2 : 23.3] | 0,556488256 | 11.8[-0.9 : 26.0] | 0,827543334 | 9.3[3.6 : 15.3] | 0,013429336 | Proinflammatory |
| IL-12p70 | -5.3[-20.4 : 12.6] | 6,435079433 | -8.5[-19.5 : 4.0] | 2,094631453 | 7.0[-6.1 : 21.9] | 3,717364745 | 3.4[-21.2 : 35.6] | 9,720098012 | 17.5[-14.0 : 60.5] | 3,729892007 | 9.8[-4.5 : 26.2] | 2,258949937 | Proinflammatory |
| IL-15 | 0.3[-3.0 : 3.8] | 10,16201142 | 1.8[-0.7 : 4.4] | 1,862150052 | -4.1[-6.5 : -1.6] | 0,015686733 | -3.4[-8.4 : 1.8] | 2,347591031 | 0.0[-5.8 : 6.3] | 11,86432983 | 0.2[-2.5 : 3.0] | 10,50156641 | Proinflammatory |
| TNF-A | 0.1[-4.2 : 4.5] | 11,71005567 | -3.6[-6.6 : -0.4] | 0,313210021 | -2.4[-5.5 : 0.8] | 1,731002469 | 0.1[-6.5 : 7.1] | 11,72386974 | 14.9[6.3 : 24.2] | 0,006031617 | 10.7[6.9 : 14.6] | 1,50E-07 | Proinflammatory |
| TNF-B | -4.1[-13.3 : 6.2] | 5,046733391 | -7.5[-14.2 : -0.3] | 0,483598773 | -2.9[-10.0 : 4.8] | 5,325633917 | 6.0[-9.5 : 24.2] | 5,64511155 | 5.8[-11.8 : 27.0] | 6,509986641 | 8.7[0.2 : 18.0] | 0,527921992 | Proinflammatory |
| IFN-G | 12.9[1.9 : 25.2] | 0,267524726 | 6.2[-1.6 : 14.6] | 1,584088929 | -2.6[-9.8 : 5.3] | 6,603051705 | 1.5[-13.6 : 19.2] | 11,11487555 | 6.5[-11.5 : 28.1] | 6,599281437 | 9.5[0.8 : 18.9] | 0,419403556 | T derived |
| IL-2 | 5.1[-12.2 : 25.7] | 7,644049739 | 4.0[-8.9 : 18.7] | 7,299674893 | 19.8[4.7 : 37.1] | 0,111788896 | -4.1[-27.5 : 26.9] | 9,995153227 | 72.4[24.9 : 137.9] | 0,012108127 | 12.9[-2.2 : 30.4] | 1,27722623 | T derived |
| IL-4 | 3.8[-12.1 : 22.6] | 8,594705562 | 4.5[-7.6 : 18.1] | 6,315962116 | 0.9[-11.0 : 14.3] | 11,55819328 | 12.6[-13.2 : 46.1] | 4,805542326 | 21.3[-10.1 : 63.7] | 2,679346691 | 8.5[-5.1 : 24.1] | 3,02227727 | T derived |
| IL-5 | 6.5[-2.8 : 16.7] | 2,283891031 | 4.1[-2.7 : 11.4] | 3,13894065 | 2.1[-4.6 : 9.4] | 7,070429366 | -6.0[-18.5 : 8.4] | 5,143349967 | 8.5[-7.9 : 27.9] | 4,256136974 | -1.3[-8.3 : 6.2] | 9,494334675 | T derived |
| IL-9 | -9.0[-23.5 : 8.2] | 3,692883195 | -7.9[-18.9 : 4.6] | 2,68168791 | 5.0[-7.8 : 19.5] | 5,992414128 | -9.4[-30.7 : 18.6] | 6,154222308 | 6.4[-22.2 : 45.6] | 9,080467086 | 20.3[4.7 : 38.2] | 0,119329588 | T derived |
| IL-10 | -8.1[-18.4 : 3.6] | 2,174890015 | -2.2[-10.4 : 6.8] | 8,135998899 | 0.7[-7.9 : 10.2] | 11,43197993 | 17.0[-2.9 : 41.0] | 1,276290273 | 13.5[-8.4 : 40.7] | 3,219322402 | 9.9[-0.2 : 20.9] | 0,712608365 | T derived |
| IL-13 | -10.1[-22.5 : 4.2] | 2,053342807 | 3.3[-7.4 : 15.2] | 7,307727438 | 6.3[-4.9 : 18.8] | 3,637581777 | 10.3[-12.4 : 38.9] | 5,232209169 | -7.6[-29.1 : 20.5] | 7,286555019 | -5.4[-16.0 : 6.5] | 4,644671406 | T derived |
| IL-16 | -0.7[-9.2 : 8.5] | 11,29284285 | -9.7[-15.4 : -3.6] | 0,02826816 | 4.3[-2.4 : 11.5] | 2,784934536 | -1.8[-14.5 : 12.8] | 10,40402245 | 4.4[-10.9 : 22.5] | 7,72277196 | 8.7[1.2 : 16.7] | 0,288072833 | T derived |
| IL-17A | 4.9[-3.9 : 14.6] | 3,721671901 | 2.6[-3.8 : 9.5] | 5,651993601 | 0.7[-5.7 : 7.6] | 10,76998021 | 6.5[-7.2 : 22.2] | 4,816866249 | 22.9[4.9 : 44.0] | 0,138930139 | 11.2[3.6 : 19.4] | 0,042461705 | T derived |
| IL-17A/F | 3.9[-10.1 : 20.0] | 7,878425501 | 5.8[-4.8 : 17.7] | 3,862262842 | -3.6[-13.5 : 7.4] | 6,526329554 | -17.5[-34.1 : 3.1] | 1,186771266 | -10.5[-31.1 : 16.3] | 5,285605207 | -4.0[-14.5 : 7.7] | 6,301684227 | T derived |
| IL-17B | -18.1[-27.7 : -7.3] | 0,021438096 | -14.5[-22.0 : -6.3] | 0,010899618 | -9.2[-17.3 : -0.3] | 0,559831093 | -17.3[-32.0 : 0.4] | 0,720367912 | 17.0[-6.6 : 46.7] | 2,23701524 | 14.3[3.4 : 26.3] | 0,116421566 | T derived |
| IL-17C | 4.1[-9.4 : 19.6] | 7,429951191 | -7.7[-16.7 : 2.3] | 1,633791952 | -7.3[-16.5 : 3.0] | 2,051305885 | -12.2[-29.3 : 9.1] | 3,133012057 | 1.9[-20.9 : 31.2] | 11,50101331 | 1.5[-9.2 : 13.5] | 10,2907733 | T derived |
| IL-17D | 4.8[-7.5 : 18.7] | 5,9757508 | 0.9[-8.0 : 10.6] | 11,04793729 | -2.9[-11.5 : 6.7] | 7,070735528 | 1.5[-16.5 : 23.2] | 11,49570326 | -0.3[-20.5 : 25.0] | 12,70111133 | 16.7[5.6 : 29.0] | 0,032397172 | T derived |
| CCL2 | 7.2[0.7 : 14.1] | 0,292266597 | 1.4[-3.1 : 6.0] | 5,50047826 | -1.5[-5.8 : 3.1] | 5,236668751 | -0.6[-9.6 : 9.3] | 9,003648664 | 18.2[6.2 : 31.6] | 0,02199423 | 16.0[10.5 : 21.8] | 2,35E-08 | Chemokine |
| CCL3 | 0.7[-5.7 : 7.5] | 8,382775239 | 4.3[-0.5 : 9.3] | 0,814360002 | -1.3[-5.8 : 3.5] | 6,001407753 | 8.7[-1.7 : 20.1] | 1,02715569 | 11.8[-0.1 : 25.2] | 0,518333494 | 9.3[3.8 : 15.0] | 0,006691903 | Chemokine |
| CCL4 | -3.5[-10.1 : 3.7] | 3,306229272 | -7.3[-12.0 : -2.4] | 0,037811029 | -2.3[-7.2 : 2.9] | 3,772140065 | -4.0[-13.9 : 7.0] | 4,623263685 | 10.2[-2.5 : 24.5] | 1,215980012 | 8.9[3.0 : 15.2] | 0,025416576 | Chemokine |
| CCL11 | -7.3[-12.4 : -1.8] | 0,095747075 | -11.0[-14.6 : -7.3] | 3,16E-07 | -3.1[-7.1 : 1.0] | 1,314008152 | 2.9[-5.6 : 12.3] | 5,141241546 | 0.6[-8.8 : 11.0] | 9,075770418 | 7.4[2.7 : 12.3] | 0,016409898 | Chemokine |
| CCL13 | -10.0[-16.4 : -3.2] | 0,045387292 | -12.1[-16.6 : -7.3] | 1,68E-05 | 0.7[-4.5 : 6.2] | 7,953877932 | -8.9[-18.5 : 1.8] | 1,007248755 | 5.4[-7.0 : 19.5] | 4,102601013 | 5.7[-0.1 : 11.9] | 0,555520556 | Chemokine |
| CCL17 | -21.4[-29.0 : -12.9] | 3,83E-05 | -19.2[-24.9 : -13.1] | 1,29E-07 | 0.7[-6.4 : 8.4] | 8,505297 | -4.9[-18.6 : 11.0] | 5,221344537 | 13.8[-4.5 : 35.5] | 1,472623146 | 20.5[11.4 : 30.5] | 3,88E-05 | Chemokine |
| CCL22 | 6.1[1.2 : 11.4] | 0,15092186 | -0.4[-3.8 : 3.1] | 8,054466053 | -0.8[-4.2 : 2.7] | 6,482121725 | -2.1[-9.1 : 5.3] | 5,623585097 | 20.8[11.2 : 31.2] | 7,59E-05 | 16.7[12.4 : 21.1] | 8,93E-15 | Chemokine |
| CCL26 | -4.4[-13.8 : 5.9] | 3,875848494 | -7.8[-14.4 : -0.7] | 0,312685349 | -2.0[-9.0 : 5.6] | 5,969873095 | -4.4[-18.3 : 11.8] | 5,720678223 | 1.4[-15.0 : 21.0] | 8,731910213 | -4.3[-11.7 : 3.7] | 2,811641646 | Chemokine |
| IL-8 | 7.6[-3.7 : 20.3] | 1,945655521 | 8.1[-0.4 : 17.3] | 0,624726041 | 1.2[-6.9 : 10.0] | 7,815525345 | -5.1[-20.2 : 12.9] | 5,561028765 | 3.2[-15.4 : 26.0] | 7,532672653 | 9.4[0.1 : 19.6] | 0,474221103 | Chemokine |
| CXCL10 | -2.4[-9.5 : 5.3] | 5,322175877 | -7.2[-12.1 : -1.9] | 0,077801315 | 0.8[-4.6 : 6.5] | 7,853155471 | 0.0[-10.9 : 12.3] | 9,964086465 | 10.7[-2.8 : 26.1] | 1,268562628 | 13.0[6.5 : 19.9] | 5,33E-04 | Chemokine |
| bFGF | 8.6[-3.5 : 22.2] | 2,077289232 | 14.7[5.1 : 25.1] | 0,024074774 | -2.7[-10.9 : 6.4] | 6,610993489 | -11.2[-26.1 : 6.8] | 2,479592906 | 10.3[-10.8 : 36.3] | 4,39235953 | 8.6[-1.3 : 19.4] | 1,078871084 | Growth Factor |
| Flt-1 | -15.7[-19.4 : -12.0] | 2,67E-13 | -15.4[-18.1 : -12.6] | 7,62E-23 | -1.8[-4.9 : 1.5] | 3,489109155 | 2.8[-4.0 : 10.1] | 5,083492505 | -8.8[-15.7 : -1.4] | 0,258086177 | -0.5[-4.0 : 3.0] | 9,151973469 | Growth Factor |
| GM-CSF | -6.7[-18.4 : 6.7] | 3,737555232 | -6.4[-15.1 : 3.4] | 2,304773007 | -3.1[-12.3 : 7.2] | 6,49465148 | 1.2[-17.9 : 24.6] | 10,96255687 | 14.5[-10.0 : 45.6] | 3,231975488 | 9.8[-1.4 : 22.3] | 1,049538665 | Growth Factor |
| IL-7 | 9.1[1.6 : 17.2] | 0,205508613 | 13.0[7.2 : 19.2] | 6,28E-05 | -5.7[-10.6 : -0.5] | 0,386910628 | -4.1[-14.2 : 7.2] | 5,577568984 | -3.2[-14.9 : 10.0] | 7,402656812 | 1.2[-4.4 : 7.2] | 8,117108694 | Growth Factor |
| PlGF | -0.5[-3.9 : 3.1] | 9,58538606 | 2.4[-0.3 : 5.1] | 0,923622385 | -0.8[-3.3 : 1.9] | 6,917779588 | -1.8[-7.1 : 3.7] | 6,155978961 | 8.4[1.7 : 15.4] | 0,157148156 | 9.1[6.1 : 12.3] | 2,00E-08 | Growth Factor |
| sICAM-1 | 4.7[-0.2 : 9.8] | 0,693317993 | 0.9[-2.5 : 4.5] | 7,24403503 | -1.7[-5.1 : 1.8] | 4,076171845 | -0.7[-7.6 : 6.7] | 10,16232549 | 15.3[6.2 : 25.3] | 0,009026583 | 14.2[10.0 : 18.5] | 6,24E-11 | Growth Factor |
| sVCAM-1 | -7.2[-10.6 : -3.6] | 0,001324965 | -12.1[-14.5 : -9.7] | 5,89E-19 | 1.2[-1.6 : 4.0] | 5,040195237 | 0.0[-5.5 : 5.9] | 11,89244504 | 10.5[3.5 : 18.1] | 0,035806393 | 6.9[3.8 : 10.2] | 1,24E-04 | Growth Factor |
| Tie-2 | -3.2[-6.8 : 0.5] | 1,032136698 | -6.6[-9.2 : -4.0] | 1,32E-05 | -1.6[-4.4 : 1.2] | 2,990461552 | 4.3[-1.6 : 10.6] | 1,884991288 | 3.9[-2.9 : 11.1] | 3,179509411 | 4.1[1.0 : 7.3] | 0,100515136 | Growth Factor |
| TSLP | 8.2[-6.3 : 24.8] | 3,381660481 | -6.2[-15.6 : 4.3] | 2,828868925 | -15.1[-23.8 : -5.4] | 0,03498579 | 1.2[-19.0 : 26.5] | 10,97839873 | 0.7[-22.4 : 30.7] | 11,49880934 | 16.4[3.8 : 30.7] | 0,116309512 | Growth Factor |
| VEGF | 35.1[19.0 : 53.5] | 4,50E-05 | 34.4[22.4 : 47.7] | 8,68E-09 | -0.8[-9.9 : 9.1] | 10,36814158 | -2.3[-19.9 : 19.2] | 9,840239302 | 5.6[-16.0 : 32.8] | 7,684983827 | 9.9[-0.8 : 21.7] | 0,855448717 | Growth Factor |
| VEGF-C | 11.7[3.9 : 20.0] | 0,032921971 | 14.3[8.3 : 20.5] | 1,12E-05 | -1.3[-6.5 : 4.2] | 7,613334522 | 4.8[-6.3 : 17.3] | 4,954415103 | 1.0[-11.3 : 15.0] | 10,60513928 | 5.9[0.0 : 12.3] | 0,621976375 | Growth Factor |
| VEGF-D | 39.3[28.0 : 51.6] | 2,22E-13 | 24.5[17.0 : 32.5] | 6,77E-11 | -5.4[-11.2 : 0.8] | 1,02339624 | -6.4[-17.9 : 6.8] | 3,940933978 | 10.5[-5.0 : 28.6] | 2,354083998 | 0.7[-5.9 : 7.8] | 10,00730878 | Growth Factor |

**Supplementary Table 3:** Reduced model adjusted for age, current smoking, BMI, and sample age. Estimates are shown as percentage change and p values are corrected for multiple testing.

| **Supplementary Table 4** | |  |  |  |  |  |  |  |  |  |  |  |  |
| --- | --- | --- | --- | --- | --- | --- | --- | --- | --- | --- | --- | --- | --- |
|  | COC low |  | COC high |  | IUD |  | POP |  | HT |  | Menopausal |  |  |
| Assay | % estimate | corrected P | % estimate | corrected P | % estimate | corrected P | % estimate | corrected P | % estimate | corrected P | % estimate | corrected P | group |
| CRP | 186.7[143.1 : 238.2] | 3,86E-34 | 255.4[216.1 : 299.7] | 2,40E-93 | -3.1[-14.7 : 10.0] | 7,462664928 | -19.7[-38.2 : 4.4] | 1,211305007 | 36.6[2.8 : 81.4] | 0,378000447 | 37.0[25.9 : 49.2] | 4,45E-12 | Proinflammatory |
| SAA | 40.9[24.5 : 59.6] | 7,91E-07 | 31.2[20.1 : 43.3] | 2,26E-08 | -13.2[-21.1 : -4.5] | 0,044859602 | -16.1[-31.1 : 2.1] | 0,961175848 | 38.2[11.6 : 71.1] | 0,036094911 | 26.9[19.1 : 35.3] | 3,43E-12 | Proinflammatory |
| IL-1A | -3.1[-12.6 : 7.6] | 6,707699614 | -4.6[-11.5 : 2.9] | 2,671825596 | -0.5[-8.3 : 7.9] | 10,8128559 | -2.2[-17.5 : 16.0] | 9,576550151 | -2.1[-18.6 : 17.7] | 9,835168696 | 0.1[-5.3 : 5.7] | 11,81028549 | Proinflammatory |
| IL-1B | -2.6[-19.2 : 17.5] | 9,401989275 | -3.5[-15.7 : 10.5] | 7,266530329 | 1.5[-12.3 : 17.6] | 10,04844367 | -11.9[-35.1 : 19.6] | 4,994452109 | 1.4[-27.2 : 41.3] | 11,20110288 | 26.5[14.7 : 39.6] | 3,27E-05 | Proinflammatory |
| IL-1RA | -4.0[-10.9 : 3.3] | 3,267814529 | -5.9[-10.7 : -0.7] | 0,309289708 | 3.8[-2.0 : 9.9] | 2,492164686 | -8.1[-18.5 : 3.6] | 2,00527587 | -8.7[-20.0 : 4.2] | 2,127219059 | 4.4[0.5 : 8.6] | 0,332569207 | Proinflammatory |
| IL-3 | -4.2[-17.2 : 10.9] | 6,796723541 | 1.2[-9.0 : 12.4] | 9,943042052 | 13.2[1.0 : 27.0] | 0,406588607 | 2.5[-19.4 : 30.4] | 10,05185953 | -30.2[-46.3 : -9.2] | 0,08866518 | 8.4[0.4 : 17.1] | 0,475799746 | Proinflammatory |
| IL-6 | -17.4[-26.7 : -6.9] | 0,02071416 | -12.2[-19.4 : -4.3] | 0,037890049 | -10.7[-18.7 : -2.0] | 0,207242245 | -9.5[-25.5 : 10.1] | 3,814472734 | 5.9[-14.3 : 30.9] | 7,126051162 | 29.5[21.7 : 37.9] | 8,15E-15 | Proinflammatory |
| IL-12 | 7.9[1.0 : 15.2] | 0,293651885 | 4.6[-0.2 : 9.7] | 0,748167093 | -2.0[-6.9 : 3.2] | 5,315233295 | 12.2[0.7 : 24.9] | 0,44188503 | -2.9[-13.6 : 9.1] | 7,457299051 | -4.9[-8.2 : -1.6] | 0,050213653 | Proinflammatory |
| IL-12p70 | -3.4[-18.0 : 13.9] | 8,209516808 | -6.2[-16.7 : 5.7] | 3,51399672 | 6.6[-6.3 : 21.3] | 3,937818189 | 4.2[-20.4 : 36.5] | 9,149470258 | 10.0[-17.8 : 47.3] | 6,244040718 | 3.6[-5.0 : 12.9] | 5,065606065 | Proinflammatory |
| IL-15 | -1.8[-4.9 : 1.5] | 3,334494047 | 0.4[-1.9 : 2.8] | 8,747083816 | -3.5[-5.9 : -1.0] | 0,073346502 | -3.9[-8.8 : 1.4] | 1,729873125 | 4.5[-1.4 : 10.6] | 1,628535003 | 5.0[3.2 : 6.8] | 2,30E-07 | Proinflammatory |
| TNF-A | -1.8[-5.8 : 2.4] | 4,755915284 | -3.5[-6.4 : -0.6] | 0,241598196 | -2.8[-5.9 : 0.5] | 1,096681086 | -0.5[-7.1 : 6.6] | 10,60379436 | 10.7[2.8 : 19.3] | 0,088008547 | 8.4[6.0 : 10.8] | 1,04E-11 | Proinflammatory |
| TNF-B | 1.8[-7.6 : 12.1] | 8,673448741 | -1.9[-8.5 : 5.1] | 6,960788238 | -3.6[-10.6 : 4.0] | 4,16210552 | 9.0[-7.0 : 27.6] | 3,44169336 | -9.0[-23.3 : 8.0] | 3,362868128 | -6.3[-10.9 : -1.4] | 0,138832957 | Proinflammatory |
| IFN-G | 6.2[-3.7 : 17.2] | 2,931411925 | 1.0[-5.9 : 8.3] | 10,27139109 | -3.0[-10.2 : 4.7] | 5,675488172 | -1.8[-16.3 : 15.3] | 10,74875961 | 15.8[-2.6 : 37.7] | 1,268042454 | 20.1[14.1 : 26.5] | 3,56E-11 | T derived |
| IL-2 | 11.5[-6.0 : 32.2] | 2,740157793 | 4.3[-7.8 : 17.9] | 6,546637307 | 16.0[1.5 : 32.6] | 0,381985382 | -5.8[-28.7 : 24.5] | 8,793436524 | 66.6[23.2 : 125.4] | 0,012048202 | 6.9[-2.3 : 16.8] | 1,892608641 | T derived |
| IL-4 | 4.4[-10.9 : 22.3] | 7,728479739 | 6.6[-4.9 : 19.5] | 3,521116933 | 0.9[-10.9 : 14.2] | 11,5839892 | 13.7[-12.2 : 47.2] | 4,309319177 | 13.7[-14.1 : 50.5] | 4,791789023 | 2.7[-5.5 : 11.5] | 6,965682721 | T derived |
| IL-5 | 1.7[-6.7 : 10.9] | 9,117331302 | -0.1[-6.2 : 6.3] | 12,53741797 | 2.1[-4.6 : 9.2] | 7,189471646 | -8.3[-20.4 : 5.7] | 3,034686122 | 18.2[1.3 : 37.8] | 0,430405622 | 7.9[3.1 : 12.9] | 0,014120519 | T derived |
| IL-9 | -14.0[-27.1 : 1.3] | 0,920467729 | -13.0[-22.7 : -2.1] | 0,274714437 | 5.3[-7.4 : 19.7] | 5,645637319 | -11.7[-32.4 : 15.3] | 4,681240076 | 20.7[-10.0 : 62.0] | 2,713060831 | 35.9[24.7 : 48.1] | 4,60E-11 | T derived |
| IL-10 | -2.0[-12.5 : 9.8] | 9,413715027 | 2.7[-5.4 : 11.5] | 6,771179805 | 0.5[-8.0 : 9.9] | 11,82820293 | 20.4[0.0 : 45.0] | 0,647902978 | 3.1[-15.7 : 26.0] | 9,998960945 | -1.3[-7.0 : 4.8] | 8,766157412 | T derived |
| IL-13 | -12.1[-23.6 : 1.2] | 0,939124109 | -0.7[-10.2 : 9.9] | 11,6385665 | 4.5[-6.3 : 16.7] | 5,568700882 | 6.6[-15.2 : 34.1] | 7,58003195 | -2.0[-23.6 : 25.6] | 11,35007153 | 0.2[-6.9 : 7.8] | 12,58127718 | T derived |
| IL-16 | 0.1[-8.0 : 8.9] | 12,80206396 | -5.4[-11.0 : 0.5] | 0,963099779 | 6.6[-0.3 : 13.9] | 0,784569375 | 2.1[-11.1 : 17.2] | 10,03735811 | -4.4[-17.7 : 11.1] | 7,238226121 | 1.0[-3.4 : 5.6] | 8,655160726 | T derived |
| IL-17A | 10.2[1.2 : 19.9] | 0,329784907 | 8.1[1.7 : 14.9] | 0,166119068 | -0.8[-7.1 : 6.1] | 10,70678338 | 8.4[-5.7 : 24.5] | 3,319489325 | 3.8[-10.7 : 20.6] | 8,182289312 | -5.5[-9.7 : -1.3] | 0,154803082 | T derived |
| IL-17A/F | -2.5[-15.0 : 11.8] | 9,337224062 | 3.4[-6.3 : 14.1] | 6,640883681 | -0.6[-10.7 : 10.6] | 11,86915298 | -16.6[-33.3 : 4.2] | 1,435927631 | -2.0[-23.3 : 25.2] | 11,29496178 | 5.5[-1.9 : 13.3] | 1,916926572 | T derived |
| IL-17B | -18.3[-27.4 : -8.0] | 0,011261058 | -13.7[-20.8 : -6.0] | 0,009794265 | -6.8[-15.1 : 2.3] | 1,770603921 | -14.6[-29.7 : 3.7] | 1,446436222 | 20.1[-3.0 : 48.5] | 1,199343122 | 15.6[8.6 : 23.0] | 7,63E-05 | T derived |
| IL-17C | 0.3[-12.1 : 14.4] | 12,57114448 | -8.9[-17.2 : 0.2] | 0,711332355 | -6.7[-15.8 : 3.5] | 2,485946946 | -12.5[-29.5 : 8.5] | 2,903485634 | 3.6[-18.3 : 31.3] | 10,01928726 | 4.2[-2.8 : 11.7] | 3,14979917 | T derived |
| IL-17D | -12.7[-22.6 : -1.7] | 0,3307138 | -14.2[-21.3 : -6.5] | 0,006248467 | -0.7[-9.5 : 9.1] | 11,57070915 | -5.5[-22.3 : 14.8] | 7,371174561 | 48.3[19.6 : 83.8] | 0,004212114 | 73.7[63.1 : 84.9] | 3,14E-63 | T derived |
| CCL2 | 1.2[-4.6 : 7.4] | 6,842006197 | -2.8[-6.7 : 1.4] | 1,880803548 | -0.8[-5.1 : 3.8] | 7,326172101 | -2.4[-11.2 : 7.4] | 6,237766038 | 30.0[17.6 : 43.8] | 3,16E-06 | 28.4[24.6 : 32.3] | 1,24E-56 | Chemokine |
| CCL3 | -3.9[-9.7 : 2.4] | 2,170593436 | 0.8[-3.5 : 5.4] | 7,077830302 | -2.3[-6.8 : 2.5] | 3,460266736 | 5.7[-4.4 : 16.9] | 2,786382638 | 15.6[3.9 : 28.5] | 0,076170655 | 14.7[11.1 : 18.4] | 4,86E-16 | Chemokine |
| CCL4 | -8.2[-14.3 : -1.8] | 0,13265115 | -10.7[-14.9 : -6.3] | 3,64E-05 | -2.5[-7.4 : 2.6] | 3,250767835 | -6.1[-15.8 : 4.7] | 2,580257763 | 16.7[4.0 : 30.9] | 0,0851122 | 16.5[12.5 : 20.6] | 5,44E-17 | Chemokine |
| CCL11 | -16.6[-21.2 : -11.8] | 2,72E-09 | -19.1[-22.2 : -15.8] | 1,94E-24 | -1.4[-5.5 : 2.9] | 5,246268411 | -0.7[-9.3 : 8.6] | 8,735477883 | 29.3[17.6 : 42.2] | 1,23E-06 | 37.8[33.9 : 41.8] | 7,64E-101 | Chemokine |
| CCL13 | -20.8[-26.2 : -15.0] | 9,91E-10 | -20.2[-24.0 : -16.1] | 6,42E-18 | 1.1[-4.2 : 6.6] | 6,937887929 | -13.3[-22.5 : -2.9] | 0,131540497 | 27.1[12.8 : 43.2] | 7,95E-04 | 29.7[25.1 : 34.4] | 4,34E-44 | Chemokine |
| CCL17 | -23.1[-30.3 : -15.2] | 1,38E-06 | -18.6[-24.0 : -12.8] | 4,48E-08 | 1.5[-5.8 : 9.2] | 7,001400934 | -4.7[-18.5 : 11.4] | 5,435945939 | 11.9[-5.1 : 32.0] | 1,820766269 | 21.2[15.4 : 27.4] | 2,97E-13 | Chemokine |
| CCL22 | 7.4[2.4 : 12.6] | 0,035135166 | 2.8[-0.6 : 6.3] | 1,089085786 | -2.1[-5.6 : 1.5] | 2,394735737 | -2.2[-9.4 : 5.6] | 5,70167429 | 6.9[-1.4 : 15.9] | 1,035682997 | 6.1[3.5 : 8.7] | 1,82E-05 | Chemokine |
| CCL26 | -6.4[-15.1 : 3.2] | 1,843626333 | -7.1[-13.3 : -0.5] | 0,350571343 | -0.9[-8.0 : 6.6] | 8,024405408 | -3.4[-17.4 : 12.9] | 6,606192389 | -0.5[-15.6 : 17.4] | 9,563385376 | -4.9[-9.5 : -0.1] | 0,459978431 | Chemokine |
| IL-8 | 0.4[-9.7 : 11.5] | 9,471348887 | 0.4[-7.0 : 8.3] | 9,229103845 | 0.6[-7.4 : 9.2] | 8,924782302 | -9.4[-23.8 : 7.6] | 2,602621236 | 20.6[0.0 : 45.4] | 0,502004878 | 28.1[21.2 : 35.4] | 2,21E-17 | Chemokine |
| CXCL10 | -8.7[-15.1 : -1.8] | 0,146451477 | -12.0[-16.3 : -7.3] | 1,09E-05 | 0.1[-5.3 : 5.7] | 9,770887557 | -3.1[-13.8 : 8.8] | 5,903272504 | 19.5[5.7 : 35.1] | 0,045693559 | 23.1[18.7 : 27.8] | 4,64E-27 | Chemokine |
| bFGF | -0.7[-11.2 : 11.1] | 10,85029085 | 10.5[1.9 : 19.8] | 0,191826523 | -0.5[-8.9 : 8.7] | 10,95845515 | -11.9[-26.7 : 5.9] | 2,121245429 | 19.8[-1.8 : 46.3] | 0,896766326 | 20.4[13.5 : 27.7] | 9,34E-09 | Growth Factor |
| Flt-1 | -17.8[-21.1 : -14.3] | 6,62E-19 | -15.6[-18.1 : -13.0] | 7,85E-27 | -0.6[-3.8 : 2.8] | 8,873829752 | 3.6[-3.2 : 10.9] | 3,697780736 | -8.0[-14.5 : -0.9] | 0,327393303 | 1.0[-1.2 : 3.2] | 4,42189778 | Growth Factor |
| GM-CSF | -2.4[-14.1 : 10.8] | 8,460245971 | -1.9[-10.4 : 7.5] | 8,248000277 | -3.7[-12.8 : 6.4] | 5,519995987 | 3.2[-16.1 : 27.0] | 9,176123565 | 1.0[-19.3 : 26.5] | 11,16972808 | -2.7[-8.9 : 4.0] | 5,06559596 | Growth Factor |
| IL-7 | 5.1[-1.9 : 12.5] | 1,863401491 | 12.4[7.0 : 18.1] | 3,66E-05 | -5.0[-9.9 : 0.2] | 0,721982627 | -4.1[-14.2 : 7.2] | 5,543212443 | -4.0[-15.0 : 8.3] | 6,035688999 | 2.0[-1.6 : 5.7] | 3,451415474 | Growth Factor |
| PlGF | -5.4[-8.6 : -2.2] | 0,015398552 | -1.6[-3.9 : 0.9] | 2,502431634 | -0.7[-3.3 : 2.0] | 7,168523046 | -4.1[-9.3 : 1.4] | 1,669380131 | 16.8[10.0 : 24.0] | 5,33E-06 | 18.5[16.5 : 20.7] | 3,05E-74 | Growth Factor |
| sICAM-1 | 3.1[-1.5 : 7.9] | 2,346432782 | 1.3[-2.0 : 4.6] | 5,424926492 | -2.2[-5.6 : 1.3] | 2,651889971 | -1.3[-8.2 : 6.2] | 8,687791588 | 11.1[2.7 : 20.3] | 0,107053529 | 12.0[9.4 : 14.7] | 7,16E-20 | Growth Factor |
| sVCAM-1 | -5.4[-8.7 : -1.9] | 0,032352956 | -10.5[-12.8 : -8.2] | 3,23E-16 | 0.9[-1.9 : 3.7] | 6,583816001 | 0.8[-4.8 : 6.7] | 9,373047185 | 5.1[-1.2 : 11.9] | 1,353677039 | 1.7[-0.1 : 3.6] | 0,803097309 | Growth Factor |
| Tie-2 | -2.3[-5.7 : 1.2] | 2,297875462 | -5.8[-8.2 : -3.4] | 4,81E-05 | -1.4[-4.1 : 1.4] | 3,853928454 | 5.1[-0.8 : 11.4] | 1,111740512 | 2.6[-3.7 : 9.2] | 5,153248235 | 2.5[0.6 : 4.4] | 0,106963818 | Growth Factor |
| TSLP | -7.1[-19.0 : 6.5] | 3,505846512 | -15.7[-23.6 : -6.9] | 0,008633633 | -15.3[-24.0 : -5.7] | 0,029769158 | -5.6[-24.6 : 18.1] | 7,358384431 | 20.6[-5.8 : 54.3] | 1,645986862 | 43.7[33.7 : 54.4] | 1,33E-21 | Growth Factor |
| VEGF | 17.0[3.6 : 32.1] | 0,139848469 | 23.1[12.7 : 34.4] | 4,27E-05 | 1.1[-8.1 : 11.2] | 9,827462006 | -6.1[-23.1 : 14.5] | 6,390938374 | 27.2[2.5 : 57.9] | 0,344390678 | 35.9[27.5 : 44.8] | 9,19E-20 | Growth Factor |
| VEGF-C | 5.0[-2.0 : 12.5] | 1,945749885 | 10.8[5.4 : 16.4] | 6,58E-04 | -0.2[-5.5 : 5.3] | 11,18585239 | 3.5[-7.5 : 15.9] | 6,521859686 | 7.7[-4.7 : 21.7] | 2,789183713 | 14.5[10.5 : 18.7] | 2,45E-12 | Growth Factor |
| VEGF-D | 39.1[28.2 : 50.8] | 2,19E-14 | 20.6[13.7 : 27.8] | 4,32E-09 | -5.5[-11.3 : 0.7] | 0,97597523 | -7.8[-19.3 : 5.2] | 2,723575262 | 23.8[7.2 : 42.9] | 0,042777631 | 10.9[6.3 : 15.8] | 2,01E-05 | Growth Factor |

**Supplementary Table 4:** Unadjusted model. Estimates are shown as percentage change and p values are corrected for multiple testing.

| **Supplementary Table 5** | |  |  |  |  |  |  |  |  |  |  |
| --- | --- | --- | --- | --- | --- | --- | --- | --- | --- | --- | --- |
|  | COC low+high |  | IUD |  | POP |  | HT |  | Menopausal |  |  |
| Assay | % estimate | corrected P | % estimate | corrected P | % estimate | corrected P | % estimate | corrected P | % estimate | corrected P | Group |
| CRP | 315.8[276.3 : 359.5] | 4,24E-156 | 5.1[-5.9 : 17.5] | 4,546550926 | -3.9[-23.5 : 20.8] | 8,8308397 | 32.5[1.8 : 72.6] | 0,438873288 | 16.5[3.4 : 31.3] | 0,144682487 | Proinflammatory |
| SAA | 46.9[35.2 : 59.7] | 2,65E-18 | -10.5[-18.4 : -1.7] | 0,237420187 | -9.4[-25.1 : 9.6] | 3,716374658 | 36.5[9.5 : 70.2] | 0,067836195 | 20.1[8.7 : 32.7] | 0,003735888 | Proinflammatory |
| IL-1A | -5.5[-12.1 : 1.7] | 1,610409328 | 0.7[-7.3 : 9.4] | 10,38178531 | -1.3[-16.9 : 17.1] | 10,53640335 | 2.1[-16.2 : 24.4] | 10,05625659 | 4.5[-4.4 : 14.2] | 3,957034445 | Proinflammatory |
| IL-1B | 0.9[-11.5 : 15.1] | 10,72214381 | 0.9[-12.9 : 17.0] | 10,85647637 | -12.3[-35.5 : 19.2] | 4,817681152 | -0.9[-30.4 : 41.1] | 11,50174691 | 15.9[-1.1 : 35.8] | 0,816154245 | Proinflammatory |
| IL-1RA | -2.4[-6.9 : 2.4] | 3,93803468 | 5.6[0.1 : 11.5] | 0,560353873 | -5.9[-15.9 : 5.2] | 3,381367584 | -4.7[-16.3 : 8.6] | 5,683918753 | 3.5[-2.3 : 9.7] | 2,858485877 | Proinflammatory |
| IL-3 | -0.5[-10.3 : 10.3] | 11,09816698 | 11.5[-0.7 : 25.3] | 0,7908477 | 0.6[-21.1 : 28.2] | 11,55066019 | -27.6[-45.4 : -4.0] | 0,29651336 | 9.2[-3.6 : 23.7] | 2,006612144 | Proinflammatory |
| IL-6 | -3.6[-10.8 : 4.2] | 4,2993015 | -5.8[-13.7 : 2.8] | 2,142734732 | 0.9[-15.9 : 21.0] | 11,08375871 | 9.2[-11.4 : 34.6] | 4,909522744 | 23.7[12.6 : 35.8] | 1,08E-04 | Proinflammatory |
| IL-12 | 2.0[-2.4 : 6.7] | 4,509641013 | -0.4[-5.2 : 4.8] | 10,63677667 | 11.0[0.1 : 23.2] | 0,58606052 | 12.0[-0.6 : 26.3] | 0,763674329 | 9.1[3.4 : 15.2] | 0,017240894 | Proinflammatory |
| IL-12p70 | -8.0[-18.1 : 3.3] | 1,888211024 | 6.7[-6.4 : 21.6] | 3,992995946 | 3.1[-21.4 : 35.3] | 9,879422525 | 18.3[-13.5 : 61.8] | 3,502794245 | 9.1[-5.1 : 25.6] | 2,647396933 | Proinflammatory |
| IL-15 | 1.5[-0.8 : 3.8] | 2,469780784 | -4.0[-6.4 : -1.6] | 0,017784189 | -3.0[-8.0 : 2.3] | 3,15897188 | -0.1[-6.0 : 6.2] | 11,69018124 | 0.3[-2.3 : 3.1] | 9,658461146 | Proinflammatory |
| TNF-A | -2.4[-5.2 : 0.4] | 1,159597876 | -2.1[-5.3 : 1.1] | 2,331530186 | 0.1[-6.5 : 7.1] | 11,82416773 | 14.9[6.2 : 24.3] | 0,006219318 | 10.7[6.9 : 14.6] | 1,77E-07 | Proinflammatory |
| TNF-B | -7.0[-13.1 : -0.4] | 0,448099406 | -3.4[-10.5 : 4.3] | 4,506408048 | 5.1[-10.3 : 23.2] | 6,424363775 | 5.4[-12.2 : 26.5] | 6,900335917 | 8.5[0.0 : 17.8] | 0,599783442 | Proinflammatory |
| IFN-G | 8.8[1.5 : 16.5] | 0,221723699 | -2.8[-10.0 : 5.1] | 6,238175405 | 1.9[-13.3 : 19.7] | 10,68084117 | 6.4[-11.7 : 28.1] | 6,694170629 | 9.3[0.6 : 18.8] | 0,467468897 | T derived |
| IL-2 | 5.4[-6.5 : 18.8] | 5,097542683 | 20.1[4.9 : 37.4] | 0,101701093 | -4.0[-27.4 : 27.1] | 10,10471397 | 73.5[25.6 : 139.6] | 0,010853611 | 13.1[-2.2 : 30.7] | 1,250990886 | T derived |
| IL-4 | 3.3[-7.6 : 15.5] | 7,354241834 | 0.1[-11.7 : 13.4] | 12,90084328 | 10.4[-14.9 : 43.3] | 5,919854345 | 18.7[-12.1 : 60.2] | 3,420645291 | 7.2[-6.3 : 22.6] | 4,014809635 | T derived |
| IL-5 | 5.0[-1.2 : 11.6] | 1,491258111 | 2.3[-4.5 : 9.6] | 6,744127383 | -4.9[-17.5 : 9.7] | 6,383530172 | 7.2[-9.1 : 26.3] | 5,300899554 | -0.7[-7.8 : 6.8] | 10,99435578 | T derived |
| IL-9 | -8.4[-18.4 : 2.8] | 1,743348306 | 4.5[-8.2 : 19.0] | 6,582101321 | -9.0[-30.5 : 19.0] | 6,375149782 | 8.9[-20.4 : 49.2] | 7,710669873 | 20.1[4.5 : 38.1] | 0,131159815 | T derived |
| IL-10 | -4.0[-11.3 : 4.0] | 4,190428197 | 0.4[-8.2 : 9.9] | 12,03653813 | 17.6[-2.5 : 41.8] | 1,166265129 | 12.8[-9.0 : 40.0] | 3,539839073 | 9.1[-0.9 : 20.2] | 0,995486989 | T derived |
| IL-13 | -0.6[-10.0 : 9.7] | 11,73836675 | 5.9[-5.2 : 18.4] | 4,041881344 | 9.0[-13.5 : 37.3] | 6,064291924 | -8.3[-29.7 : 19.7] | 6,798745246 | -7.1[-17.5 : 4.6] | 2,927576733 | T derived |
| IL-16 | -8.3[-13.4 : -2.9] | 0,03858392 | 4.8[-1.7 : 11.7] | 1,991130677 | -4.6[-16.5 : 9.0] | 6,311441103 | 2.8[-11.8 : 19.9] | 9,357859037 | 6.8[-0.3 : 14.4] | 0,792082876 | T derived |
| IL-17A | 3.6[-2.4 : 9.9] | 3,183918658 | 0.6[-5.8 : 7.6] | 11,05303149 | 7.0[-6.8 : 22.8] | 4,381438242 | 23.0[4.9 : 44.2] | 0,139528527 | 10.8[3.2 : 19.0] | 0,062500938 | T derived |
| IL-17A/F | 5.5[-4.2 : 16.1] | 3,608616339 | -3.3[-13.2 : 7.8] | 7,07181603 | -16.6[-33.3 : 4.4] | 1,469739919 | -9.5[-30.4 : 17.6] | 5,891725013 | -3.0[-13.6 : 9.0] | 7,916912077 | T derived |
| IL-17B | -16.0[-22.7 : -8.7] | 5,07E-04 | -9.7[-17.7 : -0.8] | 0,428391942 | -17.6[-32.2 : 0.1] | 0,661240773 | 14.6[-8.6 : 43.7] | 3,079946149 | 13.5[2.6 : 25.4] | 0,177789602 | T derived |
| IL-17C | -4.7[-13.1 : 4.6] | 4,068018591 | -7.7[-16.9 : 2.5] | 1,718758397 | -12.4[-29.5 : 8.8] | 3,000037194 | 1.7[-21.1 : 31.0] | 11,6677289 | 1.6[-9.2 : 13.6] | 10,22199003 | T derived |
| IL-17D | 2.2[-5.9 : 11.1] | 7,807568378 | -2.5[-11.2 : 7.1] | 7,838702576 | 3.1[-15.1 : 25.2] | 9,8410018 | -0.1[-20.4 : 25.3] | 12,87842985 | 17.4[6.2 : 29.8] | 0,022490132 | T derived |
| CCL2 | 3.1[-1.0 : 7.4] | 1,384736624 | -0.9[-5.3 : 3.7] | 6,925185209 | -0.1[-9.1 : 9.9] | 9,8865441 | 16.8[5.0 : 30.1] | 0,044420019 | 16.2[10.7 : 22.0] | 1,42E-08 | Chemokine |
| CCL3 | 3.5[-0.9 : 8.0] | 1,182388165 | -1.0[-5.6 : 3.8] | 6,687085047 | 8.5[-1.9 : 19.9] | 1,110249128 | 10.3[-1.5 : 23.4] | 0,886162236 | 8.7[3.3 : 14.4] | 0,014378671 | Chemokine |
| CCL4 | -6.5[-10.8 : -2.0] | 0,05145457 | -2.2[-7.1 : 3.0] | 4,071406979 | -4.7[-14.6 : 6.3] | 3,881415325 | 9.5[-3.2 : 23.8] | 1,487363267 | 8.8[2.9 : 15.0] | 0,031490957 | Chemokine |
| CCL11 | -9.7[-13.1 : -6.3] | 9,02E-07 | -3.0[-6.9 : 1.1] | 1,517512816 | 3.9[-4.8 : 13.4] | 3,916497113 | 0.2[-9.1 : 10.6] | 9,605073662 | 7.8[3.1 : 12.7] | 0,009438475 | Chemokine |
| CCL13 | -12.2[-16.3 : -7.9] | 1,01E-06 | 0.5[-4.6 : 6.0] | 8,457083668 | -9.4[-19.0 : 1.2] | 0,799453439 | 5.0[-7.3 : 19.0] | 4,443626964 | 4.8[-1.0 : 10.9] | 1,036842841 | Chemokine |
| CCL17 | -20.3[-25.4 : -14.8] | 2,80E-10 | 0.8[-6.4 : 8.5] | 8,378574143 | -6.0[-19.5 : 9.7] | 4,326965647 | 13.2[-5.0 : 34.8] | 1,646046484 | 19.7[10.6 : 29.5] | 9,18E-05 | Chemokine |
| CCL22 | 1.4[-1.7 : 4.7] | 3,731035942 | -0.9[-4.3 : 2.7] | 6,24854974 | -2.1[-9.0 : 5.4] | 5,804375279 | 20.0[10.4 : 30.4] | 1,74E-04 | 16.3[12.0 : 20.8] | 5,10E-14 | Chemokine |
| CCL26 | -6.3[-12.4 : 0.2] | 0,584337519 | -1.3[-8.3 : 6.4] | 7,389748215 | -4.3[-18.2 : 12.0] | 5,843544527 | 2.3[-14.3 : 22.1] | 8,027098121 | -3.5[-10.9 : 4.6] | 3,857332885 | Chemokine |
| IL-8 | 5.8[-1.4 : 13.5] | 1,185700791 | 1.6[-6.1 : 10.0] | 6,951861357 | -9.5[-23.2 : 6.7] | 2,336083827 | 1.5[-16.0 : 22.7] | 8,781043487 | 5.9[-2.7 : 15.3] | 1,86304292 | Chemokine |
| CXCL10 | -5.9[-10.5 : -1.1] | 0,16993336 | 0.9[-4.6 : 6.6] | 7,616704108 | 0.5[-10.5 : 12.9] | 9,34288517 | 10.1[-3.4 : 25.5] | 1,484688842 | 12.6[6.1 : 19.4] | 9,57E-04 | Chemokine |
| bFGF | 11.9[3.5 : 21.1] | 0,059309428 | -2.6[-10.8 : 6.5] | 6,796359261 | -11.8[-26.6 : 5.9] | 2,14906271 | 10.0[-11.0 : 36.0] | 4,521148181 | 7.0[-2.6 : 17.7] | 1,916767078 | Growth Factor |
| Flt-1 | -15.5[-17.9 : -13.0] | 4,44E-28 | -1.6[-4.8 : 1.7] | 4,058146609 | 3.1[-3.7 : 10.4] | 4,559570238 | -8.3[-15.2 : -0.8] | 0,375136328 | -0.4[-3.8 : 3.2] | 9,99032075 | Growth Factor |
| GM-CSF | -6.2[-14.2 : 2.6] | 1,955371547 | -3.2[-12.5 : 7.0] | 6,270955622 | 2.1[-17.2 : 25.9] | 10,12488306 | 12.9[-11.3 : 43.7] | 3,878954984 | 10.0[-1.3 : 22.5] | 1,017711697 | Growth Factor |
| IL-7 | 11.5[6.3 : 16.8] | 7,65E-05 | -5.8[-10.7 : -0.7] | 0,321624396 | -5.0[-14.9 : 6.0] | 4,323061719 | -4.2[-15.6 : 8.8] | 6,110102147 | -0.1[-5.6 : 5.8] | 11,75560252 | Growth Factor |
| PlGF | 1.3[-1.0 : 3.8] | 3,177642672 | -0.7[-3.3 : 1.9] | 7,080930421 | -2.0[-7.2 : 3.6] | 5,675177226 | 8.4[1.7 : 15.5] | 0,155175123 | 8.9[5.8 : 12.0] | 5,65E-08 | Growth Factor |
| sICAM-1 | 2.2[-1.0 : 5.4] | 2,202334927 | -1.6[-5.0 : 1.9] | 4,342525286 | -0.4[-7.4 : 7.0] | 10,83222134 | 14.6[5.5 : 24.6] | 0,016064172 | 14.3[10.0 : 18.7] | 5,82E-11 | Growth Factor |
| sVCAM-1 | -10.7[-12.9 : -8.4] | 2,07E-17 | 1.2[-1.5 : 4.1] | 4,645344084 | 0.2[-5.4 : 6.1] | 11,29966498 | 10.1[3.1 : 17.6] | 0,052674896 | 7.0[3.9 : 10.3] | 1,07E-04 | Growth Factor |
| Tie-2 | -5.7[-8.1 : -3.3] | 5,16E-05 | -1.6[-4.3 : 1.2] | 3,158297428 | 4.4[-1.5 : 10.8] | 1,750239398 | 3.8[-2.9 : 11.1] | 3,29604239 | 4.1[1.0 : 7.3] | 0,115279695 | Growth Factor |
| TSLP | -2.2[-11.1 : 7.6] | 7,764404671 | -15.1[-23.8 : -5.4] | 0,036366625 | 1.4[-19.0 : 26.8] | 10,86536295 | -1.7[-24.3 : 27.7] | 10,76196121 | 15.8[3.1 : 30.0] | 0,158569141 | Growth Factor |
| VEGF-A | 34.2[23.3 : 46.1] | 1,45E-10 | -0.7[-9.8 : 9.2] | 10,59330062 | -3.7[-21.1 : 17.4] | 8,482148688 | 4.3[-17.0 : 31.2] | 8,591518821 | 8.3[-2.2 : 20.0] | 1,520056843 | Growth Factor |
| VEGF-C | 12.9[7.6 : 18.5] | 9,05E-06 | -1.1[-6.3 : 4.4] | 8,329067329 | 4.3[-6.8 : 16.8] | 5,517868727 | 0.1[-12.1 : 14.0] | 11,83318011 | 5.8[-0.2 : 12.1] | 0,675974329 | Growth Factor |
| VEGF-D | 29.6[22.6 : 37.0] | 1,40E-18 | -5.0[-10.8 : 1.2] | 1,318427407 | -5.3[-16.9 : 7.8] | 4,915178048 | 9.8[-5.6 : 27.5] | 2,693252717 | 1.3[-5.3 : 8.3] | 8,600664087 | Growth Factor |

**Supplementary Table 5:** Model combining COC low and COC high into a group with all COC users. Estimates are shown as percentage change and p values are corrected for multiple testing. Model is adjusted for age, current smoking, BMI, sample age, sample analysis date, and Danish administrative region of sampling.

| **Supplementary Table 6** | |  |  |  |
| --- | --- | --- | --- | --- |
| assay_list | COC high compared to COC low | p | corrected P | group |
| CRP | 23.7[3.5 : 47.8] | 0,01935787 | 0,232294435 | Proinflammatory |
| SAA | -6.3[-19.9 : 9.6] | 0,414931145 | 4,979173738 | Proinflammatory |
| IL-1A | -1.3[-12.7 : 11.6] | 0,832407368 | 9,988888413 | Proinflammatory |
| IL-1B | -0.1[-20.2 : 25.2] | 0,994966036 | 11,93959243 | Proinflammatory |
| IL-1RA | -4.7[-12.8 : 4.2] | 0,288115032 | 3,457380389 | Proinflammatory |
| IL-3 | -0.3[-16.8 : 19.6] | 0,976198742 | 11,7143849 | Proinflammatory |
| IL-6 | 1.4[-12.2 : 17.0] | 0,851843209 | 10,22211851 | Proinflammatory |
| IL-12 | -1.7[-8.9 : 6.0] | 0,651865664 | 7,822387962 | Proinflammatory |
| IL-12p70 | -1.7[-20.5 : 21.5] | 0,874147596 | 10,48977116 | Proinflammatory |
| IL-15 | 0.5[-3.3 : 4.5] | 0,782944949 | 9,395339384 | Proinflammatory |
| TNF-A | -3.3[-7.8 : 1.4] | 0,161877828 | 1,94253394 | Proinflammatory |
| TNF-B | -0.9[-11.1 : 10.6] | 0,877020144 | 10,52424173 | Proinflammatory |
| IFN-G | -5.7[-16.5 : 6.6] | 0,346561948 | 4,50530533 | T derived |
| IL-2 | -7.3[-23.6 : 12.6] | 0,445273034 | 5,788549445 | T derived |
| IL-4 | 5.2[-12.0 : 25.8] | 0,574446978 | 7,467810708 | T derived |
| IL-5 | -2.2[-12.2 : 8.9] | 0,682271011 | 8,869523137 | T derived |
| IL-9 | 10.6[-9.9 : 35.7] | 0,336051979 | 4,368675721 | T derived |
| IL-10 | 7.9[-4.5 : 22.1] | 0,222892741 | 2,897605628 | T derived |
| IL-13 | 15.1[-3.0 : 36.6] | 0,105973757 | 1,377658846 | T derived |
| IL-16 | -10.8[-19.8 : -0.6] | 0,037805872 | 0,491476332 | T derived |
| IL-17A | -2.2[-11.1 : 7.5] | 0,638177012 | 8,296301158 | T derived |
| IL-17A/F | 2.7[-12.8 : 21.0] | 0,747042924 | 9,711558008 | T derived |
| IL-17B | 8.6[-6.1 : 25.7] | 0,266040242 | 3,458523149 | T derived |
| IL-17C | -10.3[-23.8 : 5.5] | 0,186795717 | 2,42834432 | T derived |
| IL-17D | -4.7[-18.8 : 11.9] | 0,559147268 | 7,26891448 | T derived |
| CCL2 | -3.7[-11.1 : 4.4] | 0,359893738 | 3,598937382 | Chemokine |
| CCL3 | 4.3[-3.9 : 13.2] | 0,312338778 | 3,123387775 | Chemokine |
| CCL4 | -6.7[-14.5 : 1.7] | 0,116166038 | 1,161660384 | Chemokine |
| CCL11 | -2.4[-9.0 : 4.6] | 0,491902476 | 4,919024756 | Chemokine |
| CCL13 | -0.2[-8.6 : 9.0] | 0,971490498 | 9,714904981 | Chemokine |
| CCL17 | 1.7[-10.3 : 15.4] | 0,792299174 | 7,922991742 | Chemokine |
| CCL22 | -3.8[-9.4 : 2.1] | 0,201419265 | 2,014192646 | Chemokine |
| CCL26 | -1.6[-12.5 : 10.6] | 0,786217448 | 7,862174479 | Chemokine |
| IL-8 | 0.1[-9.9 : 11.2] | 0,990299378 | 9,902993777 | Chemokine |
| CXCL10 | -6.2[-14.2 : 2.5] | 0,156114802 | 1,561148022 | Chemokine |
| bFGF | 7.8[-6.3 : 24.0] | 0,295428704 | 3,545144443 | Growth Factor |
| Flt-1 | -3.2[-8.1 : 2.1] | 0,229415098 | 2,75298118 | Growth Factor |
| GM-CSF | -6.7[-19.1 : 7.6] | 0,341459938 | 4,097519259 | Growth Factor |
| IL-7 | 1.9[-6.8 : 11.5] | 0,678144586 | 8,137735027 | Growth Factor |
| PlGF | 4.1[-0.2 : 8.7] | 0,063509645 | 0,762115743 | Growth Factor |
| sICAM-1 | -2.9[-6.8 : 1.2] | 0,160257381 | 1,923088577 | Growth Factor |
| sVCAM-1 | -4.0[-7.6 : -0.2] | 0,039259106 | 0,471109267 | Growth Factor |
| Tie-2 | -2.8[-7.2 : 1.9] | 0,23358416 | 2,803009922 | Growth Factor |
| TSLP | -15.7[-28.8 : -0.2] | 0,047422597 | 0,56907116 | Growth Factor |
| VEGF-A | 8.8[-5.1 : 24.7] | 0,227610861 | 2,731330336 | Growth Factor |
| VEGF-C | 2.6[-5.6 : 11.6] | 0,539102651 | 6,469231809 | Growth Factor |
| VEGF-D | -0.4[-8.9 : 8.9] | 0,922583081 | 11,07099697 | Growth Factor |

**Supplementary Table 6:** Model comparing the COC high group to the COC low group. Estimates are shown as percentage change and p values are shown both as crude and as corrected for multiple testing. Model is adjusted for age, current smoking, BMI, sample age, sample analysis date, and Danish administrative region of sampling.

| **Supplementary Table 7** | |  |  |  |  |  |  |  |  |  |  |
| --- | --- | --- | --- | --- | --- | --- | --- | --- | --- | --- | --- |
|  | COC low |  | COC high |  | IUD |  | POP |  | Menopausal |  |  |
| Assay | % estimate | corrected P | % estimate | corrected P | % estimate | corrected P | % estimate | corrected P | % estimate | corrected P | group |
| CRP | 282.2[225.3 : 349.2] | 1,18E-56 | 323.6[273.9 : 379.9] | 4,40E-106 | 1.4[-11.8 : 16.5] | 10,19103008 | -3.9[-24.5 : 22.3] | 8,972190935 | 15.2[2.1 : 30.0] | 0,260622895 | Proinflammatory |
| SAA | 62.0[41.6 : 85.3] | 2,67E-11 | 40.5[26.6 : 55.8] | 1,79E-09 | -11.5[-21.2 : -0.6] | 0,466581812 | -8.1[-24.8 : 12.3] | 4,907195901 | 21.2[9.6 : 34.0] | 0,002160616 | Proinflammatory |
| IL-1A | -5.6[-16.1 : 6.3] | 4,122176744 | -5.8[-14.1 : 3.4] | 2,493903914 | 1.2[-8.8 : 12.4] | 9,787185602 | -2.5[-18.7 : 16.9] | 9,398456677 | 4.0[-5.0 : 13.8] | 4,744467931 | Proinflammatory |
| IL-1B | 5.1[-14.9 : 29.8] | 7,749158059 | -3.5[-18.2 : 13.9] | 8,059615756 | -2.6[-19.2 : 17.3] | 9,339893495 | -5.5[-31.7 : 30.7] | 8,777150991 | 15.7[-1.5 : 35.9] | 0,899006172 | Proinflammatory |
| IL-1RA | 2.7[-5.0 : 10.9] | 6,063201261 | -3.8[-9.5 : 2.2] | 2,486421435 | 7.0[-0.1 : 14.5] | 0,621654772 | -4.8[-15.4 : 7.2] | 5,029586461 | 3.5[-2.4 : 9.8] | 3,016090805 | Proinflammatory |
| IL-3 | -2.3[-17.2 : 15.2] | 9,341276119 | -4.3[-15.9 : 9.0] | 6,097737121 | 8.4[-6.3 : 25.4] | 3,353326278 | 0.3[-22.3 : 29.5] | 11,77256498 | 10.4[-2.7 : 25.2] | 1,499682271 | Proinflammatory |
| IL-6 | -2.8[-14.2 : 10.2] | 7,914782195 | -4.9[-13.8 : 4.9] | 3,813089843 | -6.4[-16.1 : 4.6] | 2,918973108 | 2.2[-15.7 : 23.9] | 9,859673451 | 23.0[11.8 : 35.2] | 2,53E-04 | Proinflammatory |
| IL-12 | 7.4[0.0 : 15.4] | 0,598959499 | 2.3[-3.3 : 8.2] | 5,174222091 | 2.6[-3.7 : 9.3] | 5,131501973 | 12.4[0.7 : 25.5] | 0,450139775 | 8.6[2.9 : 14.7] | 0,035168027 | Proinflammatory |
| IL-12p70 | -7.4[-23.2 : 11.7] | 5,050930632 | -4.0[-17.1 : 11.2] | 7,023114116 | 15.6[-2.0 : 36.4] | 1,025422153 | 4.7[-21.5 : 39.7] | 9,029831921 | 10.7[-4.0 : 27.7] | 1,922152597 | Proinflammatory |
| IL-15 | 2.0[-1.7 : 5.8] | 3,482300824 | 2.9[0.0 : 5.9] | 0,562178316 | -1.6[-4.8 : 1.6] | 3,774828412 | -1.5[-6.9 : 4.2] | 7,081000302 | 0.4[-2.4 : 3.2] | 9,609693867 | Proinflammatory |
| TNF-A | 1.3[-3.3 : 6.2] | 6,992019431 | -2.1[-5.6 : 1.6] | 3,09348901 | 0.4[-3.6 : 4.6] | 10,16785794 | 1.6[-5.4 : 9.2] | 7,964163768 | 10.2[6.4 : 14.2] | 9,47E-07 | Proinflammatory |
| TNF-B | -4.8[-14.6 : 6.2] | 4,514724473 | -8.5[-15.9 : -0.3] | 0,510790784 | -3.2[-12.1 : 6.6] | 6,137597994 | 2.3[-13.5 : 21.0] | 9,479873147 | 7.9[-0.7 : 17.2] | 0,872428187 | Proinflammatory |
| IFN-G | 13.0[1.1 : 26.3] | 0,40406001 | 2.8[-5.7 : 12.2] | 6,90741063 | -8.2[-16.8 : 1.2] | 1,115893142 | -0.2[-15.9 : 18.4] | 12,78077713 | 10.1[1.2 : 19.8] | 0,324112084 | T derived |
| IL-2 | 14.9[-5.1 : 39.2] | 2,009355157 | 11.1[-4.4 : 29.1] | 2,199887219 | 35.3[14.3 : 60.2] | 0,00590277 | 3.4[-23.0 : 38.8] | 10,71233395 | 11.6[-3.5 : 29.1] | 1,806235239 | T derived |
| IL-4 | -1.1[-17.3 : 18.3] | 11,78363164 | 1.8[-11.5 : 17.2] | 10,3843095 | -0.1[-14.7 : 17.0] | 12,8726309 | 4.8[-20.4 : 38.0] | 9,594006975 | 9.5[-4.5 : 25.5] | 2,494683612 | T derived |
| IL-5 | 3.8[-5.9 : 14.5] | 5,958226118 | 3.1[-4.6 : 11.3] | 5,747644839 | -0.4[-8.6 : 8.6] | 12,15132527 | -5.5[-18.8 : 9.9] | 5,991694056 | -1.2[-8.3 : 6.5] | 9,861096697 | T derived |
| IL-9 | -12.9[-27.6 : 4.8] | 1,876440085 | -13.5[-25.2 : 0.0] | 0,643909968 | -3.9[-18.4 : 13.0] | 8,175690271 | -14.8[-35.8 : 13.2] | 3,499187654 | 19.4[3.7 : 37.4] | 0,179532418 | T derived |
| IL-10 | -4.7[-16.2 : 8.4] | 6,032680354 | -0.1[-9.7 : 10.5] | 12,75373383 | 6.2[-5.2 : 19.0] | 3,875192034 | 20.6[-1.0 : 47.1] | 0,824831717 | 9.2[-1.0 : 20.4] | 1,028863599 | T derived |
| IL-13 | -11.6[-24.6 : 3.6] | 1,663491954 | 3.3[-8.8 : 17.0] | 7,914873527 | 9.6[-4.7 : 26.1] | 2,595338772 | 5.0[-17.8 : 34.1] | 9,045811602 | -5.1[-15.9 : 7.1] | 5,134335874 | T derived |
| IL-16 | -2.0[-10.6 : 7.4] | 8,670992737 | -9.0[-15.3 : -2.2] | 0,135585543 | 8.0[-0.4 : 17.1] | 0,819570989 | -3.0[-15.8 : 11.7] | 8,758591259 | 6.3[-0.9 : 14.0] | 1,156250115 | T derived |
| IL-17A | 8.2[-1.6 : 18.8] | 1,341922773 | 5.6[-2.0 : 13.6] | 1,96439755 | 4.6[-3.7 : 13.7] | 3,747851755 | 9.1[-5.7 : 26.1] | 3,14383266 | 10.9[3.2 : 19.2] | 0,06035351 | T derived |
| IL-17A/F | 7.3[-8.1 : 25.3] | 4,84255901 | 5.2[-6.8 : 18.7] | 5,396915813 | -4.1[-16.3 : 9.9] | 7,134943289 | -18.1[-35.4 : 3.8] | 1,282234108 | -3.6[-14.3 : 8.5] | 7,057357901 | T derived |
| IL-17B | -18.9[-29.0 : -7.2] | 0,028653443 | -17.5[-25.7 : -8.4] | 0,00421815 | -10.4[-20.3 : 0.8] | 0,894236029 | -17.3[-32.7 : 1.7] | 0,932216564 | 14.6[3.5 : 26.9] | 0,112762181 | T derived |
| IL-17C | 2.8[-11.4 : 19.3] | 9,305933027 | -10.5[-20.3 : 0.6] | 0,831749051 | -8.2[-19.5 : 4.7] | 2,635147799 | -18.0[-34.8 : 3.2] | 1,172604376 | 1.6[-9.3 : 13.8] | 10,23037355 | T derived |
| IL-17D | 2.9[-10.0 : 17.7] | 8,781512966 | -2.0[-11.8 : 8.9] | 9,248707565 | -6.5[-16.9 : 5.3] | 3,459488983 | -1.5[-19.9 : 21.0] | 11,49651319 | 16.9[5.5 : 29.5] | 0,036146039 | T derived |
| CCL2 | 7.0[0.1 : 14.4] | 0,460629055 | 1.4[-3.6 : 6.8] | 5,840115892 | -0.2[-5.7 : 5.7] | 9,573098565 | 1.3[-8.4 : 12.1] | 8,039303883 | 16.2[10.6 : 22.1] | 2,76E-08 | Chemokine |
| CCL3 | 0.7[-6.1 : 8.0] | 8,399083969 | 3.4[-2.0 : 9.1] | 2,193886418 | 0.7[-5.1 : 6.9] | 8,070605887 | 11.9[0.8 : 24.4] | 0,356396228 | 10.2[4.7 : 16.0] | 0,002141021 | Chemokine |
| CCL4 | -4.3[-11.3 : 3.3] | 2,59308951 | -8.2[-13.4 : -2.6] | 0,042980384 | -1.4[-7.6 : 5.3] | 6,810993805 | -4.7[-15.1 : 7.0] | 4,19502593 | 9.1[3.1 : 15.4] | 0,026005801 | Chemokine |
| CCL11 | -7.6[-13.0 : -1.8] | 0,115007982 | -11.7[-15.7 : -7.5] | 1,81E-06 | -4.1[-9.0 : 1.0] | 1,151447258 | 4.7[-4.5 : 14.8] | 3,295685651 | 7.7[3.0 : 12.7] | 0,012708166 | Chemokine |
| CCL13 | -11.5[-18.1 : -4.3] | 0,021506331 | -12.6[-17.6 : -7.2] | 1,04E-04 | 1.5[-5.0 : 8.5] | 6,586636518 | -10.1[-20.1 : 1.1] | 0,765808691 | 5.1[-0.8 : 11.3] | 0,929552705 | Chemokine |
| CCL17 | -22.5[-30.5 : -13.6] | 4,62E-05 | -19.8[-26.2 : -12.8] | 2,38E-06 | 1.2[-7.7 : 11.1] | 7,960793663 | -5.2[-19.6 : 11.8] | 5,251932911 | 20.3[11.0 : 30.3] | 7,30E-05 | Chemokine |
| CCL22 | 5.9[0.5 : 11.5] | 0,309994449 | -0.2[-4.1 : 3.8] | 9,214236082 | -0.6[-4.9 : 3.8] | 7,752174719 | -1.0[-8.5 : 7.0] | 7,958333304 | 16.3[11.9 : 20.8] | 1,36E-13 | Chemokine |
| CCL26 | -4.2[-14.2 : 6.9] | 4,42643253 | -7.2[-14.7 : 1.0] | 0,822089005 | -0.8[-9.7 : 8.9] | 8,687319339 | -1.3[-16.4 : 16.5] | 8,769933942 | -3.5[-11.0 : 4.7] | 3,94824527 | Chemokine |
| IL-8 | 5.9[-5.4 : 18.6] | 3,213843565 | 9.9[0.5 : 20.1] | 0,378598689 | 5.5[-4.5 : 16.6] | 2,9368047 | -7.4[-22.3 : 10.2] | 3,846040714 | 5.0[-3.6 : 14.5] | 2,637661472 | Chemokine |
| CXCL10 | -2.1[-9.8 : 6.2] | 6,023156654 | -6.4[-12.1 : -0.4] | 0,375684965 | 2.7[-4.1 : 10.1] | 4,442913604 | 1.4[-10.3 : 14.7] | 8,238122738 | 12.3[5.8 : 19.3] | 0,001562984 | Chemokine |
| bFGF | 10.8[-2.4 : 25.7] | 1,342762244 | 18.6[7.4 : 31.0] | 0,009083914 | 5.6[-5.5 : 18.1] | 4,027966293 | -8.7[-24.9 : 10.9] | 4,286593814 | 6.1[-3.6 : 16.8] | 2,746012681 | Growth Factor |
| Flt-1 | -14.7[-18.6 : -10.6] | 3,16E-10 | -14.4[-17.4 : -11.2] | 1,54E-15 | 1.2[-2.9 : 5.4] | 6,913034715 | 5.1[-2.2 : 12.9] | 2,091168765 | -0.7[-4.2 : 2.9] | 8,238662428 | Growth Factor |
| GM-CSF | -7.5[-19.8 : 6.7] | 3,438601001 | -4.5[-14.6 : 6.8] | 5,040748217 | -3.9[-15.3 : 9.0] | 6,465286924 | 3.5[-17.0 : 28.9] | 9,145971799 | 9.0[-2.2 : 21.5] | 1,437820899 | Growth Factor |
| IL-7 | 9.4[1.5 : 18.1] | 0,235303571 | 13.0[6.5 : 19.9] | 6,81E-04 | -4.1[-10.3 : 2.6] | 2,705651639 | -5.3[-15.7 : 6.4] | 4,334719037 | -0.2[-5.8 : 5.7] | 11,26556857 | Growth Factor |
| PlGF | 0.6[-3.2 : 4.4] | 9,267149871 | 3.4[0.4 : 6.5] | 0,336052242 | 0.5[-2.7 : 3.9] | 8,968979083 | -1.8[-7.3 : 4.1] | 6,463506749 | 8.8[5.8 : 12.0] | 9,18E-08 | Growth Factor |
| sICAM-1 | 5.8[0.5 : 11.4] | 0,370810294 | 2.4[-1.6 : 6.5] | 2,976707918 | 0.0[-4.3 : 4.6] | 11,93695956 | 1.2[-6.3 : 9.3] | 9,099918117 | 14.0[9.7 : 18.5] | 3,66E-10 | Growth Factor |
| sVCAM-1 | -6.3[-10.1 : -2.4] | 0,020651847 | -11.9[-14.6 : -9.0] | 6,43E-14 | 1.9[-1.6 : 5.6] | 3,582161055 | 0.7[-5.2 : 7.1] | 9,730457479 | 6.7[3.5 : 10.0] | 3,98E-04 | Growth Factor |
| Tie-2 | -1.9[-5.8 : 2.1] | 4,117256593 | -5.6[-8.5 : -2.6] | 0,003636344 | 0.5[-3.0 : 4.1] | 9,511413297 | 6.3[-0.1 : 13.0] | 0,636072596 | 3.9[0.7 : 7.1] | 0,177430609 | Growth Factor |
| TSLP | 8.3[-7.1 : 26.2] | 3,709267119 | -5.1[-15.8 : 7.0] | 4,714679675 | -16.5[-27.1 : -4.5] | 0,105506262 | 0.8[-20.3 : 27.6] | 11,35019126 | 14.6[2.0 : 28.9] | 0,261744824 | Growth Factor |
| VEGF-A | 33.3[16.3 : 52.9] | 4,40E-04 | 37.7[23.8 : 53.3] | 5,92E-08 | -1.9[-13.0 : 10.7] | 9,116690543 | -5.3[-23.2 : 16.8] | 7,334161262 | 8.5[-2.2 : 20.4] | 1,482697723 | Growth Factor |
| VEGF-C | 12.5[4.1 : 21.6] | 0,03365192 | 14.1[7.4 : 21.3] | 2,39E-04 | 0.8[-5.9 : 7.9] | 9,919952555 | 5.5[-6.4 : 18.8] | 4,5677751 | 5.9[-0.2 : 12.3] | 0,678775241 | Growth Factor |
| VEGF-D | 41.6[29.5 : 54.8] | 3,09E-13 | 24.5[16.1 : 33.5] | 1,03E-08 | -4.9[-12.1 : 2.8] | 2,483126528 | -5.5[-17.6 : 8.4] | 5,050279262 | 2.1[-4.6 : 9.2] | 6,650606812 | Growth Factor |

**Supplementary Table 7:** Model including duration of use as a covariate. Estimates are shown as percentage change and p values are corrected for multiple testing. Model is adjusted for age, current smoking, BMI, duration of use, sample age, sample analysis date, and Danish administrative region of sampling.

| **Supplementary Table 8** | |  |  |  |  |  |  |  |  |  |  |
| --- | --- | --- | --- | --- | --- | --- | --- | --- | --- | --- | --- |
|  | COC low |  | COC high |  | IUD |  | POP |  | Menopausal |  |  |
| Assay | % estimate | corrected P | % estimate | corrected P | % estimate | corrected P | % estimate | corrected P | % estimate | corrected P | group |
| CRP | 0.5[-0.5 : 1.4] | 3,921357316 | 0.4[-1.0 : 1.8] | 7,144349679 | -3.3[-9.0 : 2.8] | 3,402356454 | 34.9[-46.6 : 241.2] | 6,219977628 | 13.5[12.2 : 14.8] | 10,73861706 | Proinflammatory |
| SAA | 0.0[-0.8 : 0.8] | 10,86272651 | -0.1[-1.2 : 1.1] | 11,00185193 | -3.0[-7.5 : 1.8] | 2,600377984 | 108.2[7.3 : 303.8] | 0,370787458 | 5.7[4.7 : 6.7] | 8,598524776 | Proinflammatory |
| IL-1A | -0.1[-0.8 : 0.5] | 8,732852014 | 0.6[-0.4 : 1.7] | 2,685004613 | -2.5[-7.3 : 2.6] | 3,938816757 | 46.9[-27.7 : 198.7] | 3,353734128 | 0.7[-0.2 : 1.6] | 4,485719442 | Proinflammatory |
| IL-1B | 0.5[-0.7 : 1.6] | 5,203886955 | -0.3[-2.2 : 1.6] | 9,141721159 | -4.9[-13.4 : 4.4] | 3,49661026 | 171.1[-2.5 : 653.6] | 0,666712667 | 0.8[-0.8 : 2.4] | 0,007656924 | Proinflammatory |
| IL-1RA | 0.0[-0.4 : 0.5] | 9,788948369 | -0.1[-0.9 : 0.7] | 9,60355613 | 2.5[-0.8 : 5.9] | 1,595433194 | 61.2[2.0 : 154.9] | 0,496299376 | 3.6[3.0 : 4.2] | 5,43E-13 | Proinflammatory |
| IL-3 | 0.8[-0.1 : 1.8] | 1,060222509 | -0.6[-2.0 : 0.9] | 5,241365883 | 0.5[-6.8 : 8.3] | 10,81855074 | -57.0[-84.5 : 19.6] | 1,242945929 | -0.2[-1.5 : 1.0] | 7,427601128 | Proinflammatory |
| IL-6 | 0.2[-0.8 : 1.1] | 8,670075521 | 0.2[-0.9 : 1.2] | 8,961427765 | -2.6[-7.8 : 2.8] | 4,018329763 | -2.9[-45.2 : 72.1] | 11,0061839 | 7.5[6.5 : 8.4] | 1,157846012 | Proinflammatory |
| IL-12 | -0.3[-0.8 : 0.3] | 3,897473979 | -1.3[-1.8 : -0.7] | 7,87E-05 | -3.8[-7.2 : -0.4] | 0,373799675 | -18.9[-49.0 : 29.0] | 4,403192432 | 2.4[1.8 : 3.0] | 3,57130829 | Proinflammatory |
| IL-12p70 | -0.8[-2.0 : 0.4] | 2,166237532 | 0.0[-1.7 : 1.7] | 11,88453408 | -3.1[-9.7 : 4.0] | 4,546606227 | -25.8[-74.1 : 112.3] | 6,828863821 | -0.1[-1.5 : 1.3] | 11,80412396 | Proinflammatory |
| IL-15 | -0.2[-0.5 : 0.1] | 1,332799818 | 0.3[0.0 : 0.6] | 0,437933033 | -1.3[-3.5 : 1.0] | 3,307872606 | 20.7[2.0 : 42.9] | 0,354920366 | -0.2[-0.5 : 0.0] | 3,150187723 | Proinflammatory |
| TNF-A | -0.1[-0.3 : 0.2] | 7,920376192 | -0.2[-0.6 : 0.2] | 3,617657349 | -1.1[-3.0 : 0.9] | 3,321154624 | 1.6[-23.3 : 34.6] | 10,92162298 | 1.4[1.1 : 1.8] | 1,28277802 | Proinflammatory |
| TNF-B | -0.3[-1.0 : 0.3] | 3,996988337 | -1.1[-1.9 : -0.2] | 0,199718428 | -3.2[-7.3 : 1.1] | 1,694306487 | 19.5[-39.5 : 136.1] | 7,198364771 | 0.7[-0.2 : 1.6] | 5,614354393 | Proinflammatory |
| IFN-G | 0.9[0.2 : 1.6] | 0,180575942 | 0.2[-0.8 : 1.2] | 9,196226422 | 1.4[-3.1 : 6.1] | 7,171599663 | 28.7[-26.1 : 123.8] | 4,723907835 | 0.9[0.0 : 1.7] | 8,468973869 | T derived |
| IL-2 | -0.7[-1.8 : 0.4] | 2,872945016 | 1.8[0.3 : 3.4] | 0,252920246 | -1.0[-8.1 : 6.7] | 10,23975142 | 87.4[-42.3 : 508.1] | 3,738250308 | 0.0[-1.4 : 1.5] | 3,289664363 | T derived |
| IL-4 | 0.1[-0.9 : 1.0] | 11,89073872 | -0.4[-1.8 : 1.0] | 7,143883818 | -3.8[-10.4 : 3.1] | 3,543673645 | -13.0[-61.6 : 97.4] | 9,536234053 | 1.7[0.2 : 3.1] | 2,631206787 | T derived |
| IL-5 | 0.4[-0.2 : 1.0] | 2,245356579 | 0.7[-0.2 : 1.6] | 1,367717732 | -7.0[-10.7 : -3.2] | 0,006316278 | -0.9[-58.5 : 137.0] | 12,79038385 | 0.2[-0.5 : 0.9] | 1,244895421 | T derived |
| IL-9 | -0.1[-1.2 : 1.0] | 11,40999323 | -1.1[-2.7 : 0.5] | 2,447909487 | 3.3[-4.8 : 12.0] | 5,684140727 | -11.9[-69.2 : 152.2] | 10,52525041 | -2.3[-3.6 : -0.9] | 10,78772694 | T derived |
| IL-10 | 0.6[-0.2 : 1.4] | 1,82084634 | -0.9[-1.9 : 0.0] | 0,628500494 | -6.6[-11.0 : -2.1] | 0,066176378 | 30.2[-30.5 : 144.1] | 5,212773129 | -1.1[-2.1 : -0.1] | 1,771067113 | T derived |
| IL-13 | -0.2[-1.2 : 0.8] | 8,566079024 | 0.5[-0.8 : 1.8] | 6,253574346 | -0.8[-6.8 : 5.6] | 10,42867526 | -68.8[-89.5 : -7.4] | 0,473468198 | 0.3[-0.9 : 1.4] | 0,58748895 | T derived |
| IL-16 | -0.8[-1.4 : -0.1] | 0,255102387 | -0.5[-1.3 : 0.3] | 2,690264899 | -2.3[-6.0 : 1.6] | 3,264150729 | 17.4[-42.5 : 139.9] | 8,472170811 | 0.5[-0.2 : 1.2] | 1,77E-18 | T derived |
| IL-17A | -0.3[-0.8 : 0.2] | 3,370157177 | -1.1[-1.9 : -0.4] | 0,032626658 | -4.6[-8.1 : -1.0] | 0,166387971 | -2.1[-54.2 : 109.1] | 12,41032659 | 1.9[1.1 : 2.7] | 8,306045196 | T derived |
| IL-17A/F | 0.3[-0.5 : 1.1] | 5,916230553 | -1.1[-2.4 : 0.3] | 1,484579205 | -4.5[-10.6 : 2.1] | 2,337387796 | 27.5[-43.9 : 190.1] | 7,205871152 | -1.0[-2.2 : 0.2] | 12,17477952 | T derived |
| IL-17B | 0.0[-0.7 : 0.7] | 12,4642625 | -0.4[-1.6 : 0.9] | 7,447662228 | -8.1[-13.3 : -2.5] | 0,063613368 | -45.1[-77.3 : 32.4] | 2,298867755 | -1.4[-2.4 : -0.4] | 12,66268844 | T derived |
| IL-17C | 0.3[-0.4 : 1.1] | 4,749131827 | -0.3[-1.6 : 1.1] | 9,025624302 | 0.0[-6.3 : 6.8] | 12,89297834 | -4.1[-58.4 : 120.9] | 11,95338038 | 0.5[-0.7 : 1.6] | 4,327644036 | T derived |
| IL-17D | 0.9[-0.1 : 1.8] | 1,047200302 | 1.1[-0.1 : 2.4] | 1,000654029 | -0.3[-5.8 : 5.5] | 11,86993569 | 37.1[-38.9 : 207.7] | 5,677729712 | -1.7[-2.5 : -0.9] | 0,36161136 | T derived |
| CCL2 | 0.1[-0.2 : 0.4] | 6,178702481 | 0.1[-0.6 : 0.8] | 7,326902452 | -1.1[-3.3 : 1.1] | 3,124469446 | 26.3[-15.2 : 88.1] | 2,437081714 | -0.1[-0.6 : 0.4] | 2,135821693 | Chemokine |
| CCL3 | 0.1[-0.4 : 0.5] | 7,762253524 | 0.2[-0.4 : 0.7] | 4,986991792 | -1.3[-3.9 : 1.3] | 3,146642203 | -15.8[-50.0 : 41.9] | 5,091201294 | 1.4[0.9 : 1.8] | 3,615292586 | Chemokine |
| CCL4 | -0.1[-0.6 : 0.3] | 5,126535371 | 0.4[-0.3 : 1.1] | 2,870210202 | -0.3[-3.2 : 2.6] | 8,300536404 | 16.3[-24.5 : 79.2] | 4,832885482 | 0.6[0.1 : 1.2] | 1,720860641 | Chemokine |
| CCL11 | 0.0[-0.3 : 0.4] | 8,256042643 | 0.7[0.1 : 1.2] | 0,135827756 | 0.5[-1.9 : 2.9] | 6,782557071 | 39.3[-6.0 : 106.5] | 0,96448229 | -1.1[-1.5 : -0.6] | 0,735031114 | Chemokine |
| CCL13 | 0.0[-0.4 : 0.5] | 8,623466547 | 0.9[0.2 : 1.6] | 0,151889852 | -0.6[-3.4 : 2.3] | 6,853523804 | 15.3[-35.6 : 106.4] | 6,249472801 | 1.2[0.6 : 1.8] | 0,197184275 | Chemokine |
| CCL17 | -0.2[-0.8 : 0.4] | 4,578525737 | -0.3[-1.3 : 0.7] | 5,387756823 | 1.9[-1.9 : 5.8] | 3,368537672 | 44.7[-28.2 : 191.7] | 2,931777043 | -0.2[-1.0 : 0.6] | 0,44078209 | Chemokine |
| CCL22 | 0.1[-0.2 : 0.4] | 4,256800682 | -0.7[-1.2 : -0.2] | 0,076412393 | -0.6[-2.4 : 1.1] | 4,745852352 | 29.6[-9.8 : 86.4] | 1,559332272 | 1.6[1.2 : 2.0] | 4,253777204 | Chemokine |
| CCL26 | 0.0[-0.7 : 0.7] | 9,714060854 | 0.3[-0.6 : 1.2] | 5,608889786 | -4.0[-7.8 : 0.0] | 0,473714485 | 22.1[-33.1 : 122.9] | 5,057523119 | 0.9[0.1 : 1.8] | 1,715683249 | Chemokine |
| IL-8 | -0.3[-0.8 : 0.3] | 3,042750433 | 0.5[-0.3 : 1.4] | 2,374340033 | 2.1[-2.4 : 6.8] | 3,617071308 | 15.9[-28.8 : 88.4] | 5,443369482 | 0.0[-0.8 : 0.9] | 1,01E-39 | Chemokine |
| CXCL10 | 0.0[-0.4 : 0.4] | 8,78987701 | 0.2[-0.5 : 1.0] | 5,610646129 | -0.6[-3.3 : 2.2] | 6,835477792 | -0.4[-39.6 : 64.3] | 9,872025952 | 1.6[1.0 : 2.2] | 4,769613939 | Chemokine |
| bFGF | 0.1[-0.6 : 0.9] | 8,983552493 | 0.3[-0.9 : 1.4] | 7,694095139 | -4.1[-9.5 : 1.7] | 1,940079341 | -7.9[-53.5 : 82.6] | 9,713433377 | 0.4[-0.6 : 1.3] | 0,004211207 | Growth Factor |
| Flt-1 | 0.0[-0.3 : 0.2] | 9,191889288 | -0.1[-0.6 : 0.3] | 6,665227004 | -3.3[-6.0 : -0.5] | 0,264124196 | -11.4[-30.0 : 12.2] | 3,683480115 | 0.5[0.2 : 0.8] | 7,810121575 | Growth Factor |
| GM-CSF | -0.4[-1.2 : 0.4] | 3,868046022 | -0.7[-1.8 : 0.4] | 2,394430604 | -0.1[-5.7 : 5.8] | 11,6022863 | 60.8[-15.2 : 205.3] | 1,698746374 | 1.2[0.1 : 2.3] | 2,810265171 | Growth Factor |
| IL-7 | -0.1[-0.7 : 0.5] | 9,047382716 | -0.2[-0.8 : 0.5] | 7,336613469 | -0.9[-4.2 : 2.5] | 7,302689451 | -26.2[-52.8 : 15.4] | 2,12112249 | 0.9[0.4 : 1.5] | 0,001827885 | Growth Factor |
| PlGF | 0.1[-0.1 : 0.3] | 5,341325767 | 0.3[0.0 : 0.7] | 0,826666512 | -2.1[-4.0 : -0.2] | 0,336631811 | -3.8[-21.8 : 18.3] | 8,453680622 | 0.7[0.5 : 1.0] | 2,595927619 | Growth Factor |
| sICAM-1 | 0.1[-0.2 : 0.3] | 6,527115734 | -0.3[-0.6 : 0.1] | 1,338283307 | 0.3[-1.1 : 1.7] | 8,324184057 | 1.7[-16.8 : 24.4] | 10,36477584 | 1.0[0.7 : 1.3] | 0,083670922 | Growth Factor |
| sVCAM-1 | 0.1[-0.1 : 0.3] | 5,806694368 | -0.2[-0.5 : 0.1] | 3,592193767 | 0.7[-0.6 : 2.0] | 3,325252893 | -10.2[-26.1 : 9.1] | 3,232864097 | 0.5[0.3 : 0.8] | 6,723558711 | Growth Factor |
| Tie-2 | 0.0[-0.3 : 0.2] | 9,698452567 | 0.1[-0.3 : 0.5] | 7,341012257 | -1.2[-3.6 : 1.2] | 3,803077041 | -8.7[-22.7 : 7.8] | 3,279735963 | -0.1[-0.4 : 0.2] | 9,777672797 | Growth Factor |
| TSLP | 0.2[-0.5 : 1.0] | 6,541344117 | 1.3[-0.1 : 2.7] | 0,906655892 | -2.8[-10.8 : 5.8] | 6,115434098 | -45.9[-80.1 : 46.7] | 2,656808116 | 2.5[1.5 : 3.6] | 11,45640294 | Growth Factor |
| VEGF | 0.4[-0.3 : 1.2] | 3,347313071 | 0.9[-0.2 : 2.0] | 1,154233536 | -4.7[-11.3 : 2.4] | 2,293137081 | -8.2[-54.4 : 84.4] | 9,653906474 | 1.0[0.0 : 2.0] | 0,068396547 | Growth Factor |
| VEGF-C | 0.2[-0.3 : 0.7] | 4,00806307 | 0.0[-0.7 : 0.6] | 11,73461763 | -1.4[-4.8 : 2.2] | 5,402944813 | 3.5[-30.0 : 53.0] | 10,30495706 | 0.4[-0.2 : 1.0] | 1,443429594 | Growth Factor |
| VEGF-D | 0.5[0.0 : 1.0] | 0,397458038 | 0.7[0.0 : 1.4] | 0,809582269 | -2.6[-6.5 : 1.5] | 2,573794446 | -16.4[-45.2 : 27.7] | 4,780393077 | -1.9[-2.6 : -1.2] | 0,004354157 | Growth Factor |

**Supplementary Table 8:** Model using duration of use as the exposure. Estimates are shown as percentage change for each month of consecutive contraceptive use and p values are corrected for multiple testing. Model is adjusted for age, current smoking, BMI, sample age, sample analysis date, and Danish administrative region of sampling.

| **Supplementary Table 9** | |  |  |  |  |  |  |  |  |  |  |
| --- | --- | --- | --- | --- | --- | --- | --- | --- | --- | --- | --- |
|  | COC low |  | COC high |  | IUD |  | POP |  | Menopausal |  |  |
| Assay | % estimate | corrected P | % estimate | corrected P | % estimate | corrected P | % estimate | corrected P | % estimate | corrected P | group |
| CRP | 289.8[233.7 : 355.4] | 1,74E-62 | 333.9[287.4 : 385.9] | 1,01E-130 | 5.3[-5.7 : 17.7] | 4,304231425 | -1.9[-22.6 : 24.3] | 10,4912022 | 15.0[1.9 : 29.7] | 0,28130714 | Proinflammatory |
| SAA | 62.9[43.1 : 85.4] | 2,10E-12 | 41.5[28.7 : 55.4] | 7,64E-12 | -10.5[-18.4 : -1.9] | 0,221933266 | -7.6[-24.1 : 12.6] | 5,210731389 | 21.1[9.5 : 33.9] | 0,002229741 | Proinflammatory |
| IL-1A | -5.7[-15.9 : 5.6] | 3,728631341 | -6.0[-13.5 : 2.2] | 1,793322181 | 0.9[-7.1 : 9.6] | 9,98913797 | -2.7[-18.6 : 16.4] | 9,172293366 | 4.0[-4.9 : 13.8] | 4,717676527 | Proinflammatory |
| IL-1B | 6.8[-12.9 : 30.9] | 6,337183842 | -1.6[-15.3 : 14.3] | 9,988746425 | 0.5[-13.4 : 16.6] | 11,39855455 | -4.0[-30.2 : 32.2] | 9,653707319 | 15.5[-1.6 : 35.7] | 0,933182994 | Proinflammatory |
| IL-1RA | 1.9[-5.4 : 9.8] | 7,440949051 | -4.7[-9.8 : 0.7] | 1,013833834 | 5.4[-0.1 : 11.3] | 0,675676065 | -5.5[-15.9 : 6.2] | 4,094743583 | 3.6[-2.3 : 9.9] | 2,898975066 | Proinflammatory |
| IL-3 | -0.9[-15.5 : 16.2] | 10,96293654 | -2.5[-13.3 : 9.6] | 8,079291217 | 11.6[-0.6 : 25.4] | 0,763169621 | 1.9[-20.8 : 31.0] | 10,61891412 | 10.2[-2.8 : 25.0] | 1,562395671 | Proinflammatory |
| IL-6 | -2.5[-13.5 : 10.0] | 8,223700046 | -4.5[-12.6 : 4.4] | 3,716332071 | -5.8[-13.7 : 2.9] | 2,228670153 | 2.6[-15.1 : 24.0] | 9,502633805 | 22.9[11.8 : 35.2] | 2,57E-04 | Proinflammatory |
| IL-12 | 5.9[-1.2 : 13.4] | 1,245778648 | 0.5[-4.5 : 5.7] | 10,25303638 | -0.3[-5.2 : 4.9] | 10,91339437 | 10.7[-0.6 : 23.4] | 0,782313829 | 8.8[3.0 : 14.8] | 0,029992882 | Proinflammatory |
| IL-12p70 | -11.1[-25.8 : 6.4] | 2,388220661 | -8.8[-20.1 : 4.2] | 2,113449447 | 6.5[-6.6 : 21.5] | 4,151478745 | 0.3[-24.4 : 33.2] | 11,7711849 | 11.2[-3.6 : 28.2] | 1,737131151 | Proinflammatory |
| IL-15 | 0.7[-2.8 : 4.3] | 8,333566094 | 1.3[-1.2 : 4.0] | 3,730273014 | -4.1[-6.5 : -1.6] | 0,018323472 | -2.8[-8.0 : 2.7] | 3,719403555 | 0.5[-2.3 : 3.3] | 8,861048023 | Proinflammatory |
| TNF-A | 0.0[-4.4 : 4.6] | 11,86494444 | -3.7[-6.8 : -0.4] | 0,317309971 | -2.2[-5.4 : 1.1] | 2,210601357 | 0.2[-6.6 : 7.6] | 11,42651501 | 10.4[6.5 : 14.4] | 6,37E-07 | Proinflammatory |
| TNF-B | -4.9[-14.4 : 5.6] | 4,145572317 | -8.6[-15.4 : -1.3] | 0,270632141 | -3.4[-10.5 : 4.3] | 4,479392062 | 2.2[-13.4 : 20.5] | 9,587475442 | 7.9[-0.7 : 17.2] | 0,86481001 | Proinflammatory |
| IFN-G | 16.2[4.4 : 29.3] | 0,076718758 | 6.4[-1.6 : 15.2] | 1,56584251 | -3.0[-10.3 : 4.9] | 5,825094833 | 2.8[-13.1 : 21.6] | 9,742320847 | 9.9[1.0 : 19.5] | 0,378608562 | T derived |
| IL-2 | 8.0[-10.2 : 29.9] | 5,344176153 | 3.0[-10.1 : 18.0] | 8,760269165 | 19.8[4.7 : 37.0] | 0,112831906 | -3.0[-27.4 : 29.6] | 10,86289658 | 12.3[-2.9 : 29.9] | 1,551261644 | T derived |
| IL-4 | -1.1[-16.8 : 17.5] | 11,68420031 | 1.8[-10.4 : 15.6] | 10,20455295 | -0.2[-12.0 : 13.2] | 12,69603674 | 4.8[-20.1 : 37.3] | 9,571108891 | 9.5[-4.4 : 25.5] | 2,488042768 | T derived |
| IL-5 | 5.1[-4.3 : 15.6] | 3,869427529 | 4.7[-2.3 : 12.3] | 2,500986145 | 2.3[-4.6 : 9.6] | 6,850699916 | -4.2[-17.4 : 11.1] | 7,393949827 | -1.3[-8.4 : 6.4] | 9,549873824 | T derived |
| IL-9 | -8.9[-23.8 : 8.8] | 3,934603598 | -8.7[-19.9 : 4.1] | 2,284061802 | 4.8[-8.0 : 19.3] | 6,259133461 | -10.8[-32.5 : 17.9] | 5,49138586 | 18.9[3.3 : 36.8] | 0,20996084 | T derived |
| IL-10 | -7.5[-18.3 : 4.7] | 2,813269255 | -3.8[-12.2 : 5.4] | 5,342303673 | 0.1[-8.6 : 9.6] | 12,83790699 | 16.9[-3.8 : 42.1] | 1,50581248 | 9.5[-0.7 : 20.7] | 0,908259656 | T derived |
| IL-13 | -12.9[-25.3 : 1.5] | 0,98777029 | 1.4[-9.4 : 13.5] | 10,52362694 | 6.4[-4.9 : 18.9] | 3,612799337 | 3.4[-18.7 : 31.5] | 10,23811961 | -5.0[-15.8 : 7.2] | 5,298334156 | T derived |
| IL-16 | -3.5[-11.7 : 5.4] | 5,609227032 | -10.7[-16.3 : -4.7] | 0,008888846 | 4.8[-1.8 : 11.8] | 2,048737426 | -4.5[-16.9 : 9.7] | 6,674293865 | 6.4[-0.8 : 14.1] | 1,060152936 | T derived |
| IL-17A | 5.9[-3.3 : 15.9] | 2,841450179 | 2.8[-3.8 : 9.9] | 5,416307952 | 0.3[-6.2 : 7.2] | 12,15043889 | 6.6[-7.5 : 23.0] | 4,897419035 | 11.1[3.4 : 19.4] | 0,05086493 | T derived |
| IL-17A/F | 7.7[-7.2 : 25.0] | 4,266087923 | 5.7[-5.3 : 17.9] | 4,199903164 | -3.3[-13.3 : 7.8] | 7,024951578 | -17.8[-34.9 : 3.8] | 1,297739055 | -3.6[-14.3 : 8.4] | 7,001242622 | T derived |
| IL-17B | -18.4[-28.2 : -7.1] | 0,026535269 | -16.9[-24.4 : -8.6] | 0,001789695 | -9.3[-17.4 : -0.3] | 0,558653399 | -16.7[-32.0 : 2.0] | 1,000219158 | 14.6[3.5 : 26.9] | 0,116056647 | T derived |
| IL-17C | 3.0[-10.8 : 18.9] | 8,907181654 | -10.2[-19.2 : -0.2] | 0,588728044 | -7.8[-17.0 : 2.4] | 1,661325048 | -17.8[-34.4 : 3.0] | 1,145960695 | 1.6[-9.3 : 13.8] | 10,26124678 | T derived |
| IL-17D | 5.1[-7.6 : 19.6] | 5,803739845 | 0.7[-8.4 : 10.7] | 11,52005893 | -2.4[-11.2 : 7.2] | 7,936617055 | 0.7[-17.8 : 23.4] | 12,28355174 | 16.7[5.3 : 29.2] | 0,040827941 | T derived |
| CCL2 | 6.6[-0.1 : 13.6] | 0,529841633 | 0.9[-3.7 : 5.7] | 7,055590033 | -1.0[-5.4 : 3.6] | 6,615518001 | 0.8[-8.7 : 11.4] | 8,709493456 | 16.3[10.7 : 22.1] | 2,43E-08 | Chemokine |
| CCL3 | -0.2[-6.7 : 6.7] | 9,547476283 | 2.3[-2.5 : 7.4] | 3,601087553 | -1.0[-5.6 : 3.8] | 6,794458685 | 10.9[0.0 : 23.0] | 0,497975963 | 10.3[4.8 : 16.1] | 0,001865695 | Chemokine |
| CCL4 | -4.8[-11.5 : 2.5] | 1,9418731 | -8.7[-13.4 : -3.7] | 0,00805652 | -2.2[-7.2 : 3.0] | 3,912104444 | -5.1[-15.3 : 6.3] | 3,660383021 | 9.1[3.1 : 15.5] | 0,024541507 | Chemokine |
| CCL11 | -7.0[-12.3 : -1.4] | 0,150483485 | -11.1[-14.8 : -7.2] | 5,75E-07 | -3.0[-7.0 : 1.1] | 1,460227293 | 5.3[-3.8 : 15.3] | 2,632728175 | 7.6[2.9 : 12.6] | 0,013699363 | Chemokine |
| CCL13 | -12.0[-18.3 : -5.1] | 0,008391628 | -13.2[-17.7 : -8.3] | 3,36E-06 | 0.4[-4.8 : 5.8] | 8,861321889 | -10.6[-20.4 : 0.4] | 0,573446352 | 5.1[-0.8 : 11.3] | 0,891695839 | Chemokine |
| CCL17 | -22.7[-30.4 : -14.1] | 1,58E-05 | -20.0[-25.8 : -13.7] | 8,63E-08 | 0.8[-6.4 : 8.5] | 8,32883309 | -5.4[-19.5 : 11.2] | 5,005183048 | 20.3[11.0 : 30.4] | 7,02E-05 | Chemokine |
| CCL22 | 5.7[0.6 : 11.1] | 0,290768214 | -0.4[-3.9 : 3.3] | 8,380298747 | -0.9[-4.3 : 2.6] | 6,057380389 | -1.2[-8.5 : 6.7] | 7,630775585 | 16.3[11.9 : 20.8] | 1,23E-13 | Chemokine |
| CCL26 | -4.4[-14.0 : 6.2] | 4,025507706 | -7.4[-14.2 : -0.1] | 0,478736954 | -1.2[-8.3 : 6.5] | 7,533949343 | -1.5[-16.3 : 15.9] | 8,545415204 | -3.4[-11.0 : 4.7] | 3,971754573 | Chemokine |
| IL-8 | 3.6[-7.1 : 15.6] | 5,238936122 | 6.9[-1.3 : 15.9] | 1,016901873 | 1.0[-6.7 : 9.4] | 7,988978034 | -9.5[-23.8 : 7.4] | 2,528488086 | 5.3[-3.4 : 14.7] | 2,441684915 | Chemokine |
| CXCL10 | -3.2[-10.5 : 4.7] | 4,209605094 | -7.6[-12.6 : -2.2] | 0,065068951 | 0.7[-4.7 : 6.4] | 8,03633875 | 0.3[-11.1 : 13.2] | 9,572780518 | 12.4[5.9 : 19.4] | 0,001360196 | Chemokine |
| bFGF | 6.4[-5.8 : 20.2] | 3,858205554 | 12.7[3.0 : 23.3] | 0,107916979 | -2.6[-10.9 : 6.4] | 6,700163889 | -12.5[-27.8 : 5.9] | 2,048001397 | 6.5[-3.3 : 17.3] | 2,395766728 | Growth Factor |
| Flt-1 | -15.8[-19.5 : -11.9] | 9,03E-13 | -15.7[-18.5 : -12.9] | 9,04E-23 | -1.4[-4.6 : 1.9] | 4,772615901 | 3.7[-3.4 : 11.3] | 3,772221905 | -0.6[-4.1 : 3.0] | 8,849207537 | Growth Factor |
| GM-CSF | -7.4[-19.3 : 6.3] | 3,291423968 | -4.4[-13.6 : 5.8] | 4,625090387 | -3.7[-12.9 : 6.5] | 5,549617387 | 3.6[-16.6 : 28.5] | 9,016163294 | 9.0[-2.2 : 21.5] | 1,439419197 | Growth Factor |
| IL-7 | 8.4[0.8 : 16.6] | 0,364861796 | 11.6[5.8 : 17.8] | 7,18E-04 | -5.9[-10.8 : -0.7] | 0,315071019 | -6.2[-16.4 : 5.2] | 3,253513626 | -0.1[-5.7 : 5.8] | 11,55188165 | Growth Factor |
| PlGF | -0.3[-3.8 : 3.4] | 10,63579855 | 2.3[-0.4 : 5.1] | 1,112718917 | -1.1[-3.7 : 1.6] | 5,058229339 | -2.6[-8.0 : 3.1] | 4,305081037 | 8.9[5.8 : 12.1] | 6,60E-08 | Growth Factor |
| sICAM-1 | 4.9[-0.1 : 10.3] | 0,679515617 | 1.3[-2.3 : 5.1] | 5,687098492 | -1.6[-5.1 : 1.9] | 4,391296286 | 0.3[-7.0 : 8.2] | 11,1775741 | 14.1[9.8 : 18.5] | 2,71E-10 | Growth Factor |
| sVCAM-1 | -6.6[-10.2 : -2.9] | 0,007400538 | -12.2[-14.7 : -9.7] | 7,62E-18 | 1.2[-1.6 : 4.1] | 4,867824416 | 0.4[-5.4 : 6.6] | 10,79692043 | 6.7[3.5 : 10.0] | 3,59E-04 | Growth Factor |
| Tie-2 | -3.0[-6.6 : 0.9] | 1,520619594 | -6.9[-9.5 : -4.2] | 1,15E-05 | -1.6[-4.4 : 1.2] | 2,998233058 | 5.1[-1.1 : 11.6] | 1,293430061 | 4.0[0.8 : 7.2] | 0,147599301 | Growth Factor |
| TSLP | 9.0[-5.9 : 26.4] | 3,003056346 | -4.3[-14.1 : 6.7] | 5,178098818 | -15.4[-24.0 : -5.7] | 0,029243329 | 1.6[-19.4 : 28.0] | 10,74661628 | 14.6[1.9 : 28.8] | 0,268459212 | Growth Factor |
| VEGF | 34.0[17.5 : 52.8] | 1,61E-04 | 38.5[25.7 : 52.6] | 5,89E-10 | -1.0[-10.0 : 9.0] | 10,11372858 | -4.9[-22.6 : 17.0] | 7,643450268 | 8.5[-2.2 : 20.3] | 1,503751876 | Growth Factor |
| VEGF-C | 11.3[3.3 : 19.9] | 0,057198899 | 12.6[6.6 : 19.0] | 2,66E-04 | -1.3[-6.6 : 4.2] | 7,49598504 | 4.3[-7.2 : 17.3] | 5,759738126 | 6.0[-0.1 : 12.4] | 0,626429149 | Growth Factor |
| VEGF-D | 41.6[29.9 : 54.3] | 3,24E-14 | 24.5[16.9 : 32.6] | 1,58E-10 | -5.0[-10.7 : 1.2] | 1,327319941 | -5.5[-17.4 : 8.2] | 4,939952455 | 2.1[-4.6 : 9.2] | 6,64381811 | Growth Factor |

**Supplementary Table 9:** Model excluding those who have been on hormonal contraceptives for less than 28 days. Estimates are shown as percentage change and p values are corrected for multiple testing. Model is adjusted for age, current smoking, BMI, sample age, sample analysis date, and Danish administrative region of sampling.

| **Supplementary Table 10** | |  |  |  |  |  |  |  |  |  |  |  |  |
| --- | --- | --- | --- | --- | --- | --- | --- | --- | --- | --- | --- | --- | --- |
|  | COC low |  | COC high |  | IUD |  | POP |  | HT |  | Menopausal |  |  |
| Assay | % estimate | corrected P | % estimate | corrected P | % estimate | corrected P | % estimate | corrected P | % estimate | corrected P | % estimate | corrected P | Group |
| CRP | 314.4[241.8 : 402.4] | 2,90E-44 | 366.7[297.0 : 448.6] | 3,66E-71 | 0,300840886 | 20.8[2.4 : 42.5] | 5,926000549 | 9.5[-15.6 : 42.0] | 61.9[18.6 : 121.0] | 0,02905343 | 39.2[13.1 : 71.3] | 0,02174654 | Proinflammatory |
| SAA | 49.3[27.5 : 74.7] | 8,21E-06 | 33.0[16.5 : 51.8] | 2,94E-04 | 0,417331216 | -13.6[-24.5 : -1.0] | 2,293981328 | -13.2[-29.9 : 7.4] | 38.7[7.5 : 78.9] | 0,143238828 | 19.1[0.5 : 41.1] | 0,530993609 | Proinflammatory |
| IL-1A | -3.7[-16.0 : 10.5] | 7,123364842 | -5.8[-16.1 : 5.8] | 3,772592007 | 10,26241259 | -1.1[-12.2 : 11.4] | 9,872900699 | -2.1[-19.1 : 18.3] | -1.2[-21.2 : 23.9] | 10,98699143 | 3.8[-10.7 : 20.6] | 7,517483332 | Proinflammatory |
| IL-1B | 0.7[-21.2 : 28.9] | 11,44372615 | -2.5[-20.8 : 20.1] | 9,759058209 | 10,62445019 | -1.6[-20.5 : 22.0] | 5,122368078 | -12.9[-38.1 : 22.5] | -6.9[-38.0 : 39.8] | 8,77976981 | 12.7[-14.0 : 47.5] | 4,627924929 | Proinflammatory |
| IL-1RA | -1.7[-10.3 : 7.7] | 8,492948205 | -6.7[-13.6 : 0.8] | 0,961806279 | 4,890361947 | 3.4[-4.5 : 12.0] | 2,269939443 | -8.1[-19.0 : 4.3] | -3.8[-17.4 : 12.0] | 7,399508205 | 2.6[-7.2 : 13.4] | 7,3773434 | Proinflammatory |
| IL-3 | -6.6[-23.1 : 13.3] | 5,845754436 | -6.3[-20.5 : 10.4] | 5,238262436 | 8,217215406 | 3.6[-12.6 : 22.7] | 6,762255409 | -7.6[-29.5 : 21.0] | -31.8[-50.6 : -5.7] | 0,247108597 | 7.0[-13.6 : 32.5] | 6,403725427 | Proinflammatory |
| IL-6 | -8.2[-20.9 : 6.7] | 3,168935677 | -8.9[-19.7 : 3.4] | 1,792671434 | 1,632134334 | -9.4[-20.5 : 3.2] | 7,866310954 | -4.6[-22.5 : 17.4] | 2.2[-20.2 : 30.9] | 10,33947879 | 13.2[-3.9 : 33.4] | 1,64622508 | Proinflammatory |
| IL-12 | 4.1[-4.5 : 13.4] | 4,369265725 | 0.9[-6.2 : 8.5] | 9,671651011 | 8,853608267 | 1.3[-6.0 : 9.2] | 0,902338398 | 11.4[-1.1 : 25.5] | 17.3[1.8 : 35.2] | 0,332533574 | 14.5[4.2 : 25.8] | 0,058241613 | Proinflammatory |
| IL-12p70 | -16.0[-32.5 : 4.5] | 1,407222767 | -17.3[-31.2 : -0.5] | 0,529577223 | 8,142706138 | -3.9[-20.6 : 16.2] | 7,519968357 | -7.2[-31.5 : 25.5] | 1.5[-29.3 : 45.5] | 11,24820761 | -4.6[-24.9 : 21.2] | 8,392074817 | Proinflammatory |
| IL-15 | -1.7[-6.1 : 2.9] | 5,506937639 | -0.5[-4.3 : 3.4] | 9,446257332 | 0,022785207 | -6.1[-9.7 : -2.3] | 1,256533979 | -5.1[-10.9 : 1.1] | -2.3[-9.3 : 5.3] | 6,53653214 | -0.8[-5.6 : 4.3] | 9,110450734 | Proinflammatory |
| TNF-A | -2.7[-7.7 : 2.6] | 3,774192028 | -5.9[-10.0 : -1.5] | 0,103272556 | 0,830869033 | -4.2[-8.5 : 0.3] | 6,149153434 | -2.4[-9.4 : 5.0] | 14.2[4.6 : 24.8] | 0,03762049 | 10.8[4.6 : 17.5] | 0,00659613 | Proinflammatory |
| TNF-B | -9.6[-20.5 : 2.7] | 1,459809611 | -11.5[-20.6 : -1.4] | 0,318069159 | 3,994110667 | -5.4[-15.3 : 5.8] | 10,90563641 | 1.0[-15.4 : 20.6] | 6.9[-13.5 : 32.0] | 6,448127908 | 9.8[-4.5 : 26.3] | 2,261626975 | Proinflammatory |
| IFN-G | 8.8[-4.7 : 24.2] | 2,751716261 | 2.5[-8.3 : 14.7] | 8,566948394 | 3,656689191 | -6.1[-16.3 : 5.3] | 10,92832303 | -1.9[-18.3 : 17.9] | 4.5[-16.0 : 30.1] | 8,966224715 | 10.7[-4.2 : 28.0] | 2,19425369 | T derived |
| IL-2 | 5.1[-15.6 : 30.8] | 8,520844065 | 0.8[-16.2 : 21.3] | 12,08177781 | 2,648775119 | 13.1[-6.5 : 36.9] | 7,428835124 | -8.4[-32.3 : 24.1] | 61.4[12.5 : 131.7] | 0,122340542 | 7.0[-15.8 : 35.9] | 7,5576703 | T derived |
| IL-4 | 6.2[-13.8 : 30.9] | 7,418247671 | 10.3[-7.6 : 31.6] | 3,591444611 | 6,490602939 | 6.5[-11.2 : 27.7] | 3,905649308 | 16.5[-12.7 : 55.5] | 23.6[-12.4 : 74.4] | 2,97046144 | 12.5[-10.5 : 41.4] | 4,065351505 | T derived |
| IL-5 | 4.2[-7.5 : 17.3] | 6,489538418 | 1.8[-7.9 : 12.6] | 9,370801418 | 10,50334644 | -1.3[-11.0 : 9.5] | 4,019996775 | -8.2[-22.1 : 8.3] | 0.4[-17.5 : 22.2] | 12,59065153 | -6.2[-17.6 : 6.9] | 4,375960591 | T derived |
| IL-9 | -11.8[-29.1 : 9.7] | 3,372409925 | -9.2[-24.5 : 9.2] | 3,977141015 | 9,885568135 | 3.0[-14.8 : 24.6] | 6,812179503 | -9.3[-33.0 : 22.6] | 6.9[-25.7 : 53.7] | 9,363651442 | 22.3[-3.8 : 55.5] | 1,297802099 | T derived |
| IL-10 | -14.3[-25.8 : -1.0] | 0,461780833 | -9.9[-20.2 : 1.7] | 1,20438484 | 4,338024293 | -6.0[-17.1 : 6.6] | 4,449898823 | 10.1[-9.8 : 34.4] | 11.9[-11.8 : 41.9] | 4,603663495 | 14.3[-2.4 : 33.8] | 1,257254239 | T derived |
| IL-13 | -4.9[-21.1 : 14.6] | 7,774605003 | 9.2[-6.7 : 27.8] | 3,577652086 | 2,290237341 | 11.8[-4.9 : 31.5] | 3,434680085 | 15.8[-10.5 : 49.9] | -4.2[-29.6 : 30.3] | 10,1967913 | -8.2[-25.1 : 12.6] | 5,372880292 | T derived |
| IL-16 | -2.6[-12.8 : 8.8] | 8,368837108 | -10.0[-18.0 : -1.1] | 0,365212303 | 1,584695338 | 7.9[-2.0 : 18.8] | 8,75350229 | -3.2[-17.0 : 12.8] | 11.7[-6.9 : 34.2] | 3,047996907 | 17.3[3.9 : 32.4] | 0,129551486 | T derived |
| IL-17A | 9.1[-2.6 : 22.1] | 1,702341366 | 6.2[-3.4 : 16.9] | 2,766348181 | 5,384427896 | 4.2[-5.6 : 14.9] | 2,820755745 | 10.3[-5.6 : 29.0] | 28.6[6.7 : 54.9] | 0,106133191 | 13.3[0.2 : 28.2] | 0,611525162 | T derived |
| IL-17A/F | 7.8[-9.8 : 28.9] | 5,325632491 | 7.6[-7.4 : 25.2] | 4,408540369 | 11,83210756 | 0.9[-13.6 : 17.9] | 3,155801275 | -13.7[-32.5 : 10.5] | -0.2[-25.8 : 34.4] | 12,88304042 | 10.3[-9.3 : 34.1] | 4,245895676 | T derived |
| IL-17B | -18.9[-30.8 : -5.0] | 0,122627518 | -15.7[-26.3 : -3.5] | 0,167492729 | 1,52281892 | -10.5[-22.0 : 2.8] | 0,870551262 | -18.6[-34.7 : 1.4] | 14.9[-11.7 : 49.6] | 3,909912768 | 11.7[-6.2 : 33.0] | 2,772171172 | T derived |
| IL-17C | 9.3[-8.7 : 30.8] | 4,305245126 | -4.0[-17.5 : 11.8] | 7,813996637 | 8,136285178 | -3.8[-17.8 : 12.5] | 6,005493449 | -8.9[-28.9 : 16.8] | 7.0[-20.7 : 44.3] | 8,568026015 | 7.6[-11.7 : 31.0] | 6,09899317 | T derived |
| IL-17D | -6.1[-19.4 : 9.4] | 5,47610556 | -10.7[-21.6 : 1.6] | 1,107156672 | 0,555764606 | -12.9[-23.8 : -0.4] | 4,995826312 | -9.0[-26.3 : 12.5] | -8.4[-29.0 : 18.3] | 6,52554246 | 9.5[-7.4 : 29.6] | 3,750880081 | T derived |
| CCL2 | 6.9[-0.6 : 15.1] | 0,731523465 | 1.5[-4.6 : 7.9] | 6,426470672 | 5,709131951 | -1.8[-7.7 : 4.5] | 8,527424827 | -0.9[-10.4 : 9.5] | 13.1[0.6 : 27.1] | 0,396852397 | 15.4[6.8 : 24.8] | 0,003265391 | Chemokine |
| CCL3 | 1.5[-6.4 : 10.1] | 7,099558705 | 4.4[-2.4 : 11.7] | 2,078792458 | 4,973436712 | -2.4[-8.9 : 4.6] | 1,694941318 | 8.0[-3.3 : 20.7] | 5.8[-7.1 : 20.4] | 3,962796643 | 5.8[-2.9 : 15.4] | 1,998097747 | Chemokine |
| CCL4 | -6.2[-14.2 : 2.6] | 1,612240416 | -10.4[-16.8 : -3.5] | 0,037815722 | 1,257228077 | -5.7[-12.6 : 1.7] | 1,639433359 | -8.3[-18.8 : 3.6] | 6.1[-8.0 : 22.3] | 4,154406095 | 5.5[-4.1 : 16.0] | 2,685644252 | Chemokine |
| CCL11 | -7.6[-14.1 : -0.6] | 0,329708869 | -11.8[-17.0 : -6.3] | 4,98E-04 | 1,270451318 | -4.7[-10.4 : 1.4] | 6,632265144 | 2.2[-7.4 : 12.9] | -2.7[-13.3 : 9.3] | 6,481712709 | 5.7[-2.2 : 14.2] | 1,627940885 | Chemokine |
| CCL13 | -11.6[-19.4 : -3.1] | 0,082351034 | -12.5[-18.9 : -5.6] | 0,006245733 | 5,9664514 | 2.1[-5.5 : 10.4] | 1,794936031 | -8.2[-19.0 : 4.0] | 8.3[-6.4 : 25.4] | 2,830530682 | 10.0[-0.2 : 21.3] | 0,549256001 | Chemokine |
| CCL17 | -23.2[-32.4 : -12.8] | 5,10E-04 | -21.1[-29.1 : -12.3] | 1,27E-04 | 9,163833075 | -0.6[-10.8 : 10.8] | 4,456915035 | -6.5[-21.5 : 11.2] | 13.4[-7.5 : 39.1] | 2,256491586 | 21.1[5.7 : 38.7] | 0,059450993 | Chemokine |
| CCL22 | 3.1[-2.8 : 9.5] | 3,069372786 | -2.9[-7.6 : 2.0] | 2,454664897 | 2,379086607 | -3.0[-7.8 : 2.0] | 3,147405842 | -4.1[-11.5 : 4.0] | 20.8[9.8 : 32.8] | 9,87E-04 | 17.3[10.1 : 24.9] | 9,04E-06 | Chemokine |
| CCL26 | -2.7[-14.0 : 10.1] | 6,657626305 | -7.4[-16.5 : 2.7] | 1,447445085 | 5,952207281 | -2.8[-12.5 : 8.0] | 5,795771082 | -4.6[-19.4 : 12.8] | -2.3[-19.8 : 19.0] | 8,139902721 | -6.3[-17.9 : 6.9] | 3,317702409 | Chemokine |
| IL-8 | -2.0[-13.6 : 11.0] | 7,456328312 | 2.9[-7.5 : 14.4] | 5,993905922 | 9,974393874 | 0.0[-10.3 : 11.5] | 1,893619578 | -11.0[-25.1 : 5.9] | 4.6[-14.9 : 28.6] | 6,677090391 | 13.3[-1.2 : 30.0] | 0,734883319 | Chemokine |
| CXCL10 | -4.5[-12.8 : 4.4] | 3,102055535 | -9.0[-15.6 : -1.9] | 0,138558417 | 7,227201555 | -1.4[-8.6 : 6.5] | 8,391989158 | -1.3[-12.6 : 11.6] | 7.5[-6.9 : 24.1] | 3,255328474 | 11.5[1.3 : 22.7] | 0,263011256 | Chemokine |
| bFGF | 5.5[-9.2 : 22.6] | 5,839101399 | 12.1[-1.3 : 27.4] | 0,936645079 | 8,303556098 | -2.6[-14.5 : 11.0] | 2,725449356 | -12.0[-28.6 : 8.3] | 15.0[-10.3 : 47.4] | 3,228935843 | 12.0[-4.9 : 32.1] | 2,104045979 | Growth Factor |
| Flt-1 | -17.7[-22.3 : -12.7] | 9,69E-10 | -17.9[-21.8 : -13.7] | 1,13E-13 | 2,016212874 | -3.5[-8.3 : 1.5] | 11,229538 | 0.3[-7.5 : 8.8] | -7.7[-16.2 : 1.6] | 1,234638198 | 0.2[-6.0 : 6.8] | 11,31942758 | Growth Factor |
| GM-CSF | -15.1[-28.3 : 0.5] | 0,685109077 | -16.1[-27.3 : -3.3] | 0,189537661 | 0,821755098 | -12.8[-24.7 : 1.0] | 4,84103374 | -9.5[-28.4 : 14.4] | 4.6[-20.8 : 38.3] | 8,997153868 | 1.0[-16.0 : 21.5] | 10,99022013 | Growth Factor |
| IL-7 | 7.2[-2.2 : 17.4] | 1,674526521 | 10.7[2.5 : 19.7] | 0,121242957 | 1,269343475 | -6.4[-13.6 : 1.4] | 4,280985501 | -5.8[-17.0 : 7.0] | -3.3[-16.9 : 12.5] | 7,922884526 | 3.5[-6.3 : 14.5] | 5,953600818 | Growth Factor |
| PlGF | -4.8[-9.0 : -0.4] | 0,377304938 | -1.8[-5.5 : 2.0] | 4,068960075 | 0,93429892 | -3.5[-7.2 : 0.4] | 1,016102657 | -5.3[-11.0 : 0.8] | 8.3[0.5 : 16.6] | 0,427570198 | 9.0[3.8 : 14.5] | 0,006984141 | Growth Factor |
| sICAM-1 | 4.1[-2.3 : 11.0] | 2,560902555 | 0.3[-4.9 : 5.8] | 10,85864735 | 9,618363807 | -0.7[-6.0 : 4.9] | 11,18532203 | -0.4[-8.6 : 8.6] | 21.1[9.2 : 34.2] | 0,003270891 | 21.3[13.3 : 30.0] | 4,38E-07 | Growth Factor |
| sVCAM-1 | -7.7[-11.9 : -3.3] | 0,008811271 | -12.5[-15.9 : -9.0] | 3,08E-10 | 7,098370464 | 1.1[-2.9 : 5.2] | 11,83496324 | -0.1[-6.1 : 6.4] | 11.5[3.4 : 20.2] | 0,056361067 | 8.3[3.0 : 13.8] | 0,023238287 | Growth Factor |
| Tie-2 | -5.0[-9.7 : -0.2] | 0,499558583 | -8.4[-12.2 : -4.5] | 5,30E-04 | 3,34170531 | -2.4[-6.5 : 2.0] | 4,455160278 | 3.2[-3.7 : 10.6] | 5.0[-3.3 : 14.0] | 2,984211114 | 5.5[-0.1 : 11.4] | 0,649940531 | Growth Factor |
| TSLP | 6.4[-11.8 : 28.4] | 6,212892523 | -6.2[-20.0 : 10.0] | 5,198452188 | 0,749783735 | -14.4[-27.4 : 0.8] | 11,22514418 | 1.1[-22.1 : 31.1] | -2.0[-28.4 : 34.1] | 10,79780562 | 20.0[-2.4 : 47.6] | 0,997888261 | Growth Factor |
| VEGF | 25.5[6.7 : 47.5] | 0,071775977 | 31.4[14.5 : 50.7] | 0,001181479 | 11,25358051 | 0.6[-12.6 : 15.8] | 8,564878176 | -4.1[-23.4 : 20.0] | 12.5[-13.9 : 47.0] | 4,634669915 | 19.6[0.2 : 42.8] | 0,567320231 | Growth Factor |
| VEGF-C | 7.6[-1.8 : 18.0] | 1,409791816 | 9.8[1.6 : 18.7] | 0,219015546 | 5,218387949 | -3.1[-10.6 : 4.9] | 9,528719739 | 1.7[-10.5 : 15.5] | 1.7[-12.7 : 18.3] | 9,968590441 | 8.0[-2.4 : 19.4] | 1,622766579 | Growth Factor |
| VEGF-D | 45.7[31.0 : 62.1] | 6,18E-11 | 31.8[20.5 : 44.3] | 2,62E-08 | 6,592566501 | -2.8[-11.4 : 6.6] | 10,42481368 | -1.2[-14.8 : 14.4] | 7.6[-9.7 : 28.3] | 4,947403 | -3.5[-14.1 : 8.4] | 6,599339438 | Growth Factor |

**Supplementary Table 10:** Model that only allows those who have never used hormonal contraceptives in the non-user group and in the menopausal group. Estimates are shown as percentage change and p values are corrected for multiple testing. Model is adjusted for age, current smoking, BMI, sample age, sample analysis date, and Danish administrative region of sampling.

| **Supplementary Table 11** | |  |  |  |  |  |  |  |  |  |  |  |  |
| --- | --- | --- | --- | --- | --- | --- | --- | --- | --- | --- | --- | --- | --- |
|  | COC low |  | COC high |  | IUD |  | POP |  | HT |  | Menopausal |  |  |
| Assay | % estimate | corrected P | % estimate | corrected P | % estimate | corrected P | % estimate | corrected P | % estimate | corrected P | % estimate | corrected P | Group |
| CRP | 319.9[248.4 : 406.0] | 5,56E-48 | 368.1[301.2 : 446.3] | 3,24E-78 | 18.7[1.6 : 38.8] | 0,372321738 | 8.5[-16.0 : 40.1] | 6,404095479 | 54.0[13.8 : 108.3] | 0,062027738 | 35.0[11.7 : 63.0] | 0,022780603 | Proinflammatory |
| SAA | 52.7[31.2 : 77.6] | 5,55E-07 | 35.2[19.3 : 53.2] | 2,97E-05 | -13.2[-23.5 : -1.5] | 0,336471115 | -12.5[-28.9 : 7.7] | 2,502489167 | 36.5[6.8 : 74.4] | 0,155535185 | 19.0[2.1 : 38.7] | 0,31490193 | Proinflammatory |
| IL-1A | -5.4[-17.4 : 8.4] | 5,087680051 | -6.5[-16.5 : 4.7] | 2,963003829 | -0.7[-11.5 : 11.4] | 10,85530998 | -2.0[-19.1 : 18.8] | 10,06828694 | 0.3[-20.0 : 25.6] | 11,7777994 | 1.8[-11.4 : 17.0] | 9,619136489 | Proinflammatory |
| IL-1B | 6.0[-16.5 : 34.5] | 7,612242863 | 0.3[-17.8 : 22.4] | 11,71618254 | 0.3[-18.0 : 22.8] | 11,68288931 | -11.0[-36.5 : 24.6] | 5,957203144 | -4.6[-35.8 : 41.7] | 9,786836349 | 13.2[-11.4 : 44.6] | 3,840970871 | Proinflammatory |
| IL-1RA | -1.4[-9.6 : 7.6] | 9,040388775 | -6.2[-12.8 : 0.8] | 0,996415718 | 4.0[-3.4 : 12.0] | 3,523008413 | -7.5[-18.2 : 4.6] | 2,561686485 | -3.1[-16.3 : 12.2] | 8,080728918 | 2.9[-5.9 : 12.6] | 6,370746143 | Proinflammatory |
| IL-3 | -7.6[-23.2 : 11.3] | 4,881773726 | -7.2[-20.6 : 8.4] | 4,12528301 | 2.6[-12.3 : 20.2] | 8,94553436 | -8.1[-29.4 : 19.5] | 6,328907993 | -31.8[-50.1 : -6.9] | 0,192848176 | 4.2[-14.0 : 26.2] | 8,103489622 | Proinflammatory |
| IL-6 | -5.9[-18.3 : 8.5] | 4,856890703 | -6.8[-17.2 : 4.9] | 2,917387588 | -7.5[-18.0 : 4.4] | 2,471868702 | -2.4[-20.2 : 19.2] | 9,7106065 | 5.0[-17.1 : 32.9] | 8,251723523 | 17.3[1.4 : 35.7] | 0,380681409 | Proinflammatory |
| IL-12 | 5.0[-3.3 : 14.1] | 2,941164415 | 1.6[-5.2 : 8.8] | 7,860010499 | 1.9[-5.0 : 9.3] | 7,168865684 | 12.4[0.0 : 26.3] | 0,605364956 | 17.7[2.6 : 35.1] | 0,239810328 | 14.5[5.2 : 24.7] | 0,021456777 | Proinflammatory |
| IL-12p70 | -15.6[-31.6 : 4.2] | 1,374051074 | -17.7[-31.0 : -2.0] | 0,350187985 | -4.8[-20.4 : 13.7] | 7,0293911 | -7.9[-31.6 : 23.9] | 7,02436233 | -0.6[-29.9 : 40.9] | 11,65215869 | -6.1[-24.3 : 16.5] | 6,80787382 | Proinflammatory |
| IL-15 | -2.1[-6.3 : 2.4] | 4,322728337 | -0.8[-4.4 : 2.9] | 8,015050012 | -6.2[-9.6 : -2.6] | 0,011255514 | -5.2[-11.0 : 0.9] | 1,116485998 | -2.1[-9.1 : 5.4] | 6,877268293 | -1.7[-6.0 : 2.9] | 5,636442213 | Proinflammatory |
| TNF-A | -2.1[-6.9 : 3.0] | 5,06016564 | -5.4[-9.4 : -1.3] | 0,118316831 | -3.7[-7.8 : 0.5] | 0,998265406 | -1.9[-8.7 : 5.4] | 7,118879849 | 14.7[5.4 : 24.8] | 0,018646587 | 11.6[5.9 : 17.6] | 4,95E-04 | Proinflammatory |
| TNF-B | -8.0[-18.7 : 4.1] | 2,247134358 | -10.2[-19.1 : -0.5] | 0,483351956 | -4.0[-13.6 : 6.6] | 5,321018232 | 2.9[-13.6 : 22.6] | 8,957885756 | 8.6[-11.6 : 33.4] | 5,16644879 | 11.8[-1.6 : 26.9] | 1,036029292 | Proinflammatory |
| IFN-G | 8.4[-4.5 : 23.0] | 2,75043245 | 2.2[-8.1 : 13.5] | 8,9758198 | -6.3[-15.8 : 4.3] | 3,008150778 | -2.3[-18.2 : 16.8] | 10,42942277 | 4.3[-15.4 : 28.7] | 8,984752322 | 9.0[-4.3 : 24.1] | 2,498786013 | T derived |
| IL-2 | 1.3[-17.8 : 24.8] | 11,73865953 | -2.7[-18.2 : 15.8] | 9,849883708 | 8.7[-8.9 : 29.8] | 4,591345113 | -11.5[-34.1 : 18.9] | 5,429699091 | 52.8[8.1 : 116.1] | 0,214355527 | 2.2[-17.5 : 26.6] | 10,96175029 | T derived |
| IL-4 | 1.3[-17.0 : 23.8] | 11,66400501 | 5.3[-10.9 : 24.5] | 7,038183216 | 0.0[-15.6 : 18.4] | 12,99229717 | 10.7[-16.5 : 46.8] | 6,236096003 | 12.5[-19.3 : 56.7] | 6,332292817 | 3.9[-15.4 : 27.5] | 9,309983721 | T derived |
| IL-5 | 5.8[-5.5 : 18.5] | 4,238161872 | 3.2[-6.1 : 13.4] | 6,741240546 | -0.3[-9.5 : 9.7] | 12,261324 | -7.0[-20.7 : 9.1] | 4,846236779 | 0.9[-16.4 : 21.7] | 12,08163111 | -7.8[-17.9 : 3.5] | 2,202257431 | T derived |
| IL-9 | -13.5[-29.7 : 6.5] | 2,239846047 | -11.0[-25.1 : 5.9] | 2,456326605 | 2.2[-14.3 : 21.9] | 10,49943501 | -10.5[-33.2 : 19.9] | 5,955587992 | 7.5[-24.1 : 52.3] | 8,869192514 | 23.6[-0.2 : 53.1] | 0,680083682 | T derived |
| IL-10 | -16.0[-27.0 : -3.4] | 0,188495397 | -11.4[-21.2 : -0.5] | 0,537420025 | -7.7[-18.0 : 3.9] | 2,403065767 | 8.3[-11.1 : 31.9] | 5,589900677 | 8.4[-14.1 : 36.7] | 6,463542931 | 8.5[-6.0 : 25.3] | 3,416440058 | T derived |
| IL-13 | -8.2[-23.1 : 9.6] | 4,458367205 | 5.7[-8.8 : 22.6] | 5,981097609 | 8.6[-6.6 : 26.1] | 3,686053912 | 12.6[-12.3 : 44.6] | 4,574998686 | -6.0[-29.9 : 26.2] | 8,85497738 | -7.6[-22.9 : 10.9] | 5,155702987 | T derived |
| IL-16 | -3.6[-13.2 : 7.2] | 6,534043849 | -11.1[-18.6 : -2.9] | 0,115930824 | 6.1[-3.0 : 16.1] | 2,51781791 | -4.6[-17.8 : 10.7] | 6,956202216 | 8.6[-8.9 : 29.4] | 4,624169017 | 12.7[1.1 : 25.6] | 0,403648112 | T derived |
| IL-17A | 11.2[-0.1 : 23.8] | 0,670685395 | 8.2[-1.0 : 18.3] | 1,069908419 | 6.0[-3.2 : 16.1] | 2,692047938 | 12.5[-3.3 : 30.8] | 1,661993886 | 30.3[9.1 : 55.6] | 0,045965678 | 14.8[2.9 : 28.2] | 0,177374891 | T derived |
| IL-17A/F | 1.4[-14.8 : 20.6] | 11,38526748 | 2.1[-11.7 : 17.9] | 10,16999013 | -5.3[-18.2 : 9.7] | 6,101521376 | -18.8[-36.4 : 3.6] | 1,22607624 | -9.4[-32.3 : 21.1] | 6,549415922 | -0.3[-16.6 : 19.2] | 12,66910403 | T derived |
| IL-17B | -20.6[-31.7 : -7.7] | 0,033790588 | -17.4[-27.1 : -6.3] | 0,036869554 | -12.7[-23.1 : -0.8] | 0,481910789 | -20.5[-35.7 : -1.7] | 0,447367976 | 11.3[-13.4 : 43.1] | 5,252128852 | 8.9[-6.7 : 27.1] | 3,644085034 | T derived |
| IL-17C | 5.2[-11.4 : 24.9] | 7,29299781 | -7.3[-19.6 : 7.0] | 3,894307246 | -7.0[-19.6 : 7.5] | 4,211962333 | -11.7[-30.7 : 12.4] | 4,048175385 | 3.0[-22.7 : 37.3] | 10,92814149 | 1.4[-15.0 : 20.9] | 11,41827497 | T derived |
| IL-17D | -5.0[-17.9 : 10.0] | 6,440750016 | -9.8[-20.2 : 2.0] | 1,300996256 | -12.0[-22.3 : -0.4] | 0,569239738 | -8.1[-25.3 : 13.1] | 5,522978065 | -7.8[-27.9 : 17.9] | 6,718890238 | 8.7[-6.6 : 26.4] | 3,653973102 | T derived |
| CCL2 | 7.0[-1.3 : 16.1] | 0,997175758 | 1.2[-5.4 : 8.1] | 7,348422608 | -1.9[-8.2 : 4.9] | 5,823905971 | -1.4[-11.9 : 10.3] | 8,039403273 | 13.8[0.0 : 29.6] | 0,502093011 | 14.5[5.6 : 24.2] | 0,011121457 | Chemokine |
| CCL3 | 0.4[-7.2 : 8.7] | 9,132426617 | 3.7[-2.8 : 10.7] | 2,685295623 | -3.0[-9.1 : 3.5] | 3,603146734 | 7.4[-3.7 : 19.8] | 2,01282472 | 5.0[-7.5 : 19.1] | 4,505359677 | 4.7[-3.3 : 13.3] | 2,563825612 | Chemokine |
| CCL4 | -5.8[-13.7 : 2.8] | 1,773628341 | -10.2[-16.4 : -3.5] | 0,033861229 | -5.4[-11.9 : 1.7] | 1,342891852 | -8.2[-18.6 : 3.6] | 1,658244244 | 6.5[-7.3 : 22.5] | 3,735936104 | 5.2[-3.7 : 14.8] | 2,601718312 | Chemokine |
| CCL11 | -8.8[-15.0 : -2.1] | 0,110336634 | -13.0[-17.9 : -7.8] | 2,67E-05 | -6.3[-11.6 : -0.7] | 0,286413158 | 0.5[-8.8 : 10.9] | 9,154937896 | -5.0[-15.1 : 6.4] | 3,759667125 | 2.3[-4.7 : 9.9] | 5,229452014 | Chemokine |
| CCL13 | -12.5[-19.9 : -4.4] | 0,03128098 | -13.9[-19.9 : -7.4] | 5,81E-04 | -0.5[-7.5 : 7.1] | 8,965393721 | -10.4[-20.8 : 1.3] | 0,790632615 | 4.1[-9.7 : 19.9] | 5,833910934 | 4.5[-4.4 : 14.2] | 3,325603407 | Chemokine |
| CCL17 | -24.2[-33.0 : -14.2] | 1,16E-04 | -22.7[-30.2 : -14.4] | 7,38E-06 | -2.8[-12.3 : 7.7] | 5,833943973 | -9.1[-23.4 : 7.9] | 2,749455017 | 9.7[-10.0 : 33.7] | 3,604105817 | 15.4[1.9 : 30.6] | 0,242022815 | Chemokine |
| CCL22 | 3.3[-2.6 : 9.6] | 2,796137373 | -3.1[-7.7 : 1.7] | 2,050330374 | -3.5[-8.1 : 1.4] | 1,554569179 | -4.7[-12.2 : 3.5] | 2,507600459 | 19.6[8.7 : 31.5] | 0,002339235 | 15.0[8.3 : 22.0] | 4,69E-05 | Chemokine |
| CCL26 | -3.7[-14.7 : 8.7] | 5,443134041 | -8.2[-16.9 : 1.4] | 0,927645877 | -4.2[-13.3 : 5.9] | 4,036693913 | -5.7[-20.3 : 11.6] | 4,943918744 | -4.1[-21.0 : 16.5] | 6,740119354 | -8.6[-19.1 : 3.3] | 1,48507802 | Chemokine |
| IL-8 | -2.1[-13.2 : 10.3] | 7,228554093 | 2.5[-7.2 : 13.3] | 6,211438001 | -0.7[-10.3 : 9.9] | 8,878079743 | -11.5[-25.2 : 4.8] | 1,579383276 | 2.7[-15.8 : 25.3] | 7,900322003 | 8.5[-4.0 : 22.7] | 1,915627258 | Chemokine |
| CXCL10 | -4.2[-12.5 : 4.9] | 3,526161822 | -8.6[-15.1 : -1.5] | 0,180770348 | -1.0[-8.1 : 6.7] | 7,975700136 | -1.1[-12.7 : 12.1] | 8,625408118 | 7.8[-6.7 : 24.6] | 3,099723001 | 10.8[1.2 : 21.3] | 0,273020338 | Chemokine |
| bFGF | 7.3[-6.9 : 23.7] | 3,975507703 | 14.1[1.3 : 28.5] | 0,356749691 | -1.8[-13.0 : 10.8] | 9,187107127 | -11.1[-27.3 : 8.7] | 3,018780228 | 13.9[-10.1 : 44.2] | 3,379686926 | 10.6[-4.4 : 28.0] | 2,09404638 | Growth Factor |
| Flt-1 | -18.2[-22.5 : -13.6] | 9,83E-12 | -18.3[-22.0 : -14.5] | 7,66E-17 | -4.2[-8.5 : 0.4] | 0,860272 | -0.4[-7.8 : 7.6] | 10,97156913 | -8.6[-16.5 : 0.1] | 0,627277547 | -0.7[-6.2 : 5.0] | 9,532009846 | Growth Factor |
| GM-CSF | -13.1[-26.1 : 2.1] | 1,063796997 | -14.4[-25.2 : -2.1] | 0,280147586 | -10.7[-22.1 : 2.4] | 1,250850944 | -6.8[-25.8 : 17.1] | 6,570865451 | 8.4[-17.1 : 41.8] | 6,651365125 | 5.8[-10.4 : 24.9] | 6,064035517 | Growth Factor |
| IL-7 | 6.1[-2.8 : 15.8] | 2,227486077 | 9.5[1.8 : 17.8] | 0,182470534 | -7.9[-14.5 : -0.8] | 0,350902179 | -7.1[-17.9 : 5.1] | 2,920908762 | -5.7[-18.5 : 9.0] | 5,112926008 | -0.1[-8.7 : 9.2] | 11,710426 | Growth Factor |
| PlGF | -3.6[-7.7 : 0.6] | 1,126502532 | -0.8[-4.3 : 2.8] | 7,821232741 | -3.0[-6.4 : 0.7] | 1,304766615 | -4.6[-10.3 : 1.4] | 1,544797905 | 8.3[0.8 : 16.3] | 0,359312277 | 9.0[4.3 : 13.9] | 0,001738946 | Growth Factor |
| sICAM-1 | 4.1[-2.0 : 10.5] | 2,285467111 | 0.0[-4.8 : 5.1] | 11,93554298 | -1.4[-6.2 : 3.7] | 7,141194955 | -1.0[-8.8 : 7.5] | 9,718010799 | 19.5[8.4 : 31.8] | 0,004028589 | 20.4[13.3 : 28.0] | 2,97E-08 | Growth Factor |
| sVCAM-1 | -7.7[-11.7 : -3.5] | 0,004733088 | -12.7[-15.9 : -9.4] | 6,53E-12 | 0.9[-2.7 : 4.8] | 7,44680357 | -0.3[-6.2 : 6.0] | 11,14948614 | 11.3[3.6 : 19.6] | 0,041895496 | 8.5[3.7 : 13.4] | 0,004856861 | Growth Factor |
| Tie-2 | -4.5[-8.9 : 0.2] | 0,698188897 | -8.1[-11.6 : -4.4] | 3,45E-04 | -2.2[-6.0 : 1.8] | 3,262050842 | 3.3[-3.3 : 10.4] | 4,046089936 | 5.0[-2.9 : 13.5] | 2,697750515 | 5.3[0.4 : 10.5] | 0,423756657 | Growth Factor |
| TSLP | 2.9[-14.0 : 23.1] | 9,059223338 | -10.5[-22.9 : 4.0] | 1,769488644 | -19.4[-30.8 : -6.2] | 0,064818598 | -4.0[-25.5 : 23.6] | 8,987042441 | -9.8[-33.2 : 21.8] | 5,995919561 | 8.4[-9.9 : 30.4] | 4,687884885 | Growth Factor |
| VEGF | 24.5[6.7 : 45.3] | 0,06595652 | 29.2[13.6 : 47.0] | 0,001225774 | -2.5[-14.4 : 11.2] | 8,49641985 | -6.6[-24.9 : 16.2] | 6,507931195 | 5.9[-18.1 : 36.9] | 7,954135032 | 9.3[-6.7 : 28.1] | 3,260704198 | Growth Factor |
| VEGF-C | 7.8[-1.2 : 17.6] | 1,091662105 | 9.7[2.0 : 18.0] | 0,148301933 | -3.8[-10.7 : 3.5] | 3,60292535 | 1.2[-10.5 : 14.5] | 10,15848348 | -0.5[-13.9 : 15.0] | 11,38508127 | 5.4[-3.6 : 15.2] | 2,993410524 | Growth Factor |
| VEGF-D | 48.8[34.3 : 64.7] | 4,14E-13 | 33.9[23.0 : 45.9] | 2,85E-10 | -1.0[-9.2 : 7.9] | 9,754754444 | 0.4[-13.1 : 15.9] | 11,51547325 | 10.2[-7.0 : 30.5] | 3,162356705 | -1.1[-10.9 : 9.8] | 10,05333632 | Growth Factor |

**Supplementary Table 11:** Model similar to the one in Supplementary Table 10 but allowing for one previous prescription while still being counted as a never-user. Estimates are shown as percentage change and p values are corrected for multiple testing. Model is adjusted for age, current smoking, BMI, sample age, sample analysis date, and Danish administrative region of sampling.

| **Supplementary Table 12** | | |  |  |  |  |  |  |  |  |  |  |  |  |  |  |  |
| --- | --- | --- | --- | --- | --- | --- | --- | --- | --- | --- | --- | --- | --- | --- | --- | --- | --- |
|  | G02BA03 | n=387 | G03AA07 | n=315 | G03AA09 | n=96 | G03AA10 | n=181 | G03AA11 | n=52 | G03AA12 | n=58 | G03AC09 | n=50 | Menopausal | n=1487 | group |
| Assay | % estimate | Corrected P | % estimate | Corrected P | % estimate | Corrected P | % estimate | Corrected P | % estimate | Corrected P | % estimate | Corrected P | % estimate | Corrected P | % estimate | Corrected P |  |
| CRP | 4.6[-6.5 : 17.2] | 5,178969756 | 280.7[233.2 : 335.0] | **5,02E-81** | 366.4[278.1 : 475.4] | **1,26E-44** | 321.9[260.1 : 394.4] | **4,05E-67** | 383.0[266.9 : 536.0] | **1,04E-27** | 303.5[210.0 : 425.4] | **8,89E-24** | -1.4[-25.4 : 30.3] | 11,06976652 | 16.8[3.5 : 31.9] | 0,141323588 | Proinflammatory |
| SAA | -10.8[-18.8 : -2.0] | 0,208858791 | 35.2[21.0 : 51.1] | **1,39E-06** | 57.6[32.2 : 87.8] | **4,64E-06** | 54.9[35.7 : 76.8] | **1,27E-09** | 20.6[-4.1 : 51.8] | 1,313088446 | 90.9[53.1 : 137.9] | **1,14E-07** | -9.5[-28.3 : 14.2] | 4,776278952 | 20.8[9.2 : 33.7] | **0,003053762** | Proinflammatory |
| IL-1A | 1.3[-6.9 : 10.1] | 9,246930439 | -8.2[-16.9 : 1.3] | 1,077384791 | -8.4[-21.3 : 6.6] | 3,061827361 | 1.9[-9.3 : 14.4] | 9,08151928 | -10.8[-26.9 : 8.8] | 3,124828262 | -5.2[-21.6 : 14.5] | 6,948321626 | 0.0[-18.7 : 22.9] | 11,95505506 | 5.2[-3.8 : 15.1] | 3,217998439 | Proinflammatory |
| IL-1B | -1.2[-14.9 : 14.8] | 10,53919399 | 2.8[-13.9 : 22.8] | 9,101856127 | 2.1[-22.2 : 33.9] | 10,5900506 | 1.2[-17.8 : 24.5] | 10,96804643 | -28.0[-49.5 : 2.8] | 0,849603635 | 36.9[-2.5 : 92.2] | 0,835925819 | 1.6[-29.8 : 47.0] | 11,21642518 | 14.8[-2.2 : 34.8] | 1,103592109 | Proinflammatory |
| IL-1RA | 5.1[-0.5 : 11.1] | 0,889715735 | -3.3[-9.3 : 3.2] | 3,801445429 | 0.5[-9.0 : 11.0] | 11,06681051 | 3.8[-3.8 : 12.0] | 3,995185095 | -15.0[-25.4 : -3.0] | 0,185567409 | -5.4[-16.5 : 7.1] | 4,579015748 | -7.3[-19.0 : 6.1] | 3,243396477 | 3.5[-2.5 : 9.7] | 3,079940232 | Proinflammatory |
| IL-3 | 10.9[-1.4 : 24.7] | 1,029316631 | 0.2[-12.8 : 15.1] | 11,70912045 | 0.3[-18.9 : 24.0] | 11,7497155 | 4.7[-11.1 : 23.2] | 7,004312909 | -14.4[-35.2 : 13.1] | 3,299238575 | -14.9[-34.9 : 11.1] | 2,824911508 | -4.1[-28.4 : 28.4] | 9,330475976 | 10.8[-2.3 : 25.7] | 1,337906613 | Proinflammatory |
| IL-6 | -6.5[-14.5 : 2.2] | 1,630286695 | -9.2[-18.3 : 0.8] | 0,849076817 | -0.9[-15.6 : 16.4] | 10,95719611 | -0.9[-12.4 : 12.1] | 10,63964052 | -2.6[-21.1 : 20.3] | 9,689932274 | 9.7[-10.3 : 34.1] | 4,411591258 | -3.8[-22.7 : 19.8] | 8,774546157 | 23.9[12.6 : 36.2] | **1,28E-04** | Proinflammatory |
| IL-12 | -0.4[-5.4 : 4.8] | 10,37750732 | 0.6[-5.2 : 6.9] | 10,02654277 | -0.7[-9.4 : 8.9] | 10,5657774 | 8.6[1.3 : 16.6] | 0,252955281 | -5.0[-15.8 : 7.2] | 4,837139492 | 5.9[-5.5 : 18.8] | 3,889663382 | 6.5[-6.1 : 20.7] | 3,935077363 | 8.4[2.7 : 14.5] | **0,042541279** | Proinflammatory |
| IL-12p70 | 6.1[-7.2 : 21.2] | 4,626034807 | -14.5[-27.0 : 0.1] | 0,614364006 | -9.8[-29.1 : 14.8] | 4,83791226 | -0.5[-17.3 : 19.6] | 11,45305799 | 10.1[-19.7 : 51.0] | 6,586150264 | 0.4[-25.7 : 35.7] | 11,74553429 | -15.6[-39.2 : 17.1] | 3,710189987 | 8.7[-5.7 : 25.4] | 2,982269038 | Proinflammatory |
| IL-15 | -4.1[-6.6 : -1.6] | **0,017412618** | 1.2[-1.9 : 4.3] | 5,379695131 | -2.8[-7.3 : 1.8] | 2,775083217 | 3.8[0.1 : 7.5] | 0,514821321 | 2.2[-3.9 : 8.6] | 5,898988092 | 5.2[-0.8 : 11.5] | 1,057610418 | -3.6[-9.6 : 2.7] | 3,104747467 | 0.3[-2.5 : 3.1] | 10,310753 | Proinflammatory |
| TNF-A | -2.2[-5.4 : 1.1] | 2,293667629 | -1.2[-5.0 : 2.8] | 6,696282396 | -3.8[-9.4 : 2.1] | 2,368728614 | -0.8[-5.3 : 3.8] | 8,613211379 | -6.0[-13.1 : 1.6] | 1,44541628 | -1.4[-8.5 : 6.3] | 8,541541667 | -2.4[-10.0 : 5.8] | 6,647730328 | 10.0[6.2 : 14.0] | **1,49E-06** | Proinflammatory |
| TNF-B | -4.6[-11.7 : 3.1] | 2,802075714 | -10.0[-17.8 : -1.4] | 0,288908065 | -5.3[-17.7 : 8.8] | 5,289136311 | -0.1[-10.2 : 11.2] | 11,8762285 | -3.3[-19.4 : 16.1] | 8,645573229 | -4.3[-19.6 : 13.9] | 7,43066517 | -3.0[-19.8 : 17.3] | 9,036060888 | 7.0[-1.4 : 16.2] | 1,270784824 | Proinflammatory |
| IFN-G | -3.0[-10.3 : 5.0] | 5,871425576 | 6.6[-2.9 : 17.0] | 2,355390366 | 13.3[-1.7 : 30.7] | 1,10864222 | 11.5[0.0 : 24.3] | 0,658021352 | 19.5[-0.9 : 44.0] | 0,801940022 | 5.2[-12.0 : 25.7] | 7,50438923 | -6.0[-22.6 : 14.1] | 6,882269842 | 9.2[0.4 : 18.8] | 0,513063926 | T derived |
| IL-2 | 20.5[5.2 : 38.1] | 0,094150868 | 6.9[-9.0 : 25.6] | 5,398660963 | 13.7[-11.1 : 45.4] | 3,973018852 | -7.8[-23.6 : 11.3] | 5,175972452 | 46.0[5.7 : 101.6] | 0,279165654 | 2.0[-25.0 : 38.7] | 11,68242167 | -3.7[-31.1 : 34.6] | 10,73257453 | 12.2[-3.0 : 29.7] | 1,588526896 | T derived |
| IL-4 | 0.3[-11.7 : 13.9] | 12,54261803 | 8.3[-6.8 : 25.9] | 3,873309153 | -1.3[-21.6 : 24.3] | 11,87741546 | -4.5[-19.9 : 13.9] | 7,929021879 | 6.8[-21.0 : 44.4] | 8,700390347 | 21.3[-9.0 : 61.7] | 2,450783643 | -14.4[-37.4 : 17.1] | 4,310343318 | 6.0[-7.5 : 21.5] | 5,195041279 | T derived |
| IL-5 | 1.9[-5.0 : 9.2] | 7,84330497 | 2.3[-5.8 : 11.1] | 7,696181946 | -1.5[-13.1 : 11.8] | 10,65687157 | 14.3[3.8 : 25.9] | 0,086233783 | -3.5[-18.2 : 13.9] | 8,770388319 | 15.5[-1.3 : 35.1] | 0,951470295 | -13.9[-27.5 : 2.2] | 1,134737609 | -1.4[-8.5 : 6.3] | 9,276000663 | T derived |
| IL-9 | 5.5[-7.4 : 20.3] | 5,465468905 | -16.1[-28.1 : -2.1] | 0,337350528 | -11.9[-30.6 : 11.7] | 3,819214314 | -2.8[-18.9 : 16.6] | 9,88348224 | -5.4[-30.8 : 29.4] | 9,480603137 | 19.6[-11.2 : 61.1] | 3,090590826 | -10.9[-35.4 : 23.0] | 6,285293668 | 20.6[4.8 : 38.8] | 0,115329374 | T derived |
| IL-10 | -1.0[-9.6 : 8.5] | 10,80899891 | -3.8[-13.7 : 7.1] | 6,212110683 | -19.3[-31.6 : -4.8] | 0,142385825 | 1.8[-10.3 : 15.5] | 10,22266009 | 17.8[-5.2 : 46.2] | 1,801105656 | -9.9[-26.7 : 10.7] | 4,161002498 | 11.5[-10.9 : 39.6] | 4,444660077 | 9.7[-0.5 : 20.9] | 0,825748509 | T derived |
| IL-13 | 4.1[-7.0 : 16.5] | 6,303119379 | 1.8[-10.9 : 16.3] | 10,27565394 | -6.7[-23.9 : 14.4] | 6,552502571 | -5.0[-18.7 : 11.0] | 6,743974944 | 18.9[-8.9 : 55.3] | 2,643990522 | 4.1[-19.3 : 34.3] | 9,832490664 | -4.9[-27.9 : 25.5] | 9,417215192 | -7.1[-17.6 : 4.8] | 2,99644495 | T derived |
| IL-16 | 5.0[-1.7 : 12.1] | 1,874824442 | -8.1[-14.9 : -0.7] | 0,410875182 | -10.8[-20.8 : 0.4] | 0,756717659 | -6.4[-14.5 : 2.4] | 1,947099919 | -15.7[-27.8 : -1.7] | 0,387098455 | -7.1[-19.9 : 7.7] | 4,27145298 | -2.4[-16.9 : 14.6] | 9,946806154 | 6.5[-0.7 : 14.2] | 0,983592723 | T derived |
| IL-17A | 0.6[-6.0 : 7.6] | 11,27661881 | 3.4[-4.5 : 12.0] | 5,353260571 | 1.5[-10.1 : 14.7] | 10,53748807 | 8.5[-1.2 : 19.1] | 1,120538114 | 3.2[-12.1 : 21.0] | 9,1454598 | 7.5[-7.6 : 25.2] | 4,537943438 | 2.9[-12.9 : 21.5] | 9,609405581 | 9.8[2.1 : 18.0] | 0,14673197 | T derived |
| IL-17A/F | -3.4[-13.4 : 7.9] | 7,026309954 | 3.1[-9.4 : 17.4] | 8,324893675 | 12.1[-8.2 : 36.8] | 3,395887977 | 3.7[-10.9 : 20.8] | 8,308464938 | 5.9[-18.5 : 37.7] | 8,683973222 | 25.7[-2.0 : 61.4] | 0,935901558 | -23.4[-41.5 : 0.4] | 0,692057297 | -4.0[-14.7 : 8.0] | 6,39836949 | T derived |
| IL-17B | -9.4[-17.6 : -0.4] | 0,538682281 | -17.3[-26.0 : -7.4] | **0,012298429** | -12.3[-26.1 : 4.1] | 1,728816489 | -16.0[-26.3 : -4.2] | 0,118380183 | -28.2[-42.6 : -10.2] | 0,048711218 | -6.3[-24.3 : 15.9] | 7,109889107 | -20.9[-37.4 : 0.1] | 0,659011037 | 12.8[1.9 : 24.8] | 0,264699467 | T derived |
| IL-17C | -7.3[-16.6 : 3.1] | 2,105632232 | -14.8[-24.9 : -3.5] | 0,154666904 | -4.0[-20.7 : 16.3] | 8,802542684 | 11.5[-3.7 : 29.2] | 1,886619422 | -2.7[-24.4 : 25.1] | 10,77556481 | -9.3[-28.6 : 15.3] | 5,5435731 | -28.3[-44.8 : -6.9] | 0,161693504 | 2.6[-8.4 : 15.0] | 8,498729104 | T derived |
| IL-17D | -2.8[-11.6 : 6.9] | 7,301246051 | 6.1[-5.1 : 18.7] | 3,897822173 | 15.4[-2.8 : 37.0] | 1,326216465 | 0.1[-12.2 : 14.1] | 12,8858124 | -11.6[-29.4 : 10.7] | 3,696160888 | -10.1[-27.4 : 11.4] | 4,299781388 | -8.0[-27.1 : 16.2] | 6,294907576 | 16.5[5.3 : 29.0] | **0,041432848** | T derived |
| CCL2 | -1.1[-5.6 : 3.5] | 6,321027108 | 1.0[-4.4 : 6.7] | 7,250570806 | 3.2[-5.3 : 12.5] | 4,738991538 | 9.1[2.2 : 16.5] | 0,086769746 | -12.6[-22.0 : -2.2] | 0,190077994 | 17.0[4.9 : 30.4] | **0,046807343** | -1.3[-12.1 : 10.8] | 8,211110958 | 15.7[10.2 : 21.6] | **7,30E-08** | Chemokine |
| CCL3 | -1.4[-6.0 : 3.4] | 5,612384501 | 4.5[-1.3 : 10.6] | 1,29635689 | -4.4[-12.7 : 4.6] | 3,255754976 | 6.7[-0.3 : 14.3] | 0,608908998 | -3.6[-14.3 : 8.5] | 5,436325397 | 11.2[-0.7 : 24.5] | 0,666470928 | 4.7[-7.2 : 18.1] | 4,536352951 | 8.3[2.9 : 14.1] | **0,02408831** | Chemokine |
| CCL4 | -2.9[-7.9 : 2.3] | 2,659581906 | -6.4[-12.1 : -0.3] | 0,394266685 | -4.1[-13.2 : 5.9] | 4,030776544 | -5.2[-12.0 : 2.2] | 1,663970966 | -8.4[-19.5 : 4.2] | 1,833828368 | -2.5[-13.9 : 10.5] | 6,929509069 | -9.9[-21.1 : 2.9] | 1,241870586 | 8.1[2.1 : 14.4] | 0,073684749 | Chemokine |
| CCL11 | -2.9[-6.9 : 1.2] | 1,662451656 | -9.6[-14.0 : -5.0] | **7,06E-04** | -12.4[-19.0 : -5.2] | **0,010741393** | -4.3[-9.8 : 1.6] | 1,521429728 | -19.2[-27.1 : -10.4] | **5,36E-04** | -6.5[-15.3 : 3.3] | 1,84859741 | 3.2[-7.2 : 14.7] | 5,602105722 | 7.3[2.6 : 12.3] | **0,021899044** | Chemokine |
| CCL13 | 0.2[-5.1 : 5.7] | 9,544653844 | -15.9[-21.1 : -10.4] | **8,88E-07** | -10.8[-19.4 : -1.4] | 0,254171035 | -7.9[-14.7 : -0.7] | 0,329961774 | -15.0[-25.5 : -3.1] | 0,150579411 | -1.9[-13.6 : 11.3] | 7,626996678 | -12.1[-23.1 : 0.6] | 0,605686457 | 5.0[-0.9 : 11.2] | 0,967326413 | Chemokine |
| CCL17 | 1.1[-6.2 : 8.9] | 7,798342228 | -14.8[-22.0 : -6.9] | **0,004187602** | -26.7[-36.3 : -15.6] | **1,57E-04** | -18.6[-26.8 : -9.4] | **0,001519924** | -43.8[-53.2 : -32.5] | **7,77E-09** | -17.4[-30.7 : -1.4] | 0,339324746 | -3.2[-19.8 : 16.7] | 7,314526896 | 20.0[10.7 : 30.0] | **9,01E-05** | Chemokine |
| CCL22 | -1.4[-4.8 : 2.2] | 4,472487439 | -0.6[-4.7 : 3.7] | 7,747498751 | 2.8[-3.9 : 9.9] | 4,217760269 | 5.6[0.4 : 11.1] | 0,332945689 | -12.5[-19.8 : -4.6] | **0,02621137** | 15.5[6.2 : 25.6] | **0,007411933** | -6.1[-14.1 : 2.7] | 1,694130793 | 16.0[11.7 : 20.5] | **3,09E-13** | Chemokine |
| CCL26 | -0.9[-8.1 : 6.8] | 8,071445944 | -8.2[-16.0 : 0.4] | 0,61087131 | -10.4[-22.2 : 3.3] | 1,299077972 | 4.0[-6.5 : 15.8] | 4,707521187 | -24.2[-37.0 : -8.9] | **0,032334011** | 3.0[-13.8 : 23.0] | 7,46053771 | -11.4[-26.7 : 7.0] | 2,075236119 | -3.6[-11.1 : 4.5] | 3,687353577 | Chemokine |
| IL-8 | 0.7[-7.1 : 9.1] | 8,690013991 | 12.6[2.4 : 23.7] | 0,143780313 | 6.4[-8.0 : 22.9] | 4,029325426 | 5.1[-5.9 : 17.4] | 3,752411273 | -12.6[-27.7 : 5.7] | 1,649895466 | 2.7[-14.3 : 23.1] | 7,706915039 | -8.1[-24.6 : 11.9] | 3,989903276 | 4.2[-4.4 : 13.5] | 3,506453119 | Chemokine |
| CXCL10 | 0.7[-4.8 : 6.5] | 8,008972731 | -3.2[-9.4 : 3.4] | 3,354140331 | -6.4[-15.8 : 4.0] | 2,156038335 | -1.2[-8.7 : 7.0] | 7,735843287 | -19.4[-29.8 : -7.6] | **0,020526943** | -7.7[-19.1 : 5.4] | 2,374902212 | -1.9[-14.8 : 12.9] | 7,864215783 | 11.6[5.1 : 18.5] | **0,003575938** | Chemokine |
| bFGF | -2.5[-10.8 : 6.7] | 7,049705978 | 13.9[2.4 : 26.7] | 0,19455339 | 16.7[-0.8 : 37.2] | 0,751344389 | 14.2[0.8 : 29.3] | 0,441929102 | -5.3[-23.3 : 17.0] | 7,378172351 | 8.3[-11.6 : 32.6] | 5,289991707 | -14.5[-31.5 : 6.7] | 1,984974012 | 6.0[-3.7 : 16.7] | 2,82803668 | Growth Factor |
| Flt-1 | -1.2[-4.4 : 2.2] | 5,864473247 | -11.2[-14.6 : -7.7] | **3,19E-08** | -21.1[-25.6 : -16.2] | **1,27E-13** | -13.1[-17.0 : -9.0] | **2,71E-08** | -26.4[-31.9 : -20.4] | **1,87E-13** | -20.3[-26.0 : -14.1] | **3,65E-08** | 4.1[-4.1 : 12.9] | 4,027480586 | -0.5[-3.9 : 3.1] | 9,629675959 | Growth Factor |
| GM-CSF | -1.7[-11.2 : 8.9] | 8,905268186 | -6.7[-17.3 : 5.3] | 3,150357689 | 7.1[-10.9 : 28.8] | 5,589119 | -12.9[-24.4 : 0.3] | 0,660057041 | -8.4[-28.1 : 16.6] | 5,700378013 | -9.8[-28.3 : 13.6] | 4,575136693 | -2.3[-24.0 : 25.7] | 10,30398468 | 10.6[-0.8 : 23.4] | 0,837756792 | Growth Factor |
| IL-7 | -5.9[-10.8 : -0.7] | 0,327498664 | 13.6[6.6 : 21.1] | **0,001092709** | 8.4[-1.6 : 19.6] | 1,236722594 | 13.9[5.7 : 22.7] | **0,007335275** | 2.1[-10.2 : 16.0] | 9,011929958 | 14.6[1.5 : 29.4] | 0,335758379 | -8.1[-19.6 : 5.0] | 2,54720194 | -0.6[-6.1 : 5.3] | 10,17009598 | Growth Factor |
| PlGF | -0.6[-3.2 : 2.1] | 8,001386261 | 1.7[-1.5 : 5.0] | 3,622029626 | 2.1[-2.7 : 7.2] | 4,786224828 | 0.3[-3.4 : 4.1] | 10,39737284 | 0.9[-5.3 : 7.5] | 9,354545889 | -0.1[-6.0 : 6.2] | 11,76287677 | -3.9[-10.1 : 2.7] | 2,862418782 | 8.9[5.8 : 12.1] | **7,72E-08** | Growth Factor |
| sICAM-1 | -1.4[-4.8 : 2.2] | 5,44944492 | -0.2[-4.3 : 4.1] | 11,16735701 | 4.3[-2.4 : 11.5] | 2,566772702 | 3.5[-1.6 : 8.9] | 2,154906539 | 1.9[-6.6 : 11.3] | 7,983052325 | 6.8[-1.8 : 16.1] | 1,513046131 | -1.5[-9.8 : 7.7] | 8,940487135 | 14.3[9.9 : 18.7] | **1,64E-10** | Growth Factor |
| sVCAM-1 | 1.2[-1.6 : 4.2] | 4,73572918 | -13.7[-16.6 : -10.8] | **9,62E-17** | -9.2[-13.8 : -4.2] | **0,004313431** | -7.1[-10.8 : -3.4] | **0,003277111** | -11.7[-17.6 : -5.4] | **0,005084769** | -7.2[-13.1 : -0.8] | 0,330630065 | -0.8[-7.5 : 6.4] | 9,779356669 | 7.0[3.8 : 10.3] | **1,49E-04** | Growth Factor |
| Tie-2 | -1.7[-4.4 : 1.2] | 2,993502465 | -6.8[-9.9 : -3.6] | **5,77E-04** | -3.0[-7.9 : 2.1] | 2,917031199 | -3.6[-7.3 : 0.3] | 0,83710473 | -13.2[-18.9 : -7.2] | **4,20E-04** | -2.4[-8.5 : 4.1] | 5,571064367 | 6.3[-0.9 : 14.0] | 1,048049092 | 4.0[0.9 : 7.2] | 0,139153741 | Growth Factor |
| TSLP | -15.8[-24.5 : -6.0] | **0,025126496** | -6.0[-17.4 : 6.9] | 4,152995524 | 0.4[-17.6 : 22.3] | 11,60045406 | 11.3[-4.3 : 29.4] | 1,979699064 | -30.4[-46.3 : -9.9] | 0,072036624 | 10.6[-13.5 : 41.5] | 5,06816978 | -8.1[-29.7 : 20.2] | 6,456219058 | 15.3[2.6 : 29.6] | 0,202291264 | Growth Factor |
| VEGF | -1.0[-10.1 : 9.1] | 10,06648829 | 32.6[18.2 : 48.7] | **1,78E-05** | 38.9[16.6 : 65.5] | **0,002820891** | 28.8[12.6 : 47.3] | **0,002700654** | 40.9[12.1 : 77.0] | **0,039339647** | 55.1[24.6 : 93.0] | **0,001043301** | -2.2[-23.0 : 24.2] | 10,26784989 | 7.2[-3.4 : 18.9] | 2,29382566 | Growth Factor |
| VEGF-C | -1.8[-7.1 : 3.7] | 6,107672751 | 10.8[3.9 : 18.3] | **0,023424088** | 23.3[11.7 : 36.2] | **4,30E-04** | 12.1[3.9 : 21.0] | **0,039453538** | 5.1[-7.7 : 19.6] | 5,442175232 | 21.1[7.0 : 37.1] | **0,030214807** | 3.9[-9.3 : 19.0] | 6,969789051 | 5.5[-0.6 : 11.8] | 0,922009499 | Growth Factor |
| VEGF-D | -5.1[-10.9 : 1.1] | 1,283134579 | 8.7[0.9 : 17.2] | 0,338914094 | 55.4[38.7 : 74.2] | **5,05E-13** | 38.2[26.6 : 50.8] | **6,02E-12** | 42.1[22.4 : 64.8] | **4,51E-05** | 64.0[42.2 : 89.1] | **1,43E-10** | -4.8[-18.5 : 11.3] | 6,473294268 | 2.2[-4.5 : 9.3] | 6,390950125 | Growth Factor |

**Supplementary Table 12:** Model with ATC codes as exposure instead of group. Estimates are shown as percentage change and p values are corrected for multiple testing. Model is adjusted for age, current smoking, BMI, sample age, sample analysis date, and Danish administrative region of sampling. ATC codes with less than 50 users are excluded. In the top row the n for each ATC code is noted.

| **Supplementary Table 13** | |  |  |
| --- | --- | --- | --- |
|  | Hormone therapy |  |  |
| Assay | % estimate | corrected P | group |
| CRP | 13.1[-10.9 : 43.6] | 3,7456483 | Proinflammatory |
| SAA | 14.7[-5.8 : 39.6] | 2,0625544 | Proinflammatory |
| IL-1A | -1.7[-18.8 : 19.0] | 10,3479620 | Proinflammatory |
| IL-1B | -12.8[-37.2 : 21.1] | 4,9723465 | Proinflammatory |
| IL-1RA | -7.3[-17.7 : 4.5] | 2,5743766 | Proinflammatory |
| IL-3 | -32.2[-47.8 : -11.8] | 0,0461331 | Proinflammatory |
| IL-6 | -11.6[-26.4 : 6.1] | 2,2060521 | Proinflammatory |
| IL-12 | 2.7[-8.1 : 14.8] | 7,6447640 | Proinflammatory |
| IL-12p70 | 6.0[-20.7 : 41.6] | 8,3351823 | Proinflammatory |
| IL-15 | -0.4[-5.6 : 5.0] | 10,5349653 | Proinflammatory |
| TNF-A | 4.6[-2.9 : 12.7] | 2,8261693 | Proinflammatory |
| TNF-B | -3.1[-19.3 : 16.4] | 8,8488678 | Proinflammatory |
| IFN-G | -1.4[-17.4 : 17.5] | 11,3226753 | T derived |
| IL-2 | 53.2[13.1 : 107.7] | 0,0776775 | T derived |
| IL-4 | 12.5[-15.9 : 50.6] | 5,5485237 | T derived |
| IL-5 | 8.6[-6.3 : 25.9] | 3,5718672 | T derived |
| IL-9 | -9.4[-32.3 : 21.1] | 6,5344158 | T derived |
| IL-10 | 4.6[-15.2 : 29.0] | 8,7917632 | T derived |
| IL-13 | -1.4[-22.5 : 25.4] | 11,8230330 | T derived |
| IL-16 | -2.7[-15.3 : 11.9] | 9,1687568 | T derived |
| IL-17A | 11.3[-5.2 : 30.6] | 2,4909149 | T derived |
| IL-17A/F | -6.9[-27.8 : 20.0] | 7,5396979 | T derived |
| IL-17B | 0.0[-18.9 : 23.3] | 12,9703788 | T derived |
| IL-17C | -1.1[-22.3 : 26.0] | 12,1003685 | T derived |
| IL-17D | -16.2[-29.6 : -0.2] | 0,6107782 | T derived |
| CCL2 | 0.9[-9.3 : 12.2] | 8,7458727 | Chemokine |
| CCL3 | 1.1[-8.4 : 11.6] | 8,2641414 | Chemokine |
| CCL4 | 1.8[-9.2 : 14.0] | 7,6366781 | Chemokine |
| CCL11 | -6.3[-14.6 : 2.7] | 1,6482830 | Chemokine |
| CCL13 | 0.4[-10.6 : 12.8] | 9,4666281 | Chemokine |
| CCL17 | -4.1[-18.4 : 12.6] | 6,0746271 | Chemokine |
| CCL22 | 3.8[-3.8 : 12.1] | 3,3768962 | Chemokine |
| CCL26 | 8.2[-9.3 : 29.1] | 3,7927853 | Chemokine |
| IL-8 | -4.4[-20.3 : 14.8] | 6,3261519 | Chemokine |
| CXCL10 | -2.1[-13.4 : 10.8] | 7,4018216 | Chemokine |
| bFGF | 3.7[-14.8 : 26.1] | 8,6072875 | Growth Factor |
| Flt-1 | -7.3[-13.4 : -0.7] | 0,3604640 | Growth Factor |
| GM-CSF | 2.5[-18.6 : 29.0] | 10,0318885 | Growth Factor |
| IL-7 | -3.4[-13.7 : 8.0] | 6,4829910 | Growth Factor |
| PlGF | 0.0[-5.6 : 6.0] | 11,8804089 | Growth Factor |
| sICAM-1 | 0.5[-5.4 : 6.9] | 10,3320257 | Growth Factor |
| sVCAM-1 | 3.6[-1.7 : 9.1] | 2,1985424 | Growth Factor |
| Tie-2 | 0.0[-5.9 : 6.2] | 11,9220607 | Growth Factor |
| TLSP | -15.3[-32.3 : 5.8] | 1,7138547 | Growth Factor |
| VEGF | -2.5[-21.1 : 20.6] | 9,8096542 | Growth Factor |
| VEGF-C | -4.7[-15.6 : 7.5] | 5,1486189 | Growth Factor |
| VEGF-D | 8.2[-6.4 : 25.1] | 3,4177482 | Growth Factor |

**Supplementary Table 13** Model with hormone therapy users compared to the menopausal group. Estimates are shown as percentage change and p values are corrected for multiple testing. Model is adjusted for age, current smoking, BMI, sample age, sample analysis date, and Danish administrative region of sampling.
