# Supplementary material for: Effect of Hormonal Contraceptives on Circulating Biomarkers of Inflammation, Chemotaxis, Angiogenesis, and Vascular Stress
Source: APMIS. 2026 Jul 14;134(7):e70235. doi: 10.1111/apm.70235 (PMC13368256; doi:10.1111/apm.70235)
Supplement: Supplementary file 7 — Supinfo1: [file APM-134-0-s004.docx]

# Supplementary methods

**Biomarker measurements**

In brief, the plasma samples were retrieved from the biobank analyzed as singlets with an internal control added in duplicate on each plate. Measurements below detection range were assigned a concentration between zero and the lower detection limit drawn from a uniform distribution. Measurements above detection range were set to the upper detection limit. Concentrations were median normalized by dividing the concentration with the plate median and multiplying with the overall median, as previously described(20).

**Assay aliases:**

bFGF (Basic Fibroblast growth factor, FGF2, HBGF-2, Uniprot:P09038)

CRP (C reactive protein, PTX1, Uniprot:P02741)

Eotaxin (CCL11, Uniprot:P51671)

Eotaxin-3 (CCL26, MIP-4α, IMAC, Uniprot:Q9Y258)

FLT1 (Vascular Endothelial Growth Factor Receptor 1, Uniprot:P17948)

GM-CSF (Granulocyte-macrophage colony-stimulating factor, CSF2, Uniprot:P04141)

IFNγ (Interferon gamma, IFNG, IFG, IFI, Uniprot:P01579)

IL1α (Interleukin 1 alpha, IL1A, IL1F1, Uniprot:P01583)

IL1β (Interleukin 1 beta, IL1B, IL1F2, Uniprot:P01584)

IL1RA (Interleukin-1 receptor antagonist, IL1RN, DIRA, Uniprot:P18510)

IL2 (Interleukin-2, TCGF, Uniprot:P60568)

IL3 (Interleukin-3, MCGF, MULTI-CSF, Uniprot:P08700)

IL4 (Interleukin-4, 4BSF, Uniprot:P05112)

IL5 (Interleukin-5, Uniprot:P05113)

IL6 (Interleukin-6, BSF2, HGF, HSF, IFNB2, Uniprot:P05231)

IL7 (Interleukin-7, Uniprot:P13232)

IL8 (Interleukin-8, CXCL8, GCP-1, LECT, LUCT, LYNAP, MDNCF, Uniprot:P10145)

IL9 (Interleukin-9, HP40, Uniprot:P15248)

IL10 (Interleukin-10, CSIF, GVHDS, Uniprot:P22301)

IL12/IL23-p40 (Interleukin-12 subunit beta, CLMF, IL12B, NKSF, Uniprot:P29460)

IL12p70 (Interleukin-12, Uniprot:P29459)

IL13 (Interleukin-13, P600, Uniprot:P35225)

IL15 (Interleukin-15, Uniprot:P40933)

IL16 (Interleukin-16, LCF, NPRprIL-16, Uniprot:Q14005)

IL17A (Interleukin-17A, CTLA8, Uniprot:Q16552)

IL17A/F (IL17F, Interleukin-17A and F heterodimer, Uniprot:Q96PD4)

IL17B (Interleukin-17B, Uniprot:Q9UHF5)

IL17C (Interleukin-17C, Uniprot:Q9P0M4)

IL17D (Interleukin-17D, Uniprot:Q8TAD2)

IP10 (Interferon gamma-induced protein 10, CXCL10, IFI10, SCYB10, Uniprot:P02778)

MCP1 (monocyte chemoattractant protein 1, CCL2, GDCF-2, HC11, Uniprot:P13500)

MCP4 (monocyte chemoattractant protein 4, CCL13, Uniprot:Q99616)

MDC (macrophage derived chemokine, CCL22, ABCD-1, Uniprot:O00626)

MIP1α (macrophage inflammatory protein 1α, SCYA3, CCL3, Uniprot:P10147)

MIP1β (macrophage inflammatory protein 1β, SCYA4, CCL4, LAG1, Uniprot:P13236)

PlGF (Placental growth factor, PGF, Uniprot:P49763)

SAA (Serum amyloid A, Uniprot:P0DJI8/9)

sICAM1 (soluble intracellular adhesion molecule 1, CD54, BB2, Uniprot:Q99930/P05362)

sVCAM1 (soluble Vascular cell adhesion protein 1, CD106, INCAM-100, Uniprot:P19320)

TARC (thymus- and activation-regulated chemokine, CCL17, ABCD-2, SCYA17, Uniprot:Q92583)

TIE2 (TEK tyrosine kinase, CD202B, CMCM, Uniprot:Q02763)

TSLP (thymic stromal lymphopoietin, Uniprot:Q969D9)

TNFα (Tumor Necrosis Factor-alpha, TNF, DIF, TNFSF2, TNLG1F, Uniprot:P01375)

TNFβ (Tumor Necrosis Factor-beta, Lymphotoxin alpha, LTA, TNFSF1, TNLG1E, Uniprot:P01374)

VEGFA (Vascular endothelial growth factor A, MVCD1, VPF, Uniprot:P15692)

VEGFC (Vascular endothelial growth factor C, Flt-4L, LMPH1D, VRP, Uniprot:P49767)

VEGFD (Vascular endothelial growth factor D, FIGF, c-fos induced growth factor, Uniprot:O43915)

| **Classification af combined oral contraceptives** | | | | | |
| --- | --- | --- | --- | --- | --- |
| **Generation** | **Anatomical Therapeutic Chemical code and trade name** | **Progestin** | **Estrogen** | **Group** | **Duration** |
| **2nd generation** | G03AA07 » Anastrella®, komb. | 150 µg levonorgestrel | 30 µg | High | Volume |
|  | G03AA07 » Etnor |  |  | High |  |
|  | G03AA07 » Femicept, komb. |  |  | high |  |
|  | G03AA07 » Loette® 28, komb. | 100 µg levonorgestrel | 20 µg | Low | Volume |
|  | G03AA07 » Malonetta, komb. | 150 µg levonorgestrel | 30 µg | High | Volume |
|  | G03AA07 » Microgyn®, komb. |  |  | High |  |
|  | G03AA07 » Microstad, komb. |  |  | High |  |
|  | G03AA07 » Rigevidon, komb. |  |  | High |  |
|  | G03AA11 » Cilest®, komb. | 250 µg levonorgestrel/Norgestimate | 35 µg | High | Volume |
|  | G03AA11 » Liberelle®, komb. |  |  |  |  |
|  | G03AA11 » Amorina®, komb. |  |  |  |  |
|  | G03AB03 » Trinordiol®, multiphase | 50–125 µg levonorgestrel | 30–40 µg | High | Volume |
|  | G03AB03 » Triregol, multiphase |  |  | High |  |
|  | G03AB04 » Synphase, multiphase | 0.5–1 norethisterone | 35 µg | High | Volume |
| **3rd generation** | G03AA09 » Daisynelle®, komb. | 150 µg desogestrel | 30 µg | High | Volume |
|  | G03AA09 » Denise®, komb. |  |  | High |  |
|  | G03AA09 » Marvelon®, komb. |  |  | High |  |
|  | G03AB05 » Gracial, komb |  |  | High |  |
|  | G03AA09 » Femistad, komb. |  | 20 µg | Low | Volume |
|  | G03AA09 » Mercilon®, komb. |  |  | Low |  |
|  | G03AA10 » Gestinyl®, komb. | 75 µg gestoden | 20/30 µg | Low | Volume |
|  | G03AA10 » Gestodilat®, komb. |  |  | Low |  |
|  | G03AA10 » Harmonet®, komb. |  | 20 µg | Low | Volume |
|  | G03AA10 » Minero, komb. |  |  | Low |  |
|  | G03AA10 » Minulet®, komb. |  | 30 µg | High | Volume |
|  | G03AA10 » Modina, komb. |  |  | High |  |
| **4th generation** | G03AA12 » Cleosensa, komb. | 3000 µg drospirenon | 20 µg | Low | Volume |
|  | G03AA12 » Stefaminelle®, komb. |  |  | Low |  |
|  | G03AA12 » Veyann®, komb. |  |  | Low |  |
|  | G03AA12 » Yasminelle 28®, komb. |  |  | Low |  |
|  | G03AA12 » Yasminelle®, komb. |  |  | Low |  |
|  | G03AA12 » Yaz®, komb. |  |  | Low |  |
|  | G03AA12 » Dretine®, komb. |  | 20/30 µg | Low | Volume |
|  | G03AA12 » Finminette®, komb. |  |  | Low |  |
|  | G03AA12 » Yasmin 28®, komb. |  | 30 µg | High | Volume |
|  | G03AA12 » Yasmin®, komb. |  |  | High |  |
| **Unclassified** | G03AA14 » Zoely | 2.5 mg nomegestrolacetate | 1.5 mg estradiol as hemihydrate | Low | Volume |
|  | G03AA16 » Yana, komb. | 2 mg dienogest | 30µg | High |  |
|  | G03AB08 » Qlaira®, multiphase. | 0–3 mg dienogest | 1–3 mg estradiolvalerate | Low |  |
| **POP** | G03AC01 » Mini-Pe® | 0.35 mg norethisterone | NA | POP |  |
|  | G03AC02 » Exluton® | 0.5 mg lynestrenol |  |  |  |
|  | G03AC03 | Levonorgestrel |  |  |  |
|  | G03AC06 »Depo-Provera® | 50 mg /mL medroxyprogesteronacetate |  |  |  |
|  | G03AC08 »Nexplanon | 68 mg etonogestrel/implant |  |  |  |
|  | G03AC09 »Azalia | 75 mg desogestrel |  |  | Volume |
|  | G03AC09 »Ceranor |  |  |  |  |
|  | G03AC09 »Cerazette |  |  |  |  |
|  | G03AC09 »Delamonie |  |  |  |  |
|  | G03AC09 »Desirett |  |  |  |  |
|  | G03AC09 »Desogestrel ”Stada” |  |  |  |  |
|  | G03AC09 »Vinelle |  |  |  |  |
|  | G03AC10 » Slinda® | 4 mg drospirenon |  |  |  |
| **IUDs** | G02BA03 Intra-uterin device (progesteron) | - | - | IUD | 1826,25 (days) |
| **HT** | G03C » Various | Various | various | HT | Volume |
| **Excluded** | G03AA13 » Evra®, komb. transdermal | 203 µg norelgestromin/24h | 33.9/24h | Excluded | 7 days |
|  | G02BB01 Intravaginal ring | - | - | - | 21 days |
| **Not included** | G03AB06 | - | - | - |  |
